# Supplementary material for: Interventions to promote family member involvement in adult critical care settings: a systematic review
Source: BMJ Open. 2021 Apr 7;11(4):e042556. doi: 10.1136/bmjopen-2020-042556 (PMC8031009; doi:10.1136/bmjopen-2020-042556)
Supplement: Supplementary data [file bmjopen-2020-042556supp001.pdf]

Search strategy as applied in Medline (date last run 16.06.2019)

1. exp Critical Illness/
2. exp Critical Care/
3. exp Intensive Care Units/
4. critical care.mp.
5. critical illness*.mp.
6. intensive care.mp.
7. ICU.mp.
8. ITU.mp.
9. OR/1-8
10. exp Patient Participation/
11. patient involve*.mp.
12. patient engage*.mp.
13. shared decision making.mp.
14. patient participat*.mp.
15. service user involve*.mp.
16. family involve*.mp.
17. family participat*.mp.
18. family engage*.mp.
19. caregiver involve*.mp.
20. caregiver engage*.mp.
21. caregiver participat*.mp.
22. relative involve*.mp.
23. relative engage*.mp.
24. relative participat*.mp.
25. OR/11-24
26. 9 AND 25
