# Supplementary material for: Interventions to promote family member involvement in adult critical care settings: a systematic review
Source: BMJ Open. 2021 Apr 7;11(4):e042556. doi: 10.1136/bmjopen-2020-042556 (PMC8031009; doi:10.1136/bmjopen-2020-042556)
Supplement: Supplementary data [file bmjopen-2020-042556supp002.pdf]

SUPPLEMENTARY FILE

Supplementary Table 1: Search strategy as applied in Medline 1
Supplementary Table 2: Participation in medical rounds interventions 2
Supplementary Table 3: Multi-component family involvement interventions 3
Supplementary Table 4: Discussion based family involvement interventions 3
Supplementary Table 5: Web-based support interventions 5
Supplementary Table 6: Environmental unit changes & Participation in care interventions... 6

Supplementary Table 1: Search strategy as applied in Medline (date last run 16.06.2019)

1.	exp Critical Illness/
2.	exp Critical Care/
3.	exp Intensive Care Units/
4.	critical care.mp.
5.	critical illness*.mp.
6.	intensive care.mp.
7.	ICU.mp.
8.	ITU.mp.
9.	OR/1-8
10.	exp Patient Participation/
11.	patient involve*.mp.
12.	patient engage*.mp.
13.	shared decision making.mp.
14.	patient participat*.mp.
15.	service user involve*.mp.
16.	family involve*.mp.
17.	family participat*.mp.
18.	family engage*.mp.
19.	caregiver involve*.mp.
20.	caregiver engage*.mp.
21.	caregiver participat*.mp.
22.	relative involve*.mp.
23.	relative engage*.mp.
24.	relative participat*.mp.
25.	OR/11-24
26.	9 AND 25

Xyrichis A, *et al. BMJ Open* 2021; 11:e042556. doi: 10.1136/bmjopen-2020-042556

Supplementary File: Outcomes grouped by type of family involvement intervention

Supplementary Table 2: Participation in medical rounds interventions

Study / Intervention / Measure	Allen et al. 2017	Jacobowski et al. 2010	Weber et al. 2018
Intervention	Family on rounds	Family rounds	Family rounds
Communication (improvement, % agree)	Pre: n=49 (100%)<br>Post: n=47 (100%)<br>Change 0% p=0.68<br>RR: 1, CI: 0-0		
Family knowledge	Improvement, % agree<br>Pre: n=146 (35%) | Post: n=374 (88%)<br>Change 52% p<0.001<br>RR: 2.46, CI: 2.14-2.81		
Nurse workload	Reduction, % agree<br>Pre: n=32 (65%) | Post: n=45 (96%)<br>Change 31%<br>RR: 1.46, CI: 1.18-1.81		
Physician satisfaction	Pre: n=4 (80%) | Post: n= 5 (83%)<br>Change 3%<br>RR: 1.04, CI: 0.59-1.83		
Frequency nurse communication		% highest score<br>Pre: 64% | Post: 57%<br>Change: -7% p=0.30<br>RR: 0.89 CI: 0.70-1.12	
Frequency doctor communication		% highest score<br>Pre: 38% | Post: 60%<br>Change: 22% p=0.004<br>RR: 1.60 CI 1.18-2.17	
Included in decision making		% highest score<br>Pre: 66% | Post: 76%<br>Change: 10% p=0.12<br>RR: 1.15 CI 0.96-1.38	
Support in decision making		% highest score<br>Pre: 49% | Post: 69%<br>Change: 20% p=0.005<br>RR: 1.39 CI 1.09-1.79	
Time to ask questions		% highest score<br>Pre: 40% | Post: 23%<br>Change: -17% p=0.02<br>RR: 0.57 CI 0.37-0.90	
FS ICU 24 - Frequency doctor communication			% top scores<br>Control: 43%<br>Intervention: 56%<br>Change: 13% p>0.05<br>RR: 1.31 CI 0.90-1.89
FS ICU 24 - Frequency nurse communication			% top scores<br>Control: 62%<br>Intervention: 78%<br>Change: 16% p>0.05<br>RR: 1.24 CI 0.98-1.58
FS-ICU (24) - Decision making			Mean score (SD)<br>Control: 85.1 (16.3)<br>Intervention: 88.6 (14.6)<br>Change: 3.5 p=0.20<br>CI: -1.98-8.98
FS-ICU (24) - Global score			Mean score (SD)<br>Control: 86.0 (16.0)<br>Intervention: 90.8 (10.7)<br>Change: 4.8 p=0.20<br>CI: -0.12 - 9.72
Support given (emotional)			% top scores<br>Control: 54%<br>Intervention: 71%<br>Change: 17% p>0.05<br>RR: 1.32 CI 0.99-1.75
Coordination of care			% top scores<br>Control: 68%<br>Intervention: 77%<br>Change: 9%<br>RR: 1.13 CI 0.91-1.41

Supplementary Table 3: Multi-component family involvement interventions

Study	Cray 1989	Davidson et al. 2010	Marshall et al. 2016	White et al. 2018
Intervention / Measure	**Family programme**	**Facilitated sensemaking**	**Nutrition intervention**	**Multi family support**
**Satisfaction with family involvement**	100% agreed (76/76)	Observations showed family members were most engaged when receiving information about how to participate at the bedside.	Reports of increased family involvement, enabling patient advocacy.	
**Helpful in understanding loved one's illness**	93% agreed (71/76)		Improvement in knowledge regarding nutritional needs.	
**HADS - global score (0-42)**				Mean score<br>Control: 12.0<br>Intervention: 11.7<br>Change: -0.34 p=0.61<br>CI −1.67 - 0.99
**QOC - clinician-family communication (0-100)**				Mean score<br>Control: 62.7<br>Intervention: 69.1<br>Change: 6.39 p=0.001<br>CI 2.57 - 10.20
**PPPC - Patient Perception of Patient Centeredness (1-4)**				Mean score<br>Control: 1.8<br>Intervention: 1.7<br>Change: -0.15 p=0.006<br>CI −0.26 - −0.04
**Length of stay - Hospital**				Mean score<br>Control: 6.7<br>Intervention: 7.4<br>Change: 0.90 p=0.045<br>CI 0.81 - 1.00
**Length of stay - ICU**				Mean score<br>Control: 13.5<br>Intervention: 10.4<br>Change: 0.77 p<0.001<br>CI 0.69 - 0.87
**IES - PTSD (0-88)**				Mean score<br>Control: 20.3<br>Intervention: 21.2<br>Change: 0.90 p=0.49<br>CI −1.66 - 3.47

Supplementary Table 4: Discussion based family involvement interventions

Study	Almoosa et al. 2009	Hollman et al. 2018	Garrouste et al. 2016	Huang et al. 2018	Curtis et al. 2016	Shaw et al. 2014
Intervention / Measure	**CPR discussions**	**Health conversations**	**Interprofessional family conference**	**Primary physician involvement**	**Communication facilitator**	**Team training**
**Satisfaction with CPR Discussion**	Questions answered 84% (36/43) CI 70-91<br>Facilitated decision 77% (33/42) CI 64-88					
**Time to decision**	Days mean (sd)<br>Control 7.4 (3.2)<br>Intervention 6.6 (6.5)<br>Change -0.8 days<br>CI -1.48-3.08					
**Death**	Control 8% (3/39)<br>Intervention 37% (17/45)<br>Change 31%<br>RR 4.91 CI 1.55-15.51					
**Change to less aggressive preference**	Control 18% (8/39)<br>Intervention 25% (11/45)<br>RR: 1.19 CI 0.53-2.66					
**Inclusion in decision making**		Qualitative findings of greater family engagement with ICU nurses.		% completely satisfied<br>Control: 61.4%<br>Intervention: 75.9%<br>Change: 14.5% p=0.05<br>RR: 1.23 CI 1.03-1.49		

Study / Intervention / Measure	Almoosa et al. 2009	Hollman et al. 2018	Garrouste et al. 2016	Huang et al. 2018	Curtis et al. 2016	Shaw et al. 2014
	**CPR discussions**	**Health conversations**	**Interprofessional family conference**	**Primary physician involvement**	**Communication facilitator**	**Team training**
**HADS - anxiety**			At 90 days, Median [IQR]<br>Control: 8 [4.5-12]<br>Intervention: 4 [1-9]<br>Change: 4 p=0.01<br><br>% sign. Anxiety (>8)<br>Control: 52.3% (n=23)<br>Intervention: 33.3% (n=14)<br>Change: 19% p=0.08<br>RR: 0.95, CI 0.63-1.44			
**HADS - depression**			At 90 days, Median [IQR]<br>Control: 5.5 [1-11.5]<br>Intervention: 2 [0-6]<br>Change: -3.5 p=0.04<br><br>% sign. Depress. score > 8<br>Control: 38.6% (n=17)<br>Intervention: 23.8% (n=10)<br>Change: 14.8% p=0.14<br>RR: 0.61, CI 0.31-1.18			
**PDEQ**			Median [IQR]<br>Control: 14.5 [11-23]<br>Intervention: 13 [0-17]<br>Change: -1.5 p=0.17			
**IES-R**			At 90 days, Median [IQR]<br>Control: 24 [12.5-45]<br>Intervention: 21 [9-33]<br>Change: -3 p=0.24			
**FS-ICU (24) - ICU Care**				Mean (sd)<br>Control: 88.38 (14.5)<br>Intervention: 90.22 (12.68)<br>Change: 1.84 p=0.28<br>CI -2.42 - 6.09		
**FS-ICU (24) - Global score**				Mean (sd)<br>Control: 84.91 (12.17)<br>Intervention: 86.4 (11.76)<br>Change: 1.49 p=0.16<br>CI -2.14 - 5.12		
**FS-ICU (24) - Decision making**				Mean (sd)<br>Control: 80.07 (12.76)<br>Intervention: 81.06 (15.1)<br>Change: 0.99 p=0.16<br>CI -3.00 - 4.98		
**Control over patient care**				% completely satisfied<br>Control: 55.6%<br>Intervention: 73.6%<br>Change: 18% p=0.02<br>RR: 1.31 CI 1.07-1.61		
**GAD 7 - anxiety**					Mean score 3 months<br>Control: 3.0<br>Intervention: 2.3<br>Change: -0.7 p=0.50<br>CI: -2.91 - 1.42<br><br>Mean score 6 months<br>Control: 2.7<br>Intervention: 1.8	

Study / Intervention / Measure	Almoosa et al. 2009	Hollman et al. 2018	Garrouste et al. 2016	Huang et al. 2018	Curtis et al. 2016	Shaw et al. 2014
**Intervention**	**CPR discussions**	**Health conversations**	**Interprofessional family conference**	**Primary physician involvement**	**Communication facilitator**	**Team training**
					Change: -0.9 p=0.43<br>CI: -3.10 - 1.32	
**PHQ 9 - depression**					Mean score 3 months<br>Control: 4.9<br>Intervention: 3.1<br>Change: -1.8 p=0.09<br>CI: -3.89 - 0.31<br><br>Mean score 6 months<br>Control: 4.7<br>Intervention: 2.4<br>Change: -2.3 p=0.01<br>CI: -4.30 - -0.42	
**PCL - PTSD**					Mean score 3 months<br>Control: 31.6<br>Intervention: 29.8<br>Change: -1.7 p=0.47<br>CI: -6.65 - 3.12<br><br>Mean score 6 months<br>Control: 30.6<br>Intervention: 27.1<br>Change: -3.5 p=0.056<br>CI: -7.12 - 0.09	
**Staff confidence. All items improved, biggest change: confidence in encouraging family presence**						Mean score (SD)<br>Pre: 7.0 (1.7)<br>Post: 8.4 (1.1)<br>Change: 1.4<br>p<0.001<br>CI: 0.81-1.99
**FS ICU 24 - Frequency nurse communication**						Mean score<br>Pre: 79.2<br>Post: 87.18<br>Change: 7.98<br>p=0.04
**FS ICU 24 - Frequency doctor communication**						Mean score<br>Pre: 67.86<br>Post: 76.69<br>Change: 8.83<br>p=0.04
**FS ICU 24 - Honesty of information**						Mean score<br>Pre: 77.78<br>Post: 87.08<br>Change: 9.30<br>p=0.01
**FS-ICU (24) - Decision making**						Mean score<br>Pre: 77.47<br>Post: 83.32<br>Change: 5.85<br>p=0.05
**FS-ICU (24) - Global score**						Mean score<br>Pre: 83.21<br>Post: 85.69<br>Change: 2.48<br>p=0.32

Supplementary Table 5: Web-based support interventions

Study / Intervention / Measure	Dalal et al. 2015	Dykes et al. 2017	Ernecoff et al. 2016	Huffines et al. 2013
**Intervention**	**Patient centred toolkit**	**Patient engagement ICT**	**Tablet decision support**	**Support care algorithm**
**Usability**	Mean (sd) usability score<br>m 74 (16.7)			
**Satisfaction**	Satisfaction 72% (13/18)<br>Mean (sd) satisfaction score<br>4.06 (0.94)			

Study	Dalal et al. 2015	Dykes et al. 2017	Ernecoff et al. 2016	Huffines et al. 2013
**Measure** / **Intervention**	**Patient centred toolkit**	**Patient engagement ICT**	**Tablet decision support**	**Support care algorithm**
**HCAHPS**		% top score 9-10<br>Pre: 71.8%, n=53, CI 61.1-82.6<br>Post: 93.3%, n=58, CI 88.2-98.4<br>Change 21.5<br>RR 1.33 CI 1.10-1.55		
**FS-ICU (20)**		Mean score<br>Pre: 84.3, n=106, CI 81.3-87.3<br>Post: 90, n=156, CI 88.1-91.9<br>Change: 5.7, CI 2.31-9.09		
**Adverse events**		Pre: 59/1000 patient days<br>Post: 42/1000 patient days<br>Change 17, CI 6.95-27.05<br>Relative reduction 29%		
**Length of stay**		Mean (median) LoS [range]<br>Pre: 4.9 (2) [1-108], n=881<br>Post: 5.0 (2) [1-115, n=904<br>p=0.61		
**Share in decisions about care planning**			Qualitative perceptions of improving family engagement, communication and involvement in decision making.	Mean satisfaction (1-4)<br>Pre: 2.97 | Post: 3.41<br>Change: 0.44 p=0.07<br><br>% scoring excellent<br>Pre: 45% | Post: 68%<br>Change: 23% p=0.009<br>RR: 1.52 CI 1.03-2.24
**Support and encouragement given**				Mean satisfaction (1-4)<br>Pre: 3.38 | Post: 3.52<br>Change: 0.15 p=0.47<br><br>% scoring excellent<br>Pre: 60% | Post 75%<br>Change: 15% p=0.14<br>RR: 1.23 CI 0.91-1.65
**Nurses and doctors working together as a team**				Mean satisfaction (1-4)<br>Pre: 3.38 | Post: 3.73<br>Change: 0.35 p=0.04<br><br>% scoring excellent<br>Pre: 64% | Post: 83%<br>Change: 19% p=0.04<br>RR: 1.31 CI 1.00-1.71

Supplementary Table 6: Environmental unit changes & Participation in care interventions

Study	Choi et al 2013	Rippin et al. 2015	Prichard & Newcomb 2015
	**Environmental unit changes**		**Physical participation in care**
**Measure** / **Intervention**	**PFC Unit design**	**Family centred design**	**Hand massage**
**Family interaction with patient**	r=0.632 | p<0.01		
**Family interaction with staff**	r=0.552 | p<0.01	Qualitative observations of increased, family-initiated interactions.	
**HADS - anxiety**			Mean change in score<br>Control: -0.4<br>Intervention: -3.87<br>Change: -3.47 p=0.002<br>CI -5.5 - -1.4
**HADS - depression**			Mean change in score<br>Control: -0.3<br>Intervention: -2.5<br>Change: -2.2 p=0.10<br>CI -0.49 - 4.7

Xyrichis A, *et al. BMJ Open* 2021; 11:e042556. doi: 10.1136/bmjopen-2020-042556
