# Supplementary material for: Interventions to promote family member involvement in adult critical care settings: a systematic review
Source: BMJ Open. 2021 Apr 7;11(4):e042556. doi: 10.1136/bmjopen-2020-042556 (PMC8031009; doi:10.1136/bmjopen-2020-042556)
Supplement: Author's manuscript [file bmjopen-2020-042556.draft_revisions.pdf]

BMJ Open is committed to open peer review. As part of this commitment we make the peer review history of every article we publish publicly available.

When an article is published we post the peer reviewers' comments and the authors' responses online. We also post the versions of the paper that were used during peer review. These are the versions that the peer review comments apply to.

The versions of the paper that follow are the versions that were submitted during the peer review process. They are not the versions of record or the final published versions. They should not be cited or distributed as the published version of this manuscript.

BMJ Open is an open access journal and the full, final, typeset and author-corrected version of record of the manuscript is available on our site with no access controls, subscription charges or pay-per-view fees (http://bmjopen.bmj.com).

If you have any questions on BMJ Open's open peer review process please email info.bmjopen@bmj.com

Interventions to promote family member involvement in adult critical care settings: a systematic review.

Journal:	*BMJ Open*
Manuscript ID	bmjopen-2020-042556
Article Type:	Original research
Date Submitted by the Author:	09-Jul-2020

Complete List of Authors:	Xyrichis, Andreas; King's College London,<br>Fletcher, Simon; Kingston and St Georges University London, Health, Social Care and Education<br>Philippou, Julia; King's College London, Nursing & Midwifery<br>Brearley, Sally; King's College London, Florence Nightingale Faculty of Nursing and Midwifery<br>Terblanche, Marius; King's College London,<br>Rafferty, Anne Marie; King's College London, Florence Nightingale School of Nursing and Midwifery

Keywords:	Adult intensive & critical care < INTENSIVE & CRITICAL CARE, Quality in health care < HEALTH SERVICES ADMINISTRATION & MANAGEMENT, Health policy < HEALTH SERVICES ADMINISTRATION & MANAGEMENT

SCHOLARONE™
Manuscripts

I, the Submitting Author has the right to grant and does grant on behalf of all authors of the Work (as defined in the below author licence), an exclusive licence and/or a non-exclusive licence for contributions from authors who are: i) UK Crown employees; ii) where BMJ has agreed a CC-BY licence shall apply, and/or iii) in accordance with the terms applicable for US Federal Government officers or employees acting as part of their official duties; on a worldwide, perpetual, irrevocable, royalty-free basis to BMJ Publishing Group Ltd ("BMJ") its licensees and where the relevant Journal is co-owned by BMJ to the co-owners of the Journal, to publish the Work in this journal and any other BMJ products and to exploit all rights, as set out in our licence.

The Submitting Author accepts and understands that any supply made under these terms is made by BMJ to the Submitting Author unless you are acting as an employee on behalf of your employer or a postgraduate student of an affiliated institution which is paying any applicable article publishing charge ("APC") for Open Access articles. Where the Submitting Author wishes to make the Work available on an Open Access basis (and intends to pay the relevant APC), the terms of reuse of such Open Access shall be governed by a Creative Commons licence – details of these licences and which Creative Commons licence will apply to this Work are set out in our licence referred to above.

Other than as permitted in any relevant BMJ Author's Self Archiving Policies, I confirm this Work has not been accepted for publication elsewhere, is not being considered for publication elsewhere and does not duplicate material already published. I confirm all authors consent to publication of this Work and authorise the granting of this licence.

For peer review only

TITLE PAGE

Interventions to promote family member involvement in adult critical care settings: a systematic review.

Andreas Xyrichis (corresponding author): Kings College London, James Clerk Maxwell Building, Waterloo, London, andreas.xyrichis@kcl.ac.uk
Simon Fletcher: Kingston & St Georges University London, Hunter Wing 6th Floor, St Georges University, Tooting, London, simon.fletcher@sgul.kingston.ac.uk
Julia Philippou: Kings College London, James Clerk Maxwell Building, Waterloo, London, julia.philippou@kcl.ac.uk
Sally Brearley: Kingston & St Georges University London, Hunter Wing 6th Floor, St Georges University, Tooting, London, sally.brearley@icloud.com
Marius Terblanche: Kings College London, James Clerk Maxwell Building, Waterloo, London, marius.terblanche@kcl.ac.uk
Anne Marie Rafferty: Kings College London, James Clerk Maxwell Building, Waterloo, London, anne_marie.rafferty@kcl.ac.uk

Manuscript word count (excluding tables, references): 3651

For peer review only - http://bmjopen.bmj.com/site/about/guidelines.xhtml

ABSTRACT

Objective: To identify, appraise and synthesise evidence of interventions designed to promote family member involvement in adult critical care units; and to develop a working typology of interventions for use by health professionals and family members.

Design: Mixed-method systematic review.

Data sources: Bibliographic databases were searched without date restriction: MEDLINE, EMBASE and CINAHL; the Cochrane Central Register of Controlled Trials, Joanna Briggs and Cochrane Libraries. Back issues of leading critical care and patient experience journals were manually searched, as were the reference lists of included studies. All evaluation studies of relevant intervention activities were included; all research designs and outcome measures were eligible. Due to heterogeneity in interventions, designs and outcome measures the synthesis followed a narrative approach. Service users met with the research team termly.

Results: Out of 5,109 possible citations a total of 21 studies were included. The overall evidence base was assessed as moderate to weak, with only a few exceptions of high-quality qualitative and quantitative studies. Six main outcomes were identified, grouped under two categories: i) Involvement outcomes: Communication, Decision making, Satisfaction; and ii) Health outcomes: Family Trauma, Family Wellbeing, Patient outcomes. The findings from the qualitative studies were thematically analysed to identify features of the interventions that participants perceived to influence effectiveness. Synthesised into five overarching categories, these can serve as principles to inform the future design and development of more refined family member involvement interventions.

Conclusions: Future interventions should be developed with much closer family member input and designed by taking into account the key success features we identified. We call for future interventions to be multi-layered and allow for a greater or lesser level, and different kind, of involvement for family members, the decision of which should be informed by a baseline diagnostic of family members' needs, readiness and preparedness for involvement.

Review registration: PROSPERO 2018 CRD42018086325

Keywords: Critical care units, family involvement, systematic review, typology, service user

For peer review only

ARTICLE SUMMARY

Strengths and limitations of this study

We completed a comprehensive mixed-method systematic review of available evidence involving a Service Users & Carers group throughout the process, including 16 hours of in-depth group discussions.

The evidence base is heterogeneous in terms of study designs, outcome measures and types of interventions, hindering statistical synthesis through a meta-analysis.

We identify key weaknesses in family involvement intervention development to date, and present key principles to guide future initiatives.

We present a typology of interventions that can guide research and practice in this area, developed with input from service users and carers, which now requires empirical validation.

For peer review only

For peer review only - http://bmjopen.bmj.com/site/about/guidelines.xhtml

BACKGROUND

Family member involvement in healthcare is recognised by policymakers and the public as an invaluable aspect of healthcare provision. The move towards the more structured integration of family members is consistent with the call for greater patient-centred care, long invoked by a (2001) report from the Institute of Medicine[1]. Emphasising the potential benefits in actively involving family members in decision-making processes and care management was regarded as a key contributor to the reduction of the risk of medical error and a broader improvement in quality. The idea that a 'fully-engaged' public[2] should be encouraged has also been evident in United Kingdom contexts, however governmental scrutiny over the proceeding decade has revealed that this has yet to be comprehensively realised. Two strategic reports from NHS England[3,4] explored a practical move towards a healthcare system which is built around the patient, incorporating families representing a key means of achieving this.

For the purposes of the present review, family member involvement refers to the activities of different professionals to improve care by ensuring family members are involved in decision making, sharing of information, power and responsibility for patient needs and choices. This is especially relevant in the intensive care unit (ICU), where it can have profound consequences for care decisions, delivery and outcomes. This is partly because ICU patients are rarely in a position to communicate directly with clinicians and recollect their ICU experience, which means that the responsibility for this often lies with their family members. While this is a concern in healthcare systems worldwide, and especially in the UK, a recent mixed-methods study found there is still great variation in family satisfaction across ICUs in England, Wales and Northern Ireland[5].

Studies over the past decade have confirmed that family members can have a positive influence on a patients' care and recovery from ICU; but also that family members themselves can be affected even after discharge[6-11]. In particular, within the first few days after ICU admission, family members can show signs of anxiety, depression and stress; report difficulties in understanding the information clinicians try to communicate with them; and those who suffered a bereavement are at risk of generalized anxiety, panic attacks, depression, and post-traumatic stress syndrome (PTSD)[7]. For example, one study[6] surveyed family members of patients who have been in ICU to find that 90 days after discharge more than a third (34%) of them suffered from PTSD symptoms. In addition, they noted higher rates (48%) among those family members who indicated the information they were given was incomplete.

While there is a growing evidence base on patients, family members and clinicians' perceptions of involvement in ICU care, we are still missing a standardised, evidence-based approach to facilitate this process. Indeed, a recent scoping review[10] sought to investigate the extent and range of literature on this topic, finding evidence of a growth in papers with over 100 reports identified. However, they did not seek to assess the quality of the evidence nor did they examine the type of interventions available, and key features that foster

For peer review only - http://bmjopen.bmj.com/site/about/guidelines.xhtml

effectiveness. While this is an area of growing interest, we are still unclear about the range and quality of interventions available to promote family member involvement in ICU.

METHODS

Our review sought to answer the following question: What are the available interventions, and which are most effective, for including family members in the care processes and decisions made in adult critical care settings? (Review registration: PROSPERO 2018 CRD42018086325

Four objectives were set: a) undertake a comprehensive and systematic search of published and unpublished studies reporting on interventions that promote involvement in critical care; b) robustly assess the quality of empirical evidence for all included studies; c) generate a detailed description and synthesis of interventions and their associated outcomes; and d) classify interventions and outcomes in order to develop a typology of interventions, outlining key factors that support or impede involvement.

Study eligibility criteria

We included evaluation studies of any design, including experimental and quasi-experimental studies, as well as action research, case study and ethnographic designs. We considered reports of any kind of interventions as long as they were intended to promote the participation of family members in adult critical care. We included studies that reported on a mixture of relevant outcomes, including standard measures such as the Hospital Anxiety and Depression Score (HADS); and non-standard but important indicators such as family satisfaction.

Search for evidence

We searched MEDLINE, EMBASE, CINAHL and the Cochrane Central Register of Controlled Trials following a systematic approach (Table 1). We also performed searches for clinical studies through the WHO Trials Registry. We hand searched recent back issues of key critical care and patient experience e-journals, scanned reference lists of identified reviews and included articles; searched our personal libraries and consulted with experts in the field. GoogleScholar was also searched. Non-commercially published (Grey) literature was sought through the OpenGrey and the GreyLit Report databases. A standardised search strategy was developed with the input of an information specialist and applied to all databases, utilising both MeSH and freetext terms.

Data management, screening, extraction and quality assessment

Search results were imported into Covidence, the standard production platform for Cochrane Reviews. Two reviewers (AX, SF) independently screened all citations in duplicate against the inclusion criteria. Any disagreement was resolved through discussion or by involving a third reviewer (JP). Reasons for excluding papers read in full were recorded and a PRISMA flowchart

For peer review only

was completed. Standardised data extraction forms were used by two reviewers (SF, AX) to independently extract key information. Any disagreements found at this stage were also resolved through a consensus approach, or the involvement of a third reviewer (JP). We assessed each included paper for methodological rigour using an established rating approach utilised successfully in a number of previous mixed-method systematic reviews[12,13]. Quality assessment was undertaken independently by two reviewers (AX, SF) in duplicate. For each quality domain we described the procedures reported in the paper, using verbatim quotes as necessary.

Data synthesis

The overall approach to the synthesis followed a segregated methodologies approach[14]. Accordingly, a separate synthesis of quantitative and qualitative data was initially completed followed by a mixed-method synthesis. Quantitative studies were grouped depending on study design, and results summarised using descriptive statistics. Because heterogeneity precluded statistical combination through a meta-analysis, a narrative approach to the quantitative synthesis was pursued. Synthesis of qualitative data followed the Best-fit framework approach, involving identification and grouping of thematic categories around factors influencing effectiveness of family member involvement interventions[15]. The mixed-method synthesis utilised the quantitative data to inform on the measured effects, while the qualitative data informed on the perceived effects. In this way, the qualitative and quantitative data acted as complementary rather than as confirmatory of each other. A key element of the mixed-method synthesis was to classify interventions and outcomes in order to develop a typology of family involvement interventions in ICU that presents key factors that support or impede involvement. The typology is illustrated in a matrix-like format and includes interventions supported by either suggestive or firm evidence.

Patient and public involvement

A Service Users & Carers Group was established from the outset, the members of which were recruited through promotion in social media, the UK ICUSteps charity and the NIHR INVOLVE website. Consisting of eight ICU survivors and carers, the group was consulted through each step of the process and engaged in four strategically positioned meetings leading to over 16 hours of focussed and in-depth face-to-face discussions concerning the design, conduct, reporting and dissemination of the review.

RESULTS

Overview of included studies

Search results

Out of 5,109 possible citations a total of 21 studies were included (Figure 1). Published between 1989 and 2018, they were predominantly from North American contexts (n=17), and from a range of ICU settings including surgical, neuroscience, medical oncology and trauma

For peer review only - http://bmjopen.bmj.com/site/about/guidelines.xhtml

ICUs. One study came from a French setting, and others from Swedish, Spanish and Australian ICU settings.

Study designs

Most of the studies followed a quantitative approach (n=16), but we also included four qualitative and one mixed methods study (Table 2). Specifically, quantitative studies included randomised controlled trials, cluster-randomised and stepped-wedge trials, non-randomised before and after studies, and prospective observational multi-centre studies. Qualitative data from all studies came from semi-structured interviews.

Intervention types

Interventions were grouped based on their distinguishing characteristics (Table 3), and positioned within a continuum of low to high involvement: i) environmental unit changes (n=2)[16-17], ii) web-based support (n=4)[18-21], iii) discussion-based support (n=6)[22-27], iv) multicomponent support (n=4)[28-31], v) participation in rounds (n=3)[32-34], and vi) participation in physical care (n=1)[35]. Environmental unit change interventions consisted of complete structural redesign of an existing ICU with the specific objective being enhanced family member presence. Web based and electronic interventions were approaches which utilised information and communication technology to facilitate information sharing and asynchronous communication. Discussion-based interventions generally consisted of one off or repeated face-to-face conversations which took place in the unit between family members and healthcare professionals. Multi-component interventions comprised more than one technique to engage, educate and communicate with family members in the ICU. Family involvement in rounds interventions enabled the family members to attend and watch the daily rounds process, with opportunity to raise questions and clarify issues. Physical participation in care interventions consisted of physical touch between family members and patients being utilised for reciprocal benefit.

Quality of evidence

The overall evidence base was assessed to be moderate to weak, with only a few exceptions of high-quality qualitative and quantitative studies (Tables 4 and 5). The main weaknesses of the quantitative studies were related to inadequate randomisation and blinding, and lacking a control group for comparison purposes. The qualitative studies were in the main of a better quality, with key weaknesses being related to inadequate researcher reflexivity and theorisation of findings.

Quantitative results

Six main outcome categories were identified from the studies analysed for the review, grouped under two groups. Involvement outcomes: Communication, Decision making, Satisfaction; and Health outcomes: Family Trauma, Family Wellbeing, Patient outcomes. A summary of key results is provided below, with detailed reporting of outcome measures available in Tables 6 to 15.

For peer review only - http://bmjopen.bmj.com/site/about/guidelines.xhtml

Involvement outcomes

Communication: Improved communication was considered as an outcome in five studies[32,33,27,34,31] reporting on four measurement tools. Three studies[33,27,34] utilised the Family Satisfaction ICU (FS ICU 24) tool, specifically reporting on three dimensions: honesty of information, frequency of doctor communication, frequency of nurse communication. One study[31] reported on the Quality of Communication (QOC) tool and another used a self-developed questionnaire[32]. Mean improvements were found in all studies, although not all results reached statistical significance. The biggest effect was seen on the frequency of doctor communication, with one study showing a statistically significant improvement of 60% (RR: 1.60, CI 1.18 to 2.17, p=0.004)[33].

Decision making: Six of the included studies[22,25,21,33,27,34] looked at decision making as an outcome, through six measurement tools. The 'decision making' sub-scale of the FS ICU 24 revealed improvements in all three studies although only one[27] found a statistically significant result (MD: 5.85, p=0.05). Inclusion in decision making was also considered, as the percentage change in family members reporting complete satisfaction or giving the highest score, with one study[25] showing a statistically significant improvement of 23% (RR: 1.23, CI: 1.03 to 1.49, p=0.05). Studies also noted improvements with regard to 'control over patient care' (RR: 1.31, CI: 1.07 to 1.61, p=0.02)[25], 'support in decision making' (RR: 1.39, CI: 1.09 – 1.79)[21] and 'share in decisions about care planning' (23%, RR: 1.52, CI: 1.03 to 2.24, p=0.009)[21].

Satisfaction: Satisfaction was considered as an outcome in nine studies[28,18,19,25,21,33,27,34,31] using six different measures. The majority of studies (n=4) measured family satisfaction with care using the FS-ICU, while the remaining studies used self-developed tools. One of the studies[19] also used the HCAHPS (Hospital Consumer Assessment of Healthcare Providers and Systems) Survey. This is a national, standardized, publicly reported survey of patients' perspectives of hospital care in the US. Improvements on the FS ICU were found in all studies, ranging from 1.49 to 5.7, although only one[19] reached statistical significance (MD: 5.7, CI: 2.31 to 9.09, p<0.05). Statistically significant improvements were also noted on the HCAHPS (RR: 1.33, CI: 1.10 to 1.55, p<0.05)[19].

Health outcomes

Family wellbeing was considered through measurements for anxiety and depression.

Anxiety: Four studies considered anxiety as an outcome[23,35,26,31] using three different ways for measurement. Three studies[23,35,31] used the Hospital Anxiety and Depression Scale (HADS). The fourth study[26] used the Generalised Anxiety Disorder Assessment (GAD-7). All four studies identified a reduction in family members' anxiety scores post the intervention, with the effect ranging from -0.34 to -4. The minimal clinically important difference of HADS has been suggested to be between -1.5 and -2, therefore the reduction noted in these papers could be of clinical significance. However, only two of these studies found statistical significance[23,35].

For peer review only - http://bmjopen.bmj.com/site/about/guidelines.xhtml

Depression: Depression was an outcome reported by three studies[23,35,26] using two measures. Two studies[23,35] used the depression subscale of the Hospital Anxiety and Depression Scale (HADS) while another[26] used the Patient Health Questionnaire (PHQ-9). Both studies that used the HADS identified a reduction in depression among family members, although only one[23] reached statistical significance (p=0.04) with a median reduction of 3.5. Results on the PHQ-9 also showed a statistically significant mean reduction in scores at six months (MD: -2.3, CI: -4.30 to -0.42, p=0.01)[26].

Trauma: Three studies considered the impact of interventions on family member ICU-induced trauma using four different measurement tools[23,26,31]: Post-Traumatic Stress Disorder (PTSD) Checklist (PCL), Impact of Event Scale (IES) and Peritraumatic Dissociative Experience Questionnaire (PDEQ). All three studies identified a trend showing a beneficial effect of family involvement interventions on severity of trauma through reduced severity scores, ranging from a reduction of -3 to -7 points on the various scales, however none of these results actually reached statistical significance.

Patient outcomes: Three kinds of patient outcomes were reported in three studies: patient mortality[22,31] adverse events[19] and length of stay[19,31]. A statistically significant increase in mortality was reported in two studies; specifically one study found an actual percentage increase in the intervention group of 31% (RR: 4.91, CI: 1.55 to 15.51, p<0.05)[22] while the other of 7.5% (RR: 1.27, CI: 1.09 to 1.48, p=0.008)[31]. A relative reduction in adverse events per 1000 patient days of 29% was also reported[9] with an actual mean difference of -17 (CI: 6.95 to 27.05, p<0.05). Length of stay was examined in two studies with conflicting results: one reported a statistically significant reduction (MD: -0.77, CI: -0.69 to -0.87, p<0.001)[31] while another did not find any statistically significant difference[19].

Qualitative results

Features that influence effectiveness

The findings from the qualitative studies (n=5) were analysed to identify features of the interventions that participants perceived to influence effectiveness. These included accessibility, simplicity, supplementarity, contextualisation, interprofessionality, consistency, relationship and confidence development. Synthesised into five overarching categories, these can serve as principles to inform the future design and development of more refined family member involvement interventions in ICU: a) Practicality, b) Development, c) Interaction, d) Reflexivity and e) Bridging.

Practicality: Including accessibility, simplicity, long term application and repetition, effective family involvement interventions need to be readily available with clear instructions, which are as close to universally identifiable as possible. This enables the intervention to be used many times if necessary, benefiting family members over and beyond the specific ICU experience.

Development: Developing family members' contextual knowledge can be particularly valuable for effective intervention delivery in ICU. Insight is key to developing shared

For peer review only - http://bmjopen.bmj.com/site/about/guidelines.xhtml

understanding among all those engaged in the treatment process. Insight should go beyond an understanding of the patient's condition to encompass the diverse perspectives of healthcare professionals and the emotional strain which family members naturally undergo.

Interaction: Each intervention, either explicitly or implicitly, encourages verbal and physical interaction between clinicians and family members. However, interprofessional discussions among ICU clinicians, and specifically between medical and nursing staff, are equally important. Interventions that foster quality interaction at all levels are essential for the family to be coherently integrated into the structure of the ICU.

Reflexivity: While effective interventions bring people together, they should also benefit the individual family member, who has the opportunity for personal growth in the path towards meaningful family member integration. Specific examples of reflexivity in the featured interventions were identified in the confirmatory exchanges between family members and healthcare professionals, as family members were able to explore their own role within the ICU, the relationships which they had independently developed with their clinicians and how they could utilise this unique knowledge as a form of leverage in their communication with ICU professionals.

Bridging: Effective interventions should also provide key and otherwise unavailable links which encourage family member integration. The supplementary capacity which effective interventions offer is of particular value when consistent or predictable communication cannot be guaranteed. While information exchange is important, an acknowledgment of the many difficulties which family members face in the ICU will ultimately enhance integration and experience.

DISCUSSION

Utilising the learning gained from the current review, and the insights of our service users and carers group, we propose a typology of interventions (Figure 2) to be considered when promoting family member involvement in ICU. Presented in a matrix, we position types of interventions along a continuum of low to high involvement (environmental unit changes, web-based support, discussion-based support, multicomponent support, participation in rounds and participation in physical care) along with their key characteristics (type of ICU, family time commitment, professional input, cost, opportunities & challenges), level of evidence (low, moderate, high) and impact on key involvement and health outcomes (satisfaction, decision making, communication, family wellbeing, family trauma, and patient outcomes).

We expect our typology to introduce some much-needed clarity in this field of research, as well as inform the development of future interventions and research studies. Importantly, the typology can be used by individual ICUs to inform discussions among professionals, and with family members, about which kinds of family involvement interventions they may adopt and implement in their units.

For peer review only - http://bmjopen.bmj.com/site/about/guidelines.xhtml

The diverse nature of the family involvement interventions we identified, the heterogeneity of research designs found, and the varying methodological quality of available studies meant there was not a single intervention that stood out as the recommended way forward. Interventions can be of varying cost, require different kinds and levels of input and time commitment from clinicians and family members, and impact on different kinds of outcomes. Given the current state of the evidence, we discourage proposing a single intervention as the 'gold standard' and instead encourage use of our typology to inform decisions based on each ICU context, its needs and available resources.

In addition, by having an engaged service user and carer group work with us throughout the completion of this review, we were able to look beyond the evidence by exploring the experiences and views of service users about the different interventions identified. This process, which involved four strategically positioned meetings leading to over 16 hours of focussed and in-depth face-to-face discussions, led to us to identify a key, fundamental flaw of existing family involvement interventions and research: these treat family members as a homogeneous group of individuals, rather than as heterogenous units. Specifically, different family members have different involvement needs and preferences influenced by their previous experiences, knowledge, mental and physical health, and socioeconomic state. In addition, the notion of a family member extends beyond the 'next of kin' or 'personal consultee' to encompass extended family, carers and friends. The dynamics within family units are crucial to shaping the experience, benefit and satisfaction of family members with ICU care and their level of involvement.

CONCLUSION

We argue that future interventions should be developed with much closer family member input and designed by taking into account the key success features we identified (practicality, development, interaction, reflexivity and bridging) through this review. Importantly, we call for future interventions to be multi-layered to allow for a greater or lesser level, and different kind of, involvement for family members, the decision of which should be informed by a baseline diagnostic of family members' readiness and preparedness for involvement.

DECLARATIONS

Acknowledgement

We are grateful to our Expert Advisory Board and Service User & Carer Group for their invaluable insight and support throughout this review. We owe intellectual debt to the late Professor Scott Reeves who generously supervised this project until his untimely passing in May 2018.

Ethics approval and consent to participate – Not applicable.

Consent for publication – Not applicable.

For peer review only - http://bmjopen.bmj.com/site/about/guidelines.xhtml

Availability of data and material – The datasets generated and/or analysed during the current study are available from the corresponding author on reasonable request.

Competing interests – The authors declare that they have no competing interests.

Funding – This paper presents independent research funded by the National Institute for Health Research (NIHR) under its Research for Patient Benefit (RfPB) Programme (Grant Reference Number PB-PG-0416-20021). The views expressed are those of the authors and not necessarily those of the NHS, the NIHR or the Department of Health and Social Care.

Author contributions – AX was responsible for conceiving the review, developing the review question and methodology, leading the funding application and coordinating the project. SF and JP were responsible for developing and executing the search strategy. AX, SF and JP were responsible for reference screening, quality assessment, data extraction and management. AX and SF were responsible for the statistical inferences, qualitative synthesis and interpretation of the data. SF and AX led the development of the first draft of the manuscript. All authors gave editorial comments on the draft manuscript. AX is the guarantor of the review. All authors read and approved the final manuscript.

For peer review only

REFERENCES

1 Institute of Medicine. To err is human New York: National Academies Press; 2001.

2 Wanless D. Securing our Future Health: Taking a Long-Term View London: HM Treasury; 2002.

3 NHS England. Transforming Participation in Health and Care: Guidance to support commissioners. London; 2013.

4 NHS England. Five Year Forward View. London; 2015.

5 Wright S, Walmsley E, Harvey S, Robinson E, Ferrando-Vivas P, Harrison D, et al. Family-Reported Experiences Evaluation (FREE) study: a mixed-methods study to evaluate families' satisfaction with adult critical care services in the NHS. Health Services and Delivery Research. 2015; 3: p. 45.

6 Azoulay E, Pochard F, Kentish-Barnes N, Chevret S, Aboab J, Adrie C, et al. Risk of post-traumatic stress symptoms in family members of intensive care unit patients. American Journal of Respiratory and Critical Care Medicine. 2005;: p. 987-994.

7 Azoulay E, Chaize M, Kentish-Barnes N. Involvement of ICU families in decisions: fine-tuning the partnership. Annals of Intensive Care. 2014; 4: p. 1.

8 Bench S, Cornish J, Xyrichis A. A qualitative exploration of Intensive Care discharge summaries for General Practice staff. British Journal of General Practice. 2016; 66(653): p. e904-e912.

9 Davidson JE, Powers K, Hedayat KM, Tieszen M, Kon AA, Shepard E, et al. Clinical practice guidelines for support of the family in the patient-centered intensive care unit: American College of Critical Care Medicine Task Force 2004–2005. Critical Care Medicine. 2007; 35(2): p. 605-622.

10 Olding M, McMillan SE, Reeves S, Schmitt MH, Puntillo K, & Kitto S. Patient and family involvement in adult critical and intensive care settings: a scoping review. Health Expectations. 2015; 19: p. 1183-1202.

11 Liput SA, Kane-Gill SL, Seybert AL, Smithburger PL. A Review of the Perceptions of Healthcare Providers and Family Members Toward Family Involvement in Active Adult Patient Care in the ICU. Critical Care Medicine. 2016; 44(6): p. 1191-1197.

12 Pauzé E, Reeves S. Examining the effects of interprofessional education on mental health providers: Findings from an updated systematic review. Journal of Mental Health. 2010; 19(3): p. 258-271.

13 Reeves S, Fletcher S, Barr H, Birch I, Boet S, Davies N, et al. A BEME systematic review of the effects of interprofessional education: BEME Guide No. 39. Medical Teacher. 2016; 38(7): p. 656-668.

14 Dufour É, Duhoux A, Bolduc J. Measured and perceived effects of audit and feedback on nursing performance: a mixed methods systematic review protocol. Systematic Reviews. 2019; 8(1): p. 38.

15 Carroll C, Booth A, Leaviss J, Rick J. “Best fit” framework synthesis: refining the method. Medical Research Methodology. 2013; 13(1): p. 37.

16 Choi YS, Bosch SJ. Environmental affordances: Designing for family presence and involvement in patient care. Health Environments Research & Design Journal. 2013; 6(4): p. 53-75.

17 Rippin AS, Zimring C, Samuels O, Denham ME. Finding a Middle Ground: Exploring the Impact of Patient-and Family-Centered Design on Nurse–Family Interactions in the Neuro ICU. Health Environments Research & Design Journal. 2015; 9(1): p. 80-98.

18 Dalal AK, Dykes PC, Collins S, Lehmann LS, Ohashi K, Rozenblum R, et al. A web-based, patient-centered toolkit to engage patients and caregivers in the acute care setting: a preliminary evaluation. Journal of the American Medical Informatics Association. 2015; 23(1): p. 80-87.

19 Dykes PC, Rozenblum R, Dalal A, Massaro A, Chang F, Clements M, et al. Prospective evaluation of a multifaceted intervention to improve outcomes in intensive care: the Promoting Respect and Ongoing Safety Through Patient Engagement Communication and Technology Study. Critical Care Medicine. 2017; 45(8): p. e806-e813.

20 Ernecoff NC, Witteman HO, Chon K, Buddadhumaruk P, Chiarchiaro J, Shotsberger KJ, et al. Key stakeholders' perceptions of the acceptability and usefulness of a tablet-based tool to improve communication and shared decision making in ICUs. Journal of Critical Care. 2016; 33: p. 19-25.

21 Huffines M, Johnson KL, Naranjo LLS, Lissauer ME, Fishel MAM, Howes SMA, et al. Improving family satisfaction and participation in decision making in an intensive care unit. Critical Care Nurse. 2013; 33(5): p. 56-69.

22 Almoosa KF, Goldenhar LM, Panos RJ. Characteristics of discussions on cardiopulmonary resuscitation between physicians and surrogates of critically ill patients. Journal of Critical Care. 2009; 24(2): p. 280-287.

For peer review only

23 Garrouste-Orgeas M, Max A, Lerin T, Grégoire C, Ruckly S, Kloeckner M, et al. Impact of proactive nurse participation in ICU family conferences: a mixed-method study. Critical Care Medicine. 2016; 44(6): p. 1116-1128.

24 Hollman Frisman G, Wåhlin I, Orvelius L, Ågren S. Health-promoting conversations - a novel approach to families experiencing critical illness in the ICU environment. Journal of Clinical Nursing. 2018; 27(3): p. 631-639.

25 Huang KB, Weber U, Johnson J, Anderson N, Knies AK, Nhundu B, et al. Primary Care Physician Involvement in Shared Decision Making for Critically Ill Patients and Family Satisfaction with Care. The Journal of the American Board of Family Medicine. 2018; 31(1): p. 64-72.

26 Curtis JR, Treece PD, Nielsen EL, Gold J, Ciechanowski PS, Shannon SE, et al. Randomized trial of communication facilitators to reduce family distress and intensity of end-of-life care. American Journal of Respiratory and Critical Care Medicine. 2016; 193(2): p. 154-162.

27 Shaw DJ, Davidson JE, Smilde RI, Sondoozi T, Agan D. Multidisciplinary team training to enhance family communication in the ICU. Critical Care Medicine. 2014; 42(2): p. 265-271.

28 Cray L. A collaborative project: initiating a family intervention program in a medical intensive care unit. Focus on Critical Care. 1989; 16(3): p. 213-218.

29 Davidson JE, Daly BJ, Agan D, Brady NR, Higgins PA. Facilitated sensemaking: a feasibility study for the provision of a family support program in the intensive care unit. Critical Care Nursing Quarterly. 2010; 33(2): p. 177-189.

30 Marshall AP, Wake E, Weisbrodt L, Dhaliwal R, Spencer A, Heyland DK. A multi-faceted, family-centred nutrition intervention to optimise nutrition intake of critically ill patients: The OPTICS feasibility study. Australian Critical Care. 2016; 29(2): p. 68-76.

31 White DB, Angus DC, Shields AM, Buddadhumaruk P, Pidro C, Paner C, et al. A Randomized Trial of a Family-Support Intervention in Intensive Care Units. New England Journal of Medicine. 2018; 378(25): p. 2365-2375.

32 Allen SR, Pascual J, Martin N, Reilly P, Luckianow G, Datner E, et al. A novel method of optimizing patient-and family-centered care in the ICU. Journal of Trauma and Acute Care Surgery. 2017; 82(3): p. 582-586.

33 Jacobowski NL, Girard TD, Mulder JA, Ely EW. Communication in critical care: family rounds in the intensive care unit. American Journal of Critical Care. 2010; 19(5): p. 421-430.

34 Weber U, Johnson J, Anderson N, Knies AK, Nhundu B, Bautista C, et al. Dedicated Afternoon Rounds for ICU Patients' Families and Family Satisfaction With Care. Critical Care Medicine. 2018; 46(4): p. 602-611.

35 Prichard C, Newcomb P. Benefit to family members of delivering hand massage with essential oils to critically ill patients. American Journal of Critical Care. 2015; 24(5): p. 446-449.

For peer review only - http://bmjopen.bmj.com/site/about/guidelines.xhtml

36 Martínez MD, Morilla FR, Del Pino ÁR, Almenara MI, Bannik JT, Caballero LJ, Macías GC, Bernet LA, González CA. Implicación familiar en los cuidados del paciente crítico. Enfermería Intensiva. 2003; 14(3): p. 96-108.

For peer review only

APPENDIX

Table 1: Database search string

1. exp Critical Illness/
2. exp Critical Care/
3. exp Intensive Care Units/
4. critical care.mp.
5. critical illness*.mp.
6. intensive care.mp.
7. ICU.mp.
8. ITU.mp.
9. OR/1-8
10. exp Patient Participation/
11. patient involve*.mp.
12. patient engage*.mp.
13. shared decision making.mp.
14. patient participat*.mp.
15. service user involve*.mp.
16. family involve*.mp.
17. family participat*.mp.
18. family engage*.mp.
19. caregiver involve*.mp.
20. caregiver engage*.mp.
21. caregiver participat*.mp.
22. relative involve*.mp.
23. relative engage*.mp.
24. relative participat*.mp.
25. OR/11-24
26. 9 AND 25

For peer review only

Table 2: Included studies

Author	Year	Country and setting	Design	Research approach	Intervention
Allen et al	2017	USA – Surgical ICU	Non-randomised, before and after study	Quantitative	Engaging family members on rounds
Almoosa et al	2009	USA – Medical ICU	Prospective 2-centre observational study	Quantitative	CPR Discussions
Choi and Bosch	2013	USA – Neuro ICU and Trauma ICU	Comparative study	Quantitative	Patient and family centred unit design
Cray	1989	USA – Medical ICU	Post hoc evaluation	Quantitative	Family intervention programme
Dalal et al	2015	USA – Medical ICU (Oncology Unit)	Post hoc evaluation	Quantitative	Patient centred toolkit
Davidson et al	2010	USA – Mixed ICU	Feasibility study	Quantitative	Family Support Programme, based on Facilitated Sensemaking
Dykes et al	2017	USA – Medical ICU	Prospective pre/post study	Quantitative	Patient Engagement Communication and Technology (PROSPECT)
Ernecoff et al	2016	USA – Medical ICU	Qualitative interview study	Qualitative	Tablet-based support tool
Garrouste-Orgeas et al	2016	France – Surgical ICU	Randomised controlled trial with qualitative component	Mixed methods	Proactive participation of a nurse in family conferences
Hollman Frisman et al	2018	Sweden – ICU	Qualitative study	Qualitative	Health promoting conversations
Huang et al	2018	USA – Neuroscience ICU	Prospective, observational single centre design	Quantitative	Primary care physician involvement in decision-making in the ICU
Huffines et al	2013	USA – Surgical ICU	Pre-post design	Quantitative	SICU Family supportive care algorithm
Marshall et al	2016	Australia – ICU	Prospective cohort study	Qualitative	Multi-faceted family-centred nutrition intervention
Jacobowski et al	2010	USA – Medical ICU	Before and after study	Quantitative	Family rounds
Prichard and Newcomb	2015	USA – Trauma intensive care unit	Quasi experimental pilot study	Quantitative	Hand massage
Randall-Curtis et al	2016	USA – ICU	Randomised controlled trial	Quantitative	Communication facilitator
Rippin et al	2015	USA – Neuroscience ICU	Comparative study	Qualitative	Family centred unit design
Shaw et al	2014	USA – ICU	Pre/post design	Quantitative	Multidisciplinary team training to enhance family communication in the ICU
Weber et al	2018	USA – Neuroscience ICU	Pre/post implementation study	Quantitative	Family rounds
White et al	2018	USA - 2 Neuro ICU, 2 Mixed ICU, 1 Medical ICU	Multicentre, stepped-wedge, cluster-randomized	Quantitative	Multicomponent family-support intervention

For peer review only - http://bmjopen.bmj.com/site/about/guidelines.xhtml

Table 3: Intervention characteristics

Author	Activity Type	Intervention	Purpose	Procedure	Participants	Beneficiary	Outcomes
Allen et al	Rounds based	Family member involvement in rounds	Engaging and integrating ICU patients' family members and surrogates on daily rounds	On admission to the ICU, a family member was invited to participate on daily rounds with the critical care team.	Family members, nurses, physicians	Family members, nurses, physicians	• significantly increased family member knowledge<br>• strengthened relationship between family members and doctors<br>• nurses reported: greater enjoyment<br>• improved communication<br>• reduction in workload<br>• increased support for patient and family centered care.<br>• increased physician satisfaction post intervention
Almoosa et al	Discussion based	CPR Discussions	The implementation of structured, informed CPR specific conversations with family members and surrogates from relevant physicians	Physician initiated CPR conversation about: chest compressions, electrical cardioversion, mechanical ventilation	Physicians, residents, fellows or family	Family members	• satisfaction with CPR discussions was higher after the intervention
Choi and Bosch	Unit design	Patient and family centred unit design	Unit redesign undertaken to enable physical space to more broadly facilitate family interaction with patient and family interaction with staff	The intervention unit included larger spaces and more comfortable accommodations for visitors	Family members	Family members and patients	• increased family interactions with patient<br>• increased family interactions with staff
Cray et al	Multi-component	Family intervention programme	Multi-component intervention to enhance family involvement in and understanding of their loved one's condition in ICU	Intervention components included: Family conference, Telephone conversation, Visit by nurse, Follow up visit to the intermediary care unit	Family members, Clinical nurse specialists	Family members	• strong agreement that the intervention helped family members to understand their loved one's illness and benefit other families.<br>• high satisfaction with intervention components.

For peer review only - http://bmjopen.bmj.com/site/about/guidelines.xhtml

Author	Activity Type	Intervention	Purpose	Procedure	Participants	Beneficiary	Outcomes
Dalal et al	Web based/ electronic	Patient centred toolkit (PCTK)	The PCTK was a suite of web-based patient-facing and provider-facing tools designed to facilitate collaborative decision making by providing access to tailored educational content and facilitating patient-provider communication	The PCTK was accessible by patients, caregivers, and providers from any web-enabled device connected to the hospital's secure intranet.	Patients and caregivers	Patients and family members	• the majority of patients and caregivers surveyed were satisfied or extremely satisfied with the intervention
Davidson et al	Multi-component	Family Support Programme, based on Facilitated Sensemaking	A systems-based patient-centered care and engagement program	The intervention consisted of 2 main components: personalized instruction and provision of family visiting kits.	Family members, Clinical nurse specialists	Family members	• high rate of instrument reliability<br>• a breakdown of the top ten needs of family members<br>• a ranking of interventions' helpfulness<br>• identification of any additional needs
Dykes et al	Web based/ electronic	Promoting Respect and Ongoing Safety through Patient Engagement Communication and Technology (PROSPECT).	A systems-based patient-centered care and engagement program designed to enhance patient and care partner experience in the ICU	60 minute training session to facilitate patient-centred care in healthcare professionals,<br>A web based – toolkit to apply the training,<br>Delivery of the Patient SatisfActive Model from nurses to patients in ICU	Nurses and patients	Nurses and patients	• relative reduction in the rate of adverse effects<br>• improvements in patient satisfaction<br>• improvements in care partner satisfaction

For peer review only - http://bmjopen.bmj.com/site/about/guidelines.xhtml

Author	Activity Type	Intervention	Purpose	Procedure	Participants	Beneficiary	Outcomes
Ernecoff et al	Web based/ electronic	Tablet-based and video-driven communication and decision support tool	A tool was conceptualised to: 1) prepare the family for conversations with clinicians, 2)give clinicians tailored information about the family and patient in advance of the family meeting, 3) promote a personalized relationship between clinician and family, and 4) provide general decision support to surrogates.	The sections of the tool for surrogates were: 1) orienting surrogates to the ICU, 2) explaining principles of surrogate decision making 3) providing a question prompt list and opportunity to write down questions, 4) a values clarification exercise 5) education about treatment pathways (e.g., life prolonging treatment, comfort-focused treatment; and a time-limited trial of ICU care);6) eliciting surrogates prognostic information, and 7) providing psycho-social resources	Family members (surrogates)	Family members	• enhancing and supplementing communication between surrogates and the clinical team<br>• leveraging surrogates' downtime before and between clinician-family meetings<br>• helping surrogates to consider the patient's values and treatment options<br>• allowing for repetition and review of information
Garrouste-Orgeas et al	Discussion based	Proactive participation of a nurse in family conferences	Integration of nursing staff in family conferences focussed around: Naming emotions, expressing Understanding, showing Respect, articulating Support for the patient, and Exploring the family’s emotional state	All family members who wished to attend were escorted to the room. Briefly, an open question was asked first to encourage the family members to express themselves. A substantial proportion of the time was devoted to listening to the family, and the professionals used simple words to enhance comprehension. At the end of the conference, the family was allowed enough time to ask all the questions they had.	Physicians, nurses, family members	Family members	• family members reported that the conferences allowed them to receive and assimilate information<br>• be listened to regarding both their positive and negative feelings<br>• receive compassion and respect

For peer review only - http://bmjopen.bmj.com/site/about/guidelines.xhtml

Author	Activity Type	Intervention	Purpose	Procedure	Participants	Beneficiary	Outcomes
Hollman Frisman et al	Discussion based	(Nurse led) Health promoting conversations with families	The aim of the conversations was to create a context for change related to the families' problems and resources.	Discussion of the aim of the conversation series and the families' and nurses' expectations about the conversations and each other's roles. The three conversation sessions focused on topics that the families considered important, and the dialogue and questioning intended to identify resources within and outside the family. At the end of each conversation, the nurse offered a short reflection on how the family members had experienced the session. A closing letter was sent to the family 2–3 weeks after the last conversation to summarise further possibilities for reflection.	Nurses and family members	Family members	• health promoting conversations led to increasing emotional openness<br>• enhanced consciousness regarding illness<br>• greater family member satisfaction<br>• a valuable sense of confirmation<br>• general promotion of family wellbeing
Huang et al	Discussion based	Primary Care Physician involvement in decision-making in the ICU	The study evaluated survey results which measured family member satisfaction with general ICU care and shared decision making between Primary Care Physicians and family members	Examined involvement of the patient's Primary Care Physician and shared decision making.	Primary care physicians	Family members	• a higher proportion reporting PCP involvement felt completely satisfied with their inclusion in the ICU decision making process

For peer review only

Author	Activity Type	Intervention	Purpose	Procedure	Participants	Beneficiary	Outcomes
Huffines et al	Web based/ electronic	SICU Family Supportive Care Algorithm	The goal of the interventions in the 24-hour bundle is to inform families about the importance of their participation in decision making and to inform them of the resources available to help them participate in decision making.	Within 24 hours of admission, a member of the intensivist team meets with the family. Also within the first 24 hours after a patient's SICU admission, the patient's family is encouraged to watch an on-demand 10-minute video.<br>72 hours:<br>Reaffirm and ensure family is supported. Encouraged to participate in decision making in rounds.<br>96 hours:<br>Implement a family meeting with the multidisciplinary team.	Bedside nurses	Family members	• mixed to positive outcomes when measuring family satisfaction<br>• staff teamwork<br>• participation in decision making<br>• frequency of support
Marshall et al	Multi-component	Multi-faceted, family-centred nutrition intervention	To assist families in discussing nutritional goals with health professionals	Consisted of:<br>Family interviews,<br>Focussed education sessions, and Nutrition diaries	Dieticians, Family Nurses	Families and patients	• perceptions around how nutrition education can be improved<br>• the diversity of experience relating to the provision of in-hospital nutrition therapy<br>• continuity of existing family member involvement approaches<br>• the importance of families as advocates
Jacobowski et al	Rounds based	Family rounds	To enhance communication between medical and nursing staff and the families of ICU patients	The attending physician provided a summary for the family using understandable, lay language and the family was offered an opportunity to ask questions of the team	Physicians, nurses, family members	Physicians, nurses, family members	• communication regarding condition improved significantly as did decision making support.<br>• there was a decline in the number of family members who thought that they had sufficient time to address their questions and concerns.

For peer review only - http://bmjopen.bmj.com/site/about/guidelines.xhtml

Author	Activity Type	Intervention	Purpose	Procedure	Participants	Beneficiary	Outcomes
Prichard and Newcomb	Physical	Hand massage	To provide physical relief to patients in the ICU through hand massage	Participants were taught to administer hand massage in compliance with the M Technique, a registered method of simple, structured touch that has been used on critically ill patients with positive effects. For this study, the technique was used on hands. Participants applied the intervention twice daily for 5 minutes per session for 3 consecutive days	Family members, patients	Family members, patients	• anxiety was greatly reduced in the treatment group
Randall-Curtis et al	Discussion based	Communication-facilitator	To understand the family's concerns, needs, and communication characteristics	Consisted of facilitated: Interviews, Meetings, Communication and emotional support; as well as Facilitator participation in family conferences and 24 hour follow up	Nurse or social worker trained to improve communication between the ICU team and the family by acting as a communication facilitator or navigator	Family members	• adjusted depression scores were lowered alongside ICU costs
Rippin et al	Unit design	Family Centred Unit design	Unit designed to prioritise and engage the family in the ICU context	The FCU physically integrated family into the fabric of the unit. Nurses worked between centralized nursing stations and decentralized alcoves just outside patient rooms for improved monitoring and safety. Rooms were larger (245 sq ft) with more space around the bedside.	Nurses and family members	Family members	• reduction in problematic bedside co-presence (or overcrowding)<br>• a continuity of pre intervention distribution patterns in which nurse and family remained clustered in their respective 'domains'<br>• nurse perspectives which acknowledged the unpredictability and complexity of the ICU and the subsequent mixed results which this newly implemented unit design encouraged

For peer review only - http://bmjopen.bmj.com/site/about/guidelines.xhtml

Author	Activity Type	Intervention	Purpose	Procedure	Participants	Beneficiary	Outcomes
Shaw et al	Discussion based	Multidisciplinary team training to enhance family communication in the ICU	The training material was selected to improve communication. The training was also designed to foster team building and improve collaborative relationships among clinicians from multiple disciplines.	Training included how to conduct and participate in patient/family conferences, addressing goals of care and giving critical information in the intensive care setting so that all caregivers might speak to patients and their families with a common voice. The training was designed to address known drivers of family satisfaction, as well as to address each of the 21 items being measured in the staff confidence survey.	ICU team members	MDT team members and family members	• staff confidence and family satisfaction were shown to significantly improve
Weber et al	Rounds based	Family Rounds	To improve family satisfaction in ICU experience by integrating them into rounds processes	After each session, the nursing leader recorded (1) whether family rounds occurred (2), how many families participated, and (3) how many patients the NICU team had rounded on that morning	Nursing staff	Family members	• family reported improved satisfaction with decision making, frequency of communication, receiving emotional support and with coordination of care<br>• percentage improvements in satisfaction scores were not large enough to reach statistical significance
White et al	Multi-component	Multicomponent family-support intervention	Collaborative intervention between nursing staff and family members designed to support caregivers in the ICU	Nurses received advanced communication training.<br>A family support pathway was instituted. Intensive support for implementation was provided to each ICU by a quality-improvement specialist to incorporate the family-support pathway into clinicians' workflow.	Nursing staff, ICU team, family members	Family members and nursing staff	• significant improvements were noted with family satisfaction regarding:<br>• the quality of communication with clinicians, and<br>• perceptions of patient centeredness<br>• the intervention led to an average reduction in the length of stay in the ICU of 3 days

For peer review only - http://bmjopen.bmj.com/site/about/guidelines.xhtml

Table 4: Quality assessment - Quantitative studies

Study Id	Aim of Evaluation	Research Design	Sample size	Power calculation	Number of groups	Method of allocation	Allocation concealment	Blinding	Loss to follow up	Data collection method	Significance measures	Data analysis method	Reported biases or confounders	Relevance	Ethics	Strength of design	Strength of numbers	Quality of information	Quality of study	Overall weighting
Choi 2013	+	-	+	-	+	-	-	-	-	-	+	+	+	+	-	?	?	?	?	Low
Dalal 2016	-	-	+	-	-	-	-	-	-	-	+	+	+	+	+	?	?	?	?	Very low
Marshall 2016	+	+	+	-	?	-	-	-	-	+	+	+	+	+	+	+	+	?	+	Moderate
Allen 2017	+	-	+	-	-	-	-	-	-	-	+	+	+	+	+	?	?	?	?	Low
Dykes 2017	+	-	+	+	-	-	-	-	-	+	+	+	+	+	+	?	?	?	?	Low
Rodriguez Martinez 2003	+	+	+	?	+	-	-	?	?	+	+	+	+	+	-	?	+	?	?	Moderate
Huang 2018	+	+	+	+	+	-	-	-	-	+	+	+	+	+	+	-	+	+	+	Moderate
Randall Curtis 2016	+	+	+	+	+	+	+	?	-	+	+	+	+	+	+	+	+	+	+	Very high
Shaw 2014	+	?	+	+	+	?	?	?	-	+	+	+	+	+	+	?	+	+	?	High
Huffines 2013	+	+	+	-	+	-	-	-	+	+	+	+	+	+	?	?	?	?	?	Moderate
Garrouste-Orgeas 2016	+	+	+	+	+	+	+	-	+	+	+	+	+	+	+	+	+	+	+	Very high
NL 2010	+	?	+	?	+	?	?	?	?	+	+	+	+	+	+	?	+	?	?	Moderate
JE 2010	+	+	+	-	+	-	-	-	+	+	+	+	+	+	+	?	?	?	?	Moderate
Weber 2018	+	+	+	+	+	?	?	?	?	+	+	+	+	+	+	?	?	?	?	High
KF 2009	+	+	+	+	+	-	-	-	+	+	+	+	+	+	+	?	?	?	?	Moderate
Prichard 2015	+	?	+	?	+	-	?	?	+	+	+	+	+	+	+	?	+	+	?	High
Cray 1989	+	-	?	?	?	?	?	?	?	?	+	+	-	+	?	-	+	?	-	Low
White 2018	+	+	+	+	+	+	+	?	+	+	+	+	+	+	+	+	+	+	+	Very high

Table 5: Quality assessment - Qualitative studies

Study Id	Aim of Evaluation	Sampling	Data collection	Data analysis	Research relations	Findings	Transferability	Relevance & Usefulness	Ethics	Quality of information	Quality of study	Overall weighting
Marshall 2016	+	+	+	+	-	+	?	+	+	?	?	Moderate
Ernecoff 2016	+	+	+	+	-	+	+	+	+	+	+	High
Rippin 2015	+	?	+	+	?	+	+	+	-	?	?	Low
Garrouste-Orgeas 2016	+	+	+	+	-	+	+	+	+	+	+	High
Frisman 2018	+	+	+	+	-	+	+	+	+	+	+	High

For peer review only - http://bmjopen.bmj.com/site/about/guidelines.xhtml

Table 6: Anxiety outcome measures

Measure \ Study / Intervention	Garouste et al. 2016<br>IP family conference	Prichard & Newcomb 2015<br>Hand massage	White et al. 2018<br>Multi family support	Curtis et al. 2016<br>Communication facilitator
**HADS - Anxiety**	90 days, Median [IQR]<br>Control: 8 [4.5-12]<br>Intervention: 4 [1-9]<br>**Change: -4 p=0.01***<br><br>% w\ Anxiety score > 8<br>Control: 52.3% (n=23)<br>Intervention: 33.3% (n=14)<br>**Change: 19% p=0.08**<br>**RR: 0.95, CI 0.63-1.44**	Mean change in score<br>Control: -0.4<br>Intervention: -3.87<br>**Change: -3.47 p=0.002***<br>**CI -5.5 - -1.4**		
**HADS – Global score**			Mean score<br>Control: 12.0<br>Intervention: 11.7<br>**Change: -0.34 p=0.61**<br>**CI –1.67 – 0.99**	
**GAD 7 - Anxiety**				Mean score 3 months<br>Control: 3.0<br>Intervention: 2.3<br>**Change: -0.7 p=0.50**<br>**CI: -2.91 - 1.42**<br><br>Mean score 6 months<br>Control: 2.7<br>Intervention: 1.8<br>**Change: -0.9 p=0.43**<br>**CI: -3.10 - 1.32**
**Overall metric for comparison**	**MD: -4**	**MD: -3.47**	**MD: -0.34**	**MD: -0.7**

Table 7: Communication outcome measures

Measure \ Study / Intervention	Jacobowski et al. 2010<br>Family rounds	Weber et al. 2018<br>Family rounds	Shaw et al. 2014<br>Team training	White et al. 2018<br>Multi family support	Allen et al. 2017<br>Family on rounds
**FS ICU 24 – Frequency of nurse communication**	% highest score<br>Pre: 64%<br>Post: 57%<br>**Change: -7% p=0.30**<br>**RR: 0.89 CI: 0.70-1.12**	% top scores<br>Control: 62%<br>Intervention: 78%<br>**Change: 16% p>0.05**<br>**RR: 1.24 CI 0.98-1.58**	Mean score<br>Pre: 79.2<br>Post: 87.18<br>**Change: 7.98 p=0.04***		
**FS ICU 24 – Frequency of doctor communication**	% highest score<br>Pre: 38%<br>Post: 60%<br>**Change: 22% p=0.004***<br>**RR: 1.60 CI 1.18-2.17**	% top scores<br>Control: 43%<br>Intervention: 56%<br>**Change: 13% p>0.05**<br>**RR: 1.31 CI 0.90-1.89**	Mean score<br>Pre: 67.86<br>Post: 76.69<br>**Change: 8.83 p=0.04***		
**FS ICU 24 – Honesty of information**			Mean score<br>Pre: 77.78<br>Post: 87.08<br>**Change: 9.30 p=0.01***		

For peer review only - http://bmjopen.bmj.com/site/about/guidelines.xhtml

Study / Intervention / Measure	Jacobowski et al. 2010	Weber et al. 2018	Shaw et al. 2014	White et al. 2018	Allen et al. 2017
Intervention	Family rounds	Family rounds	Team training	Multi family support	Family on rounds
**Quality of Communication (QOC)**				Mean score<br>Control: 62.7<br>Intervention: 69.1<br>**Change: 6.39**<br>**p=0.001***<br>**CI 2.57 – 10.20**	
**Communication improvement**					Pre: n=49 (100%)<br>Post: n=47 (100%)<br>**Change 0%**<br>**p=0.68**<br>**RR: 1, CI: 0-0**
**Overall metric for comparison**	**RR: 1.60**	**RR: 1.31**	**MD: 8.83**	**MD: 6.39**	**RR: 1**

Table 8: Decision making outcome measures

Study / Intervention / Measure	Huang et al. 2018	Shaw et al. 2014	Weber et al. 2018	Jacobowski et al. 2010	Huffines et al. 2013	Almoosa et al. 2009
Intervention	Primary physician involvement	Team training	Family rounds	Family rounds	Support care algorithm	CPR discussions
**FS ICU 24 – Decision making**	Mean (sd)<br>Control: 80.07 (12.76)<br>Intervention: 81.06 (15.1)<br>**Change: 0.99 p=0.16**<br>**CI -3.00 – 4.98**	Mean score<br>Pre: 77.47<br>Post: 83.32<br>**Change: 5.85**<br>**p=0.05***	Mean score (SD)<br>Control: 85.1 (16.3)<br>Intervention: 88.6 (14.6)<br>**Change: 3.5**<br>**p=0.20**<br>**CI: -1.98-8.98**			
**Inclusion in decision making**	% completely satisfied<br>Control: 61.4%<br>Intervention: 75.9%<br>**Change: 14.5%**<br>**p=0.05***<br>**RR: 1.23 CI 1.03-1.49**			% highest score<br>Pre: 66%<br>Post: 76%<br>**Change: 10% p=0.12**<br>**RR: 1.15 CI 0.96-1.38**		
**Control over patient care**	% completely satisfied<br>Control: 55.6%<br>Intervention: 73.6%<br>**Change: 18% p=0.02***<br>**RR: 1.31 CI 1.07-1.61**					
**Support in decision making**				% highest score<br>Pre: 49%<br>Post: 69%<br>**Change: 20%**<br>**p=0.005***<br>**RR: 1.39 CI 1.09-1.79**		
**Share in decisions about care planning**					% scoring excellent<br>Pre: 45%<br>Post: 68%<br>**Change: 23%**<br>**p=0.009***<br>**RR: 1.52 CI 1.03-2.24**	
**Time to decision**						Days mean (sd)<br>Control 7.4 (3.2)<br>Interv. 6.6 (6.5)<br>**Change -0.8 days**<br>**CI -1.48-3.08**
**Overall metric for comparison**	**RR: 1.31**	**MD: 5.85**	**MD: 3.5**	**RR: 1.39**	**RR: 1.52**	**MD: -0.8**

For peer review only - http://bmjopen.bmj.com/site/about/guidelines.xhtml

Table 9: Depression outcome measures

Study / Intervention / Measure	Garouste et al. 2016 — IP family conference	Prichard & Newcomb 2015 — Hand massage	Curtis et al. 2016 — Communication facilitator
**HADS – Depression**	At 90 days, Median [IQR]<br>Control: 5.5 [1-11.5]<br>Intervention: 2 [0-6]<br>**Change: -3.5 p=0.04***<br>% sign. Depress. score > 8<br>Control: 38.6% (n=17)<br>Intervention: 23.8% (n=10)<br>**Change: 14.8% p=0.14**<br>**RR: 0.61, CI 0.31-1.18**	Mean change in score<br>Control: -0.3<br>Intervention: -2.5<br>**Change: -2.2 p=0.10**<br>**CI -0.49 - 4.7**	
**PHQ 9 Depression**			Mean score 3 months<br>Control: 4.9<br>Intervention: 3.1<br>**Change: -1.8 p=0.09**<br>**CI: -3.89 - 0.31**<br>Mean score 6 months<br>Control: 4.7<br>Intervention: 2.4<br>**Change: -2.3 p=0.01***<br>**CI: -4.30 - -0.42**
**Overall metric**	**MD: -3.5**	**MD: -2.2**	**MD: -1.8**

Table 10: Satisfaction outcome measures

Study / Intervention / Measure	Huang et al. 2018 — Primary physician involvement	Shaw et al. 2014 — Staff teamwork training	Dykes et al. 2017 — ICT-based engagement	Weber et al. 2018 — Family rounds	Huffines et al. 2013 — Supportive care algorithm
**FS ICU 24 – Global score**	Mean (sd)<br>Control: 84.91 (12.17)<br>Intervention: 86.4 (11.76)<br>**Change: 1.49 p=0.16**<br>**CI -2.14 - 5.12**	Mean score<br>Pre: 83.21<br>Post: 85.69<br>**Change: 2.48 p=0.32**	Mean score<br>Pre: 84.3 (3)<br>Post: 90 (1.9)<br>**Change: 5.7 p<0.05***<br>**CI 2.31-9.09**	Mean score (SD)<br>Control: 86.0 (16.0)<br>Intervention: 90.8 (10.7)<br>**Change: 4.8 p=0.20**<br>**CI: -0.12 - 9.72**	
**HCAHPS**			% top score 9-10<br>Pre: 71.8%<br>Post: 93.3%<br>**Change 21.5 P<0.05***<br>**RR 1.33 CI 1.10-1.55**		
**Support given**				% top scores<br>Control: 54%<br>Intervention: 71%<br>**Change: 17% p>0.05**<br>**RR: 1.32 CI 0.99-1.75**	% scoring excellent<br>Pre: 60%<br>Post 75%<br>**Change: 15% p=0.14**<br>**RR: 1.23 CI 0.91-1.65**
**Overall metric for comparison**	**MD: 1.49**	**MD: 2.48**	**RR: 1.33**	**RR: 1.32**	**RR: 1.23**

Study / Intervention / Measure	White et al. 2018 — Multi-component family support programme	Cray 1989 — Family support programme	Dalal et al. 2015 — Patient centred toolkit	Jacobowski et al. 2010 — Family rounds
**PPPC**	Mean score<br>Control: 1.8<br>Intervention: 1.7<br>**Change: -0.15 p=0.006***<br>**CI −0.26 - −0.04**			

For peer review only - http://bmjopen.bmj.com/site/about/guidelines.xhtml

**Satisfaction with intervention**		% Satisfied<br>**100% agreed (76/76)**	% Satisfied<br>**72% (13/18)**	
**Time to ask questions**				% highest score<br>Pre: 40%<br>Post: 23%<br>**Change: -17% p=0.02***<br>**RR: 0.57 CI 0.37-0.90**
**Overall metric for comparison**	**MD: 0.15**	**100%**	**72%**	**RR: 0.57**

Table 11: Knowledge improvement outcome measures

**Study**	**Allen et al. 2017**	**Almoosa et al. 2009**	**Cray 1989**
**Intervention / Measure**	**Family on rounds**	**CPR discussions**	**Family support programme**
**Improved family knowledge**	% agreement<br>Pre: n=146 (35%)<br>Post: n=374 (88%)<br>**Change 52% p<0.001***<br>**RR: 2.46 CI: 2.14-2.81**		
**Questions answered**		% agreement<br>84% (36/43)<br>**CI: 70%-91%**	
**Facilitated decision**		% agreement<br>77% (33/42)<br>**CI: 64%-88%**	
**Understanding of loved one's illness**			% agreement<br>93% (71/76)
**Overall metric for comparison**	**RR: 2.46**	**84%**	**93%**

Table 12: Patient outcome measures

**Study**	**Almoosa et al. 2009**	**White et al. 2018**	**Dykes et al. 2017**
**Intervention / Measure**	**CPR discussions**	**Multi family support**	**Patient engagement**
**Mortality**	Control: 8% (3/39)<br>Intervention: 37% (17/45)<br>**Change: 31%, p<0.05***<br>**RR: 4.91, CI: 1.55-15.51**	Control: 28.5% (249/873)<br>Intervention: 36% (197/547)<br>**Change: 7.5%, p=0.008***<br>**RR: 1.27, CI: 1.09-1.48**	
**Length of stay**		Mean days<br>Control: 13.5<br>Intervention: 10.4<br>**Change: 3.1, p<0.001***<br>**CI: 0.69 - 0.87**	Mean (median) [range]<br>Pre: 4.9 (2) [1-108], n=881<br>Post: 5.0 (2) [1-115, n=904<br>**Change: 0.1 [0], p=0.61**
**Adverse events**			Pre: 59/1000 patient days<br>Post: 42/1000 patient days<br>**Change: 17, % reduction: 29%**<br>**CI: 6.95-27.05, p<0.05***
**Overall metric for comparison**	**RR: 4.91**	**RR: 1.27**	**29%**

For peer review only

Table 13: Satisfaction outcome measures

Measure \ Intervention \ Study	Allen et al. 2017
	**Family presence on rounds**
**Nurse satisfaction**	Pre: n=32 (65%)<br>Post: n=45 (96%)<br>**Change 31% p<0.05***<br>**RR: 1.46, CI: 1.18-1.81**
**Physician satisfaction**	Pre: n=4 (80%)<br>Post: n= 5 (83%)<br>**Change 3% p>0.05**<br>**RR: 1.04, CI: 0.59-1.83**
**Overall metric for comparison**	**RR: 1.46**

Table 14: Teamwork outcome measures

Measure \ Intervention \ Study	Choi et al 2013	Weber et al. 2018	Huffines et al. 2013
	**Patient/ family centred unit design**	**Family rounds**	**Supportive care algorithm**
**Family interactions with patients**	Correlation<br>r=0.632<br>p<0.01*		
**Family interactions with staff**	Correlation<br>r=0.552<br>p<0.01*		
**Coordination of care**		% top scores<br>Control: 68%<br>Intervention: 77%<br>**Change: 9%**<br>**RR: 1.13 CI 0.91-1.41**	
**Nurse – doctor teamwork**			Mean satisfaction (1-4)<br>Pre: 3.38<br>Post: 3.73<br>**Change: 0.35 p=0.04**<br><br>% scoring excellent<br>Pre: 64%<br>Post: 83%<br>**Change: 19% p=0.04**<br>**RR: 1.31 CI 1.01-1.71**
**Overall metric for comparison**	**n/a**	**RR: 1.13**	**RR: 1.31**

For peer review only - http://bmjopen.bmj.com/site/about/guidelines.xhtml

Table 15: Trauma outcome measures

Study / Intervention / Measure	Garouste et al. 2016	Curtis et al. 2016	White et al. 2018
	Interprofessional family conference	Communication facilitator	Multicomponent family support
**PDEQ**	Median [IQR]<br>Control: 14.5 [11-23]<br>Intervention: 13 [0-17]<br>**Change: -1.5 p=0.17**		
**IES-R**	At 90 days, Median [IQR]<br>Control: 24 [12.5-45]<br>Intervention: 21 [9-33]<br>**Change: -3 p=0.24**		
**IES - PTSD (0-88)**		Mean score<br>Control: 20.3<br>Intervention: 21.2<br>**Change: 0.90 p=0.49**<br>**CI: 1.66 - 3.47**	
**PCL - PTSD**			Mean score 3 months<br>Control: 31.6<br>Intervention: 29.8<br>**Change: -1.7 p=0.47**<br>**CI: -6.65 - 3.12**<br><br>Mean score 6 months<br>Control: 30.6<br>Intervention: 27.1<br>**Change: -3.5 p=0.056**<br>**CI: -7.12 - 0.09**
**Overall metric for comparison**	**MD: -3**	**MD: 0.9**	**MD: -7.12**

For peer review only

Figure 1: PRISMA Flow diagram

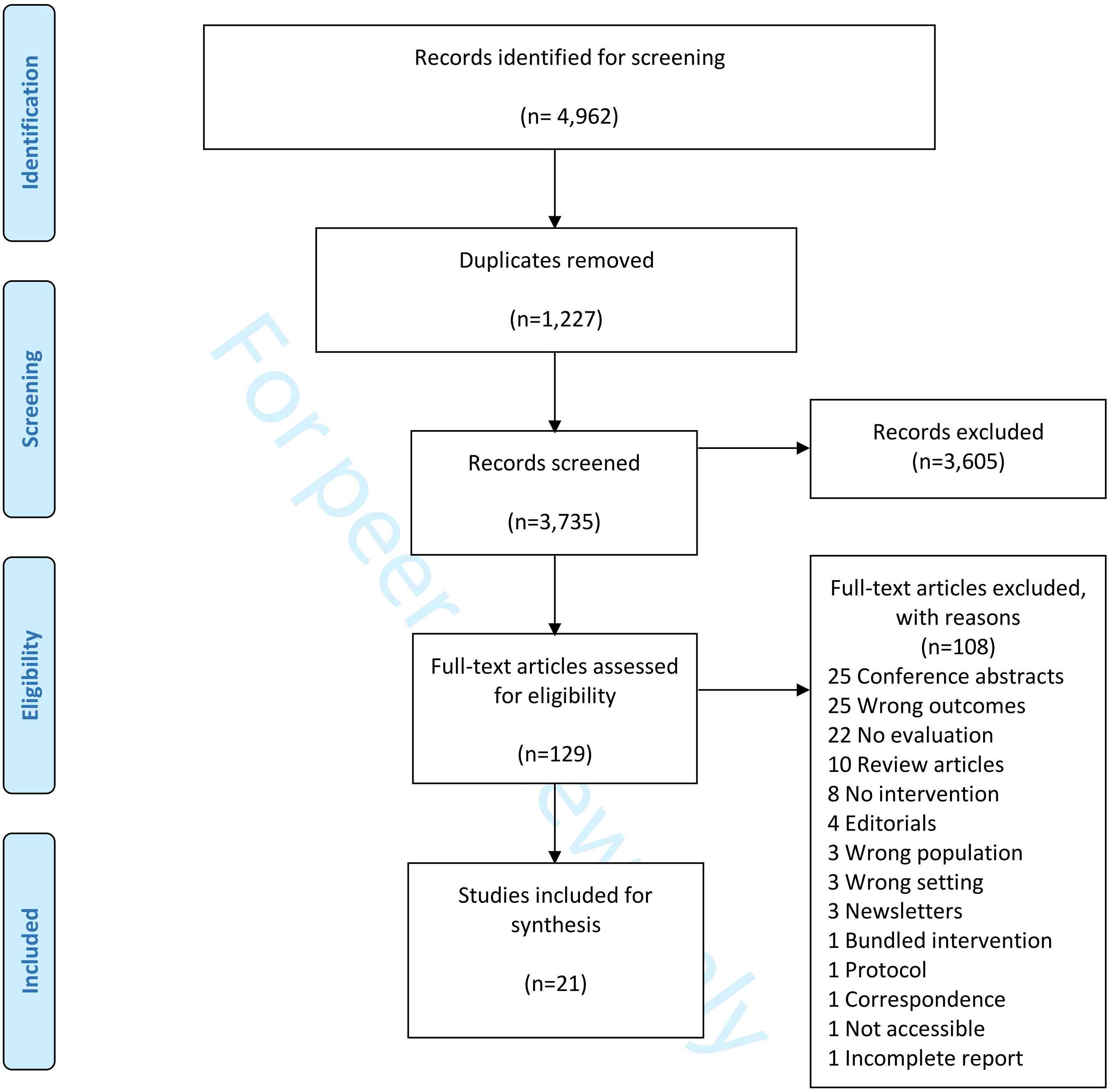

For peer review only - http://bmjopen.bmj.com/site/about/guidelines.xhtml

Kingston University London

St George's University of London

Florence Nightingale Faculty of Nursing, Midwifery & Palliative Care

King's College London

NHS National Institute for Health Research

Features / Intervention Types	ICU setting	Family time commitment	Professional input	Opportunities & Challenges	Cost	Evidence level	Engagement outcomes			Clinical outcomes		
							Satisfaction	Decision making	Communication	Wellbeing	Trauma	Patient outcomes
**Environmental unit changes** Unit change interventions consisted of complete structural redesign of an existing ICU with the objective being enhanced family member presence	General	Low	Low	+ Communication<br>+ Interaction<br>- Exposure<br>- Privacy	High	Low	Low impact	Very low impact	Low impact	Very low impact	Very low impact	Very low impact
**Web-based support** Web based and electronic interventions were approaches which utilised information and communication technology to facilitate information sharing and asynchronous communication	General/ specialist	Moderate	Moderate	+ Knowledge<br>+ Efficiency<br>- IT proficiency	Moderate	Moderate	High impact	Low impact	Very low impact	Very low impact	Very low impact	Low impact
**Discussion-based support** Discussion based interventions generally consisted of one off or regular face to face conversations which took place in the unit between family members and healthcare professionals	General/ Specialist	Moderate	High	+ Support<br>+ Wellbeing<br>+ Decision making<br>- Training	Moderate	High	Moderate impact	Very high impact	High impact	Moderate impact	Moderate impact	Low impact
**Multicomponent support** Multicomponent interventions comprised more than one technique to engage, educate and communicate with family members in the ICU	General/ Specialist	High	High	+ Family trauma<br>+ Pt outcomes<br>- Complexity<br>- Resources	High	High	Moderate impact	Very low impact	Low impact	Moderate impact	Low impact	Moderate impact
**Participation in medical rounds** Family involvement in rounds interventions enabled the family members to attend and watch the daily rounds process, with opportunity to raise questions and clarify issues	General ICU	High	Moderate	+ Knowledge<br>+ Efficiency<br>- Time<br>- Satisfaction	Low	High	Moderate impact	Very low impact	High impact	Low impact	Very low impact	Very low impact
**Participation in physical care** Physical participation in care interventions consisted of physical touch between family members and patients being utilised for reciprocal benefit	General ICU	High	Low	+ Satisfaction<br>+ Wellbeing<br>- Confidence	Low	Low	High impact	Very low impact	Very low impact	Moderate impact	Very low impact	Very low impact

Reporting checklist for systematic review and meta-analysis.

Based on the PRISMA guidelines.

Instructions to authors

Complete this checklist by entering the page numbers from your manuscript where readers will find each of the items listed below.

Your article may not currently address all the items on the checklist. Please modify your text to include the missing information. If you are certain that an item does not apply, please write "n/a" and provide a short explanation.

Upload your completed checklist as an extra file when you submit to a journal.

In your methods section, say that you used the PRISMAreporting guidelines, and cite them as:

Moher D, Liberati A, Tetzlaff J, Altman DG, The PRISMA Group. Preferred Reporting Items for Systematic Reviews and Meta-Analyses: The PRISMA Statement

For peer review only

		Reporting Item	Page Number
**Title**			
	#1	Identify the report as a systematic review, meta-analysis, or both.	1
**Abstract**			
Structured summary	#2	Provide a structured summary including, as applicable: background; objectives; data sources; study eligibility criteria, participants, and interventions; study appraisal and synthesis methods; results; limitations; conclusions and implications of key findings; systematic review registration number	2
**Introduction**			
Rationale	#3	Describe the rationale for the review in the context of what is already known.	4
Objectives	#4	Provide an explicit statement of questions being addressed with reference to participants, interventions, comparisons, outcomes, and study design	5

		(PICOS).	
**Methods**			
Protocol and registration	#5	Indicate if a review protocol exists, if and where it can be accessed (e.g., Web address) and, if available, provide registration information including the registration number.	5
Eligibility criteria	#6	Specify study characteristics (e.g., PICOS, length of follow-up) and report characteristics (e.g., years considered, language, publication status) used as criteria for eligibility, giving rational	5
Information sources	#7	Describe all information sources in the search (e.g., databases with dates of coverage, contact with study authors to identify additional studies) and date last searched.	5
Search	#8	Present full electronic search strategy for at least one database, including any limits used, such that it could be repeated.	5
Study selection	#9	State the process for selecting studies (i.e., for screening, for determining eligibility, for inclusion in the systematic review, and, if applicable, for inclusion in the meta-analysis).	5-6
Data collection process	#10	Describe the method of data extraction from reports (e.g., piloted forms, independently by two reviewers) and any processes for obtaining and confirming data from investigators.	5-6
Data items	#11	List and define all variables for which data were sought (e.g., PICOS, funding sources), and any assumptions and simplifications made.	5-6
Risk of bias in individual studies	#12	Describe methods used for assessing risk of bias in individual studies (including specification of whether this was done at the study or outcome level, or both), and how this information is to be used in any data synthesis.	5-6
Summary measures	#13	State the principal summary measures (e.g., risk ratio, difference in means).	5-6
Planned methods of analyis	#14	Describe the methods of handling data and combining results of studies, if done, including measures of consistency (e.g., I2) for each meta-analysis.	6
Risk of bias across studies	#15	Specify any assessment of risk of bias that may affect the cumulative evidence (e.g., publication bias, selective reporting within studies).	5-6
Additional	#16	Describe methods of additional analyses (e.g., sensitivity or subgroup	6

For peer review only

analyses		analyses, meta-regression), if done, indicating which were pre-specified.	
**Results**			
Study selection	#17	Give numbers of studies screened, assessed for eligibility, and included in the review, with reasons for exclusions at each stage, ideally with a flow diagram.	6
Study characteristics	#18	For each study, present characteristics for which data were extracted (e.g., study size, PICOS, follow-up period) and provide the citation.	6-7
Risk of bias within studies	#19	Present data on risk of bias of each study and, if available, any outcome-level assessment (see Item 12).	7
Results of individual studies	#20	For all outcomes considered (benefits and harms), present, for each study: (a) simple summary data for each intervention group and (b) effect estimates and confidence intervals, ideally with a forest plot.	7-8
Synthesis of results	#21	Present the main results of the review. If meta-analyses are done, include for each, confidence intervals and measures of consistency.	8-9
Risk of bias across studies	#22	Present results of any assessment of risk of bias across studies (see Item 15).	9-10
Additional analysis	#23	Give results of additional analyses, if done (e.g., sensitivity or subgroup analyses, meta-regression [see Item 16]).	9-10
**Discussion**			
Summary of Evidence	#24	Summarize the main findings, including the strength of evidence for each main outcome; consider their relevance to key groups (e.g., health care providers, users, and policy makers	10-11
Limitations	#25	Discuss limitations at study and outcome level (e.g., risk of bias), and at review level (e.g., incomplete retrieval of identified research, reporting bias).	10-11
Conclusions	#26	Provide a general interpretation of the results in the context of other evidence, and implications for future research.	11
**Funding**			
Funding	#27	Describe sources of funding or other support (e.g., supply of data) for the systematic review; role of funders for the systematic review.	11

For peer review only

The PRISMA checklist is distributed under the terms of the Creative Commons Attribution License CC-BY.
This checklist was completed on 08. July 2020 using https://www.goodreports.org/, a tool made by the EQUATOR Network in collaboration with Penelope.ai

For peer review only

Interventions to promote family member involvement in adult critical care settings: a systematic review.

Journal:	*BMJ Open*
Manuscript ID	bmjopen-2020-042556.R1
Article Type:	Original research
Date Submitted by the Author:	02-Nov-2020
Complete List of Authors:	Xyrichis, Andreas; King's College London,<br>Fletcher, Simon; Kingston and St Georges University London, Health, Social Care and Education<br>Philippou, Julia; King's College London, Nursing & Midwifery<br>Brearley, Sally; King's College London, Florence Nightingale Faculty of Nursing and Midwifery<br>Terblanche, Marius; King's College London,<br>Rafferty, Anne Marie; King's College London, Florence Nightingale School of Nursing and Midwifery
**Primary Subject Heading**:	Intensive care
Secondary Subject Heading:	Intensive care, Patient-centred medicine, Health services research, Health policy, Evidence based practice
Keywords:	Adult intensive & critical care < INTENSIVE & CRITICAL CARE, Quality in health care < HEALTH SERVICES ADMINISTRATION & MANAGEMENT, Health policy < HEALTH SERVICES ADMINISTRATION & MANAGEMENT

I, the Submitting Author has the right to grant and does grant on behalf of all authors of the Work (as defined in the below author licence), an exclusive licence and/or a non-exclusive licence for contributions from authors who are: i) UK Crown employees; ii) where BMJ has agreed a CC-BY licence shall apply, and/or iii) in accordance with the terms applicable for US Federal Government officers or employees acting as part of their official duties; on a worldwide, perpetual, irrevocable, royalty-free basis to BMJ Publishing Group Ltd ("BMJ") its licensees and where the relevant Journal is co-owned by BMJ to the co-owners of the Journal, to publish the Work in this journal and any other BMJ products and to exploit all rights, as set out in our licence.

The Submitting Author accepts and understands that any supply made under these terms is made by BMJ to the Submitting Author unless you are acting as an employee on behalf of your employer or a postgraduate student of an affiliated institution which is paying any applicable article publishing charge ("APC") for Open Access articles. Where the Submitting Author wishes to make the Work available on an Open Access basis (and intends to pay the relevant APC), the terms of reuse of such Open Access shall be governed by a Creative Commons licence – details of these licences and which Creative Commons licence will apply to this Work are set out in our licence referred to above.

Other than as permitted in any relevant BMJ Author's Self Archiving Policies, I confirm this Work has not been accepted for publication elsewhere, is not being considered for publication elsewhere and does not duplicate material already published. I confirm all authors consent to publication of this Work and authorise the granting of this licence.

For peer review only

TITLE PAGE

Interventions to promote family member involvement in adult critical care settings: a systematic review.

Andreas Xyrichis (corresponding author): Kings College London, James Clerk Maxwell Building, Waterloo, London, andreas.xyrichis@kcl.ac.uk
Simon Fletcher: Kingston & St Georges University London, Hunter Wing 6th Floor, St Georges University, Tooting, London, simon.fletcher@sgul.kingston.ac.uk
Julia Philippou: Kings College London, James Clerk Maxwell Building, Waterloo, London, julia.philippou@kcl.ac.uk
Sally Brearley: Kingston & St Georges University London, Hunter Wing 6th Floor, St Georges University, Tooting, London, sally.brearley@icloud.com
Marius Terblanche: Kings College London, James Clerk Maxwell Building, Waterloo, London, marius.terblanche@kcl.ac.uk
Anne Marie Rafferty: Kings College London, James Clerk Maxwell Building, Waterloo, London, anne_marie.rafferty@kcl.ac.uk

For peer review only

ABSTRACT

Objective: To identify, appraise and synthesise evidence of interventions designed to promote family member involvement in adult critical care units; and to develop a working typology of interventions for use by health professionals and family members.

Design: Mixed-method systematic review.

Data sources: Bibliographic databases were searched without date restriction up to June 2019: MEDLINE, EMBASE and CINAHL; the Cochrane Central Register of Controlled Trials, Joanna Briggs and Cochrane Libraries. Back issues of leading critical care and patient experience journals were manually searched, as were the reference lists of included studies. All evaluation studies of relevant intervention activities were included; all research designs and outcome measures were eligible. Due to heterogeneity in interventions, designs and outcome measures the synthesis followed a narrative approach. Service users met with the research team termly.

Results: Out of 4,962 possible citations a total of 20 studies were included. The overall evidence base was assessed as moderate to weak. Six categories of interventions were identified: environmental unit changes (n=2), web-based support (n=4), discussion-based support (n=6), multicomponent support (n=4), participation in rounds (n=3), and participation in physical care (n=1). Clinical and methodological heterogeneity across studies hindered meta-analysis, hence a narrative synthesis was pursued. Six main outcomes were identified, grouped under two categories: i) Involvement outcomes: Communication (*mean difference* ranged from: 6.39 to 8.83), Decision making (*mean difference* ranged from: -0.8 to 5.85), Satisfaction (*mean difference* ranged from: 0.15 to 2.48); and ii) Health outcomes: Family Trauma (*mean difference* ranged from: -7.12 to 0.9), Family Wellbeing (*mean difference* ranged from: -0.7 to -4), Patient outcomes (*relative risk* ranged from: 1.27 to 4.91). The findings from the qualitative studies were thematically analysed to identify features of the interventions that participants perceived to influence effectiveness. Synthesised into five overarching categories (practicality, development, interaction, reflexivity, bridging) these can serve as principles to inform the future design and development of more refined family member involvement interventions.

Conclusions: Future interventions should be developed with much closer family member input and designed by considering the key features we identified. We call for future interventions to be multi-layered and allow for a greater or lesser level, and different kind, of involvement for family members. Choice of intervention should be informed by a baseline diagnostic of family members' needs, readiness and preparedness for involvement.

Review registration: PROSPERO 2018 CRD42018086325

Keywords: Critical care units, family involvement, systematic review, typology, service user

For peer review only - http://bmjopen.bmj.com/site/about/guidelines.xhtml

ARTICLE SUMMARY

Strengths and limitations of this study

- We completed a comprehensive mixed-method systematic review of available evidence on interventions for family member involvement in adult critical care.
- We involved a Service Users & Carers group throughout the systematic review process, which included 16 hours of in-depth group discussions.
- Study screening, selection, quality assessment and data extraction was completed independently and in duplicate by two review authors.
- Methodological and clinical heterogeneity across studies prevented us from pursuing a meta-analysis.
- Qualitative evidence was synthesised thematically to identify features of the interventions that participants perceived to influence effectiveness.

For peer review only

For peer review only - http://bmjopen.bmj.com/site/about/guidelines.xhtml

BACKGROUND

Family member involvement in healthcare is recognised by policymakers and the public as an invaluable aspect of healthcare provision. The move towards the more structured integration of family members is consistent with the call for greater patient-centred care, long invoked by a (2001) report from the Institute of Medicine[1]. Emphasising the potential benefits in actively involving family members in decision-making processes and care management was regarded as a key contributor to the reduction of the risk of medical error and a broader improvement in quality. The idea that a 'fully-engaged' public[2] should be encouraged has also been evident in United Kingdom contexts, however governmental scrutiny over the proceeding decade has revealed that this has yet to be comprehensively realised. Two strategic reports from NHS England[3,4] explored a practical move towards a healthcare system which is built around the patient, incorporating families representing a key means of achieving this.

For the purposes of the present review, family member involvement refers to the activities of different professionals to improve care by ensuring family members are involved in decision making, sharing of information, power and responsibility for patient needs and choices. Family member involvement is especially relevant in the intensive care unit (ICU), where it can have profound consequences for care decisions, delivery and outcomes; this is partly because ICU patients are rarely in a position to communicate directly with clinicians and recollect their ICU experience, which means that the responsibility for this often lies with their family members. While this is a concern in healthcare systems worldwide, and especially in the UK, a recent mixed-methods study found there is still great variation in family satisfaction across ICUs in England, Wales and Northern Ireland[5].

Studies over the past decade have confirmed that family members can have a positive influence on a patients' care and recovery from ICU; but also that family members themselves can be affected even after discharge[6-11]. In particular, within the first few days after ICU admission, family members can show signs of anxiety, depression and stress; report difficulties in understanding the information clinicians try to communicate with them; and those who suffered a bereavement are at risk of generalized anxiety, panic attacks, depression, and post-traumatic stress syndrome (PTSD)[7]. For example, one study[6] surveyed family members of patients who have been in ICU to find that 90 days after discharge more than a third (34%) of them suffered from PTSD symptoms. In addition, they noted higher rates (48%) among those family members who indicated the information they were given was incomplete.

While there is a growing evidence base on patients, family members and clinicians' perceptions of involvement in ICU care, we are still missing a standardised, evidence-based approach to facilitate this process. Indeed, a recent scoping review[10] sought to investigate the extent and range of literature on this topic, finding evidence of a growth in papers with over 100 reports identified. However, they did not seek to assess the quality of the evidence nor did they examine the type of interventions available, and key features that foster

For peer review only - http://bmjopen.bmj.com/site/about/guidelines.xhtml

effectiveness. While this is an area of growing interest, we are still unclear about the range and quality of interventions available to promote family member involvement in ICUs.

METHODS

Our review sought to answer the following question: What are the available interventions, and which are most effective, for fostering family member involvement in adult critical care settings? Four objectives were set: a) undertake a comprehensive and systematic search of published and unpublished studies reporting on interventions that promote involvement in adult critical care; b) robustly assess the quality of empirical evidence for all included studies; c) generate a detailed description and synthesis of interventions and their associated outcomes; and d) classify interventions and outcomes in order to develop a typology of interventions, outlining key factors that support or impede involvement.

Study eligibility criteria

We included evaluation studies of any design, including experimental and quasi-experimental studies, as well as action research, case study and ethnographic designs. We considered reports of any kind of interventions as long as they were intended to promote the participation of family members in adult critical care. We included studies that reported on a mixture of relevant outcomes, including standard measures such as the Hospital Anxiety and Depression Score (HADS); and non-standard but important indicators such as family satisfaction. We excluded non-evaluation papers and those with populations, outcomes and settings that did not match our brief. One study reported on a bundled intervention including awakening and breathing coordination, delirium monitoring and management, early mobility, and family-centred care; this study had to be excluded because we could not discern outcomes specifically linked to the family involvement component of the bundle.

Search for evidence

We searched (June 2019) MEDLINE, EMBASE, CINAHL and the Cochrane Central Register of Controlled Trials following a systematic approach without date restriction. We also performed searches for clinical studies through the WHO Trials Registry. We hand searched recent back issues of key critical care and patient experience e-journals, scanned reference lists of identified reviews and included articles; searched our personal libraries and consulted with experts in the field. GoogleScholar was also searched. Non-commercially published (Grey) literature was sought through the OpenGrey and the GreyLit Report databases. A standardised search strategy was developed with the input of an information specialist and applied to all databases, utilising both MeSH and freetext terms (Supplementary file).

Data management, screening, extraction and quality assessment

Search results were imported into Covidence, the standard production platform for Cochrane Reviews. Two reviewers (AX, SF) independently screened all citations in duplicate against the inclusion criteria. Any disagreement was resolved through discussion or by involving a third

For peer review only - http://bmjopen.bmj.com/site/about/guidelines.xhtml

reviewer (JP). Reasons for excluding papers read in full were recorded and a PRISMA flowchart was completed. Standardised data extraction forms were used by two reviewers (SF, AX) to independently extract key information. Any disagreements found at this stage were also resolved through a consensus approach, or the involvement of a third reviewer (JP). We assessed each included paper for methodological rigour using an established rating approach utilised successfully in a number of previous mixed-method systematic reviews[12,13]. This rating approach assesses 'quality of study' to consider: appropriate fit between study design/research questions and use of appropriate analyses; and, 'quality of information' to assess for a clear rationale for the intervention, good contextual information and risk of bias. Quality assessment was undertaken independently by two reviewers (AX, SF) in duplicate. For each quality domain we described the procedures reported in the paper, using verbatim quotes as necessary.

Data synthesis

The overall approach to the synthesis followed a segregated methodologies approach[14]. Accordingly, a separate synthesis of quantitative and qualitative data was initially completed followed by a mixed-method synthesis. Quantitative studies were grouped depending on study design, and results summarised using descriptive statistics. Because heterogeneity precluded statistical combination through a meta-analysis, a narrative approach to the quantitative synthesis was pursued. Synthesis of qualitative data followed the Best-fit framework approach, involving identification and grouping of thematic categories around factors influencing effectiveness of family member involvement interventions[15]. The mixed-method synthesis utilised the quantitative data to inform on the measured effects, while the qualitative data informed on the perceived effects. In this way, the qualitative and quantitative data acted as complementary rather than as confirmatory of each other. A key element of the mixed-method synthesis was to classify interventions and outcomes in order to develop a typology of family involvement interventions in ICU. To develop the typology, we identified categories for the different interventions used in the studies by noting their distinguishing characteristics. We also formed and refined key family involvement features of the interventions, such as required time commitment from families and staff, opportunities and challenges, and cost. We tabulated these alongside an indication of the quality level of the current body of evidence, and the expected impact on important engagement and clinical outcomes. The typology was developed in collaboration with a Service Users & Carers Group over two face to face meetings, during which the overall findings of the review and ways of representing these visually were discussed. The resulting typology is illustrated in a matrix-like format presenting the intervention categories in a continuum from low to high involvement.

Patient and public involvement

A Service Users & Carers Group was established from the outset, the members of which were recruited through promotion in social media, the UK ICUSteps charity and the NIHR INVOLVE website. Consisting of eight ICU survivors and carers, the group was consulted through each

For peer review only - http://bmjopen.bmj.com/site/about/guidelines.xhtml

step of the process and engaged in four strategically positioned meetings leading to over 16 hours of focussed and in-depth face-to-face discussions concerning the design, conduct, reporting and dissemination of the review.

RESULTS

Overview of included studies

Search results

Out of 4,962 possible citations a total of 20 studies were included (Figure 1). Published between 1989 and 2018, they were predominantly from North American contexts (n=17), and from a range of ICU settings including surgical, neuroscience, medical oncology and trauma ICUs. One study came from a French setting, and others from Swedish and Australian ICU settings.

Study designs

Most of the studies followed a quantitative approach (n=15), but we also included four qualitative and one mixed methods study (see Table 1). Specifically, quantitative studies included randomised controlled trials, cluster-randomised and stepped-wedge trials, non-randomised before and after studies, and prospective observational multi-centre studies. Qualitative data from all studies came from semi-structured interviews. Sample sizes across the quantitative studies ranged from 30 to 1,106 family members; and in the qualitative studies from 10 to 54 family members.

Intervention types

Interventions were grouped based on their distinguishing characteristics (summarised in Table 2), and positioned within a continuum of low to high involvement: i) environmental unit changes (n=2)[16-17], ii) web-based support (n=4)[18-21], iii) discussion-based support (n=6)[22-27], iv) multicomponent support (n=4)[28-31], v) participation in rounds (n=3)[32-34], and vi) participation in physical care (n=1)[35]. Environmental unit change interventions consisted of complete structural redesign of an existing ICU with the specific objective being enhanced family member presence. Web based and electronic interventions were approaches which utilised information and communication technology to facilitate information sharing and asynchronous communication. Discussion-based interventions generally consisted of one off or repeated face-to-face conversations which took place in the unit between family members and healthcare professionals. Multi-component interventions comprised more than one technique to engage, educate and communicate with family members in the ICU. Family involvement in rounds interventions enabled the family members to attend and watch the daily rounds process, with opportunity to raise questions and clarify issues. Physical participation in care consisted of physical touch between family members and patients being utilised for reciprocal benefit.

For peer review only - http://bmjopen.bmj.com/site/about/guidelines.xhtml

Quality of evidence

The overall evidence base was assessed to be moderate to weak, with only a few exceptions of high-quality quantitative and qualitative studies (Tables 3 and 4). The main weaknesses of the quantitative studies were related to inadequate randomisation and blinding, and lacking a control group for comparison purposes. The qualitative studies were in the main of a better quality, with key weaknesses being related to inadequate researcher reflexivity and theorisation of findings.

Quantitative results

Six main outcome categories were identified from the studies analysed for the review, grouped under two groups. Involvement outcomes: Communication, Decision making, Satisfaction; and Health outcomes: Family Wellbeing, Family Trauma, Patient outcomes. A summary of key results is provided below, with detailed reporting of outcome measures available in the tables; the supplement offers additional tables in which outcome measures are presented according to intervention type.

Involvement outcomes

Communication (Table 5): Improved communication was considered as an outcome in five studies[32,33,27,34,31], the majority of which involved family participation in rounds, reporting on four measurement tools. Three studies[33,27,34] utilised the Family Satisfaction ICU (FS ICU 24) tool, specifically reporting on three dimensions: honesty of information, frequency of doctor communication, frequency of nurse communication. One study[31] reported on the Quality of Communication (QOC) tool and another used a self-developed questionnaire[32]. Mean improvements were found in all studies, although not all results reached statistical significance. The biggest effect was seen on the frequency of doctor communication, with one study of family participation in rounds showing a statistically significant improvement of 60% (RR: 1.60, CI 1.18 to 2.17, p=0.004)[33].

Decision making (Table 6): Six of the included studies[22,25,21,33,27,34] looked at decision making as an outcome, most of which were discussion-based interventions, through six measurement tools. The 'decision making' sub-scale of the FS ICU 24 revealed improvements in three studies although only one[27], targeting the whole interprofessional team, found a statistically significant result (MD: 5.85, p=0.05). Inclusion in decision making was also considered, as the percentage change in family members reporting complete satisfaction or giving the highest score, with one study[25] which involved the patient's primary physician showing a statistically significant improvement of 23% (RR: 1.23, CI: 1.03 to 1.49, p=0.05). Studies also noted improvements with regard to 'control over patient care' (RR: 1.31, CI: 1.07 to 1.61, p=0.02)[25], 'support in decision making' (RR: 1.39, CI: 1.09 – 1.79)[21] and 'share in decisions about care planning' (23%, RR: 1.52, CI: 1.03 to 2.24, p=0.009)[21].

For peer review only - http://bmjopen.bmj.com/site/about/guidelines.xhtml

Satisfaction (Table 7): Satisfaction was considered as an outcome in nine studies [28,18,19,25,21,33,27,34,31] using six different measures, and reporting on a mixture of discussion-, rounds-, multi-component- and web- based interventions. The majority of studies (n=4) measured family satisfaction with care using the FS-ICU, while the remaining studies used self-developed tools. One of the studies[19] also used the HCAHPS (Hospital Consumer Assessment of Healthcare Providers and Systems) Survey. This is a national, standardized, publicly reported survey of patients' perspectives of hospital care in the US. Mean improvements on the FS ICU were found in all studies, ranging from 1.49 to 5.7, although only one[19] which used a web-based engagement intervention reached statistical significance (MD: 5.7, CI: 2.31 to 9.09, p<0.05). Statistically significant improvements were also noted on the HCAHPS (RR: 1.33, CI: 1.10 to 1.55, p<0.05)[19].

Health outcomes

Family wellbeing was considered through measurements for anxiety and depression.

Anxiety (Table 8): Four studies, which encouraged family participation in discussions and personal care, considered anxiety as an outcome[23,35,26,31] using three different ways for measurement. Three studies[23,35,31] used the HADS. The fourth study[26] used the Generalised Anxiety Disorder Assessment (GAD-7). All four studies identified a reduction in family members' anxiety scores post the intervention, with the effect ranging from -0.34 to -4. The minimal clinically important difference of HADS has been suggested to be between -1.5 and -2, therefore the reduction noted in these papers could be of clinical significance. However, only two of these studies found statistical significance[23,35].

Depression (Table 9): Depression was an outcome also reported by three of the above studies[23,35,26] using two measures. Two studies[23,35] used the depression subscale of the Hospital Anxiety and Depression Scale (HADS) while another[26] used the Patient Health Questionnaire (PHQ-9). Both studies that used the HADS identified a reduction in depression among family members, although only one[23] reached statistical significance (p=0.04) with a median reduction of 3.5. Results on the PHQ-9 also showed a statistically significant mean reduction in scores at six months (MD: -2.3, CI: -4.30 to -0.42, p=0.01)[26].

Family Trauma (Table 10): Three studies considered the impact of interventions (multicomponent-, web- and discussion-based) on family member ICU-induced trauma using four different measurement tools[23,26,31]: Post-Traumatic Stress Disorder (PTSD) Checklist (PCL), Impact of Event Scale (IES) and Peritraumatic Dissociative Experience Questionnaire (PDEQ). All three studies identified a trend showing a beneficial effect of family involvement interventions on severity of trauma through reduced severity scores, ranging from a reduction of -3 to -7 points on the various scales, however none of these results reached statistical significance.

Patient outcomes (Table 11): Three kinds of patient outcomes were reported in three studies involving multicomponent-, web- and discussion-based interventions: patient mortality[22,31] adverse events[19] and length of stay[19,31]. A statistically significant

For peer review only - http://bmjopen.bmj.com/site/about/guidelines.xhtml

increase in mortality was reported in two studies; specifically one study found an actual percentage increase in the intervention group of 31% (RR: 4.91, CI: 1.55 to 15.51, p<0.05)[22] while the other of 7.5% (RR: 1.27, CI: 1.09 to 1.48, p=0.008)[31]. A relative reduction in adverse events per 1000 patient days of 29% was also reported[9] with an actual mean difference of -17 (CI: 6.95 to 27.05, p<0.05). Length of stay was examined in two studies with conflicting results: one reported a statistically significant reduction (MD: -0.77, CI: -0.69 to -0.87, p<0.001)[31] while another did not find any statistically significant difference[19].

Qualitative results

Features that influence effectiveness

The findings from the qualitative studies (n=5) were analysed thematically to identify features of the interventions that participants perceived to influence effectiveness. These included accessibility, simplicity, supplementarity, contextualisation, interprofessionality, consistency, relationship and confidence development. Synthesised into five overarching categories, these can serve as principles to inform the future design and development of more refined family member involvement interventions in ICU: a) Practicality, b) Development, c) Interaction, d) Reflexivity and e) Bridging. We present a high-level summary of these below, with a more detailed qualitative report to be made available in a forthcoming publication.

Practicality: Identified as a theme in three studies[20,23-24] this included intervention accessibility, simplicity, long term application and repetition. Practicality points to effective interventions needing to be readily available with clear instructions, which are as close to universally identifiable as possible. This may enable the intervention to be used as many times as necessary, benefiting family members over and beyond an individual encounter.

Development: In four studies[17,20,24,30] developing family members' contextual knowledge, or insight, was particularly valuable for effective intervention delivery. Insight was key to developing shared understanding among all those engaged in the treatment process. Insight should go beyond an understanding of the patient's condition to encompass the diverse perspectives of healthcare professionals and the emotional strain which family members naturally undergo.

Interaction: Three studies[17,20,24] noted that interventions, either explicitly or implicitly, should encourage verbal and physical interaction between clinicians and family members. However, interprofessional discussions among ICU clinicians, and specifically between medical and nursing staff, appear equally important. Interventions that foster quality interaction at all levels appear essential for the family to be coherently integrated into the structure of the ICU.

Reflexivity: While effective interventions bring people together, three studies[17,23-24] noted they should also benefit the individual family member, who has the opportunity for personal growth in the path towards meaningful family member integration. Specific

For peer review only

examples of reflexivity in the featured interventions were identified in the confirmatory exchanges between family members and healthcare professionals, as family members were able to explore their own role within the ICU, the relationships which they had independently developed with their clinicians and how they could utilise this unique knowledge as a form of leverage in their communication with ICU professionals.

Bridging: Effective interventions should also provide key and otherwise unavailable pathways to encourage family member integration. In two studies[20,23] the supplementary capacity which effective interventions offer was of particular value when consistent or predictable communication could not be guaranteed. While information availability is important, acknowledging the many difficulties which family members themselves face, and offering ways to overcome these, may ultimately enhance integration and experience.

Proposed typology

Utilising the learning gained from the current review, and the insights of our service users and carers group, we propose a typology of interventions (Figure 2) to be considered when promoting family member involvement in ICU. Presented in a matrix, we position types of interventions along a continuum of low to high involvement (environmental unit changes, web-based support, discussion-based support, multicomponent support, participation in rounds and participation in physical care) along with their key characteristics (type of ICU, family time commitment, professional input, cost, opportunities & challenges), level of evidence (low, moderate, high) and impact on key involvement and health outcomes (satisfaction, decision making, communication, family wellbeing, family trauma, and patient outcomes).

We completed an initial face and content validation with our project's service users group consisting of ICU survivors and family members, and advisory board consisting of ICU professionals, but would still caution that the typology remains in need of further empirical validation in different ICU settings. At this stage, we suggest the typology is used as a reflective tool to aid ICU teams make evidence-informed decisions about potential interventions they may wish to consider adopting locally. In the first instance, teams are advised to discuss the kinds of engagement and/or clinical outcomes their unit wants to improve as a priority so they can focus and limit their choice of intervention types. They could then consider the evidence level, cost and key opportunities and challenges offered by each intervention type to help them make fully informed decisions as well as manage expectations. Finally, they could consider the time investment expected of ICU professionals and of family members for the different intervention types; for example, in geographically remote areas where family visiting is a challenge and staff shortage is significant, the team may favour trialling an intervention with less time commitment.

We expect our typology to introduce some much-needed clarity in this field of research, as well as inform the development of future interventions and research studies. Importantly, the

For peer review only - http://bmjopen.bmj.com/site/about/guidelines.xhtml

For peer review only

typology may be used by individual ICUs to inform discussions among professionals, and with family members, about which kinds of family involvement interventions they may wish to adopt, trial, and implement in their units.

DISCUSSION

The diverse nature of the family involvement interventions we identified, the heterogeneity of research designs found, and the varying methodological quality of available studies meant there was not a single intervention that stood out as the recommended way forward. Overall, the available evidence suggests that: rounds-based interventions can benefit communication outcomes; multi-component and web-based interventions can increase family satisfaction; and, discussion-based interventions can benefit decision making and family wellbeing. However, interventions can be of varying cost, require different kinds and levels of input and time commitment from clinicians and family members, and impact on different kinds of outcomes. Given the current state of the evidence, we discourage proposing a single intervention as the 'gold standard' and instead encourage use of our typology to inform decisions based on individual ICU teams' context, needs and available resources.

Through focussing on the different types of available interventions, their associated involvement and clinical outcomes, features perceived to influence effectiveness, and proposing an overarching typology of family involvement interventions, the current review makes a significant contribution to the available literature. Past review work in this area largely focussed on interventions that can support family members of critically ill patients[36-39]; these included providing care and treatment options for family members to counter the negative effects arising out of a loved one being hospitalised, or dying, in ICU. Such work has helped spark the current movement around family involvement in ICU but remains somewhat limiting in that it does not adequately distinguish between family *support* and family *involvement*, often conflating the two. Consequently, conclusions of past work lack clarity on what can be done to involve as well as support family members.

We add to this field of research by adopting strict methodological standards in the current review, to reduce risk of reviewer bias and strengthen transparency. Many of the available guidelines for family involvement in ICU are based on a scoping review methodology, which can be methodologically limiting especially in lacking independent and duplicate screening, data extraction and quality assessment; though with notable exceptions[40]. A further way in which we sought to build on others' work was by examining the full spectrum of involvement interventions, rather than focussing on singular aspects such as decision making. Moreover, we opted to include service users throughout the review process, rather than limiting their involvement to the final stages of peer checking results. This allowed our service users to contribute throughout the process, see how the review was developing, appreciate the technical side of the review process and of the available evidence, and importantly to contribute actively in the development of the final typology.

For peer review only - http://bmjopen.bmj.com/site/about/guidelines.xhtml

For peer review only

Much of the available literature on family involvement tends to conflate and gloss over different kinds of interventions; while this has been helpful in the past to make the case for the importance of family involvement in general, it is time to start to unpick the different types of available interventions and their impact on different kinds of outcomes. Available guidelines often suggest a long list of initiatives to support family members without full consideration or instruction on the contextual requirements of these suggestions or the kind of impact likely to be achieved by these. While prior reviews concluded, as have we, that the evidence in this field needs development and strengthening, these did not offer a granulated analysis of the evidence level for different kinds of interventions or presented these in a visual and user-friendly way to inform future work. We offer our typology as a first step in this direction, towards developing a more systematic programme of research on interventions for family involvement in ICU.

By having an engaged service user and carer group work with us throughout the completion of this review, we were able to look beyond the evidence by exploring the experiences and views of service users about the different interventions identified. This process, which involved four strategically positioned meetings leading to over 16 hours of focussed and in-depth face-to-face discussions, led to us to identify a key, fundamental flaw of existing family involvement interventions and research: in our assessment, these tend to treat family members as a homogeneous group of individuals, rather than as heterogenous units. Specifically, different family members may have different involvement needs and preferences influenced by their previous experiences, knowledge, mental and physical health, and socioeconomic state. In addition, the notion of a family member extends beyond the 'next of kin' or 'personal consultee' to encompass extended family, carers and friends. Moreover, our service users emphasised that the dynamics within family units are crucial to shaping the experience, benefit and satisfaction of family members with ICU care and their level of involvement.

Current family involvement interventions and initiatives appear to be applied in ICUs following a blanket approach, without adequate consideration of particular family members' needs and preparedness for involvement. This may result in some family members benefiting while others experiencing harm, which goes some way in explaining the wide confidence intervals identified in our review that often cross the line of no difference. Indeed, within the PTSD literature, systematic reviews have long concluded that interventions applied universally to victims of trauma, such as compulsory debriefing, should cease and replaced with a 'screen and treat' model[41]. We therefore invite researchers and clinicians to consider the aforementioned issues carefully in future initiatives for involving family members in ICU.

The current review should be considered in the context of its limitations. These include lack of a meta-analysis, and research published in languages other than English and from lower- and middle-income countries.

For peer review only - http://bmjopen.bmj.com/site/about/guidelines.xhtml

CONCLUSION

We examined interventions for family member involvement in ICUs, assessed the quality level of the available evidence, presented key results grouped by kinds of outcomes and interventions, and identified key features stakeholders perceived to influence intervention success. We summarised our results in the form of a typology, which we offer here with a view to sparking further debate, research and applied work in this field. Based on the learning gained from the current review, we argue that future interventions should be developed with much closer family member input and designed by considering the key success features we identified. Importantly, we call for future interventions to be multi-layered to allow for a greater or lesser level, and different kind of, involvement for family members; the decision of which should be informed by a baseline diagnostic of family members' readiness and preparedness for involvement.

DECLARATIONS

Acknowledgement

We are grateful to our Expert Advisory Board and Service User & Carer Group for their invaluable insight and support throughout this review. We owe intellectual debt to the late Professor Scott Reeves who generously supervised this project until his untimely passing in May 2018.

Ethics approval and consent to participate – Not applicable.

Consent for publication – Not applicable.

Availability of data and material – The datasets generated and/or analysed during the current study are available from the corresponding author on reasonable request.

Competing interests – The authors declare that they have no competing interests.

Funding – This paper presents independent research funded by the National Institute for Health Research (NIHR) under its Research for Patient Benefit (RfPB) Programme (Grant Reference Number PB-PG-0416-20021). The views expressed are those of the authors and not necessarily those of the NHS, the NIHR or the Department of Health and Social Care.

Author contributions – AX was responsible for conceiving the review, developing the review question and methodology, leading the funding application and coordinating the project. SF and JP were responsible for developing and executing the search strategy. AX, SF and JP were responsible for reference screening, quality assessment, data extraction and management. AMR, MT and SB gave advice throughout the project and contributed to data interpretation. AX and SF were responsible for the statistical inferences and qualitative synthesis respectively. SF and AX led the development of the first draft of the manuscript. SB, MT and AMR gave editorial comments on the draft manuscript. AX is the guarantor of the review. AX, SF, JP, SB, MT, and AMR read and approved the manuscript.

For peer review only

REFERENCES

1 Institute of Medicine. To err is human New York: National Academies Press; 2001.

2 Wanless D. Securing our Future Health: Taking a Long-Term View London: HM Treasury; 2002.

3 NHS England. Transforming Participation in Health and Care: Guidance to support commissioners. London; 2013.

4 NHS England. Five Year Forward View. London; 2015.

5 Wright S, Walmsley E, Harvey S, Robinson E, Ferrando-Vivas P, Harrison D, et al. Family-Reported Experiences Evaluation (FREE) study: a mixed-methods study to evaluate families' satisfaction with adult critical care services in the NHS. Health Services and Delivery Research. 2015; 3: p. 45.

6 Azoulay E, Pochard F, Kentish-Barnes N, Chevret S, Aboab J, Adrie C, et al. Risk of post-traumatic stress symptoms in family members of intensive care unit patients. American Journal of Respiratory and Critical Care Medicine. 2005;: p. 987-994.

7 Azoulay E, Chaize M, Kentish-Barnes N. Involvement of ICU families in decisions: fine-tuning the partnership. Annals of Intensive Care. 2014; 4: p. 1.

8 Bench S, Cornish J, Xyrichis A. A qualitative exploration of Intensive Care discharge summaries for General Practice staff. British Journal of General Practice. 2016; 66(653): p. e904-e912.

9 Davidson JE, Powers K, Hedayat KM, Tieszen M, Kon AA, Shepard E, et al. Clinical practice guidelines for support of the family in the patient-centered intensive care unit: American College of Critical Care Medicine Task Force 2004–2005. Critical Care Medicine. 2007; 35(2): p. 605-622.

10 Olding M, McMillan SE, Reeves S, Schmitt MH, Puntillo K, & Kitto S. Patient and family involvement in adult critical and intensive care settings: a scoping review. Health Expectations. 2015; 19: p. 1183-1202.

11 Liput SA, Kane-Gill SL, Seybert AL, Smithburger PL. A Review of the Perceptions of Healthcare Providers and Family Members Toward Family Involvement in Active Adult Patient Care in the ICU. Critical Care Medicine. 2016; 44(6): p. 1191-1197.

12 Reeves S, Fletcher S, Barr H, Birch I, Boet S, Davies N, et al. A BEME systematic review of the effects of interprofessional education: BEME Guide No. 39. Medical Teacher. 2016; 38(7): p. 656-668.

13 Xyrichis A, Fletcher S, Brearley S, Philippou J, Purssell E, Terblanche M, Rafferty AM, Reeves S. Interventions to promote patients and families' involvement in adult intensive care settings: a protocol for a mixed-method systematic review. Systematic Reviews. 2019; 8(1): p. 185-192.

14 Dufour É, Duhoux A, Bolduc J. Measured and perceived effects of audit and feedback on nursing performance: a mixed methods systematic review protocol. Systematic Reviews. 2019; 8(1): p. 38.

15 Carroll C, Booth A, Leaviss J, Rick J. "Best fit" framework synthesis: refining the method. Medical Research Methodology. 2013; 13(1): p. 37.

For peer review only

16 Choi YS, Bosch SJ. Environmental affordances: Designing for family presence and involvement in patient care. Health Environments Research & Design Journal. 2013; 6(4): p. 53-75.

17 Rippin AS, Zimring C, Samuels O, Denham ME. Finding a Middle Ground: Exploring the Impact of Patient-and Family-Centered Design on Nurse–Family Interactions in the Neuro ICU. Health Environments Research & Design Journal. 2015; 9(1): p. 80-98.

18 Dalal AK, Dykes PC, Collins S, Lehmann LS, Ohashi K, Rozenblum R, et al. A web-based, patient-centered toolkit to engage patients and caregivers in the acute care setting: a preliminary evaluation. Journal of the American Medical Informatics Association. 2015; 23(1): p. 80-87.

19 Dykes PC, Rozenblum R, Dalal A, Massaro A, Chang F, Clements M, et al. Prospective evaluation of a multifaceted intervention to improve outcomes in intensive care: the Promoting Respect and Ongoing Safety Through Patient Engagement Communication and Technology Study. Critical Care Medicine. 2017; 45(8): p. e806-e813.

20 Ernecoff NC, Witteman HO, Chon K, Buddadhumaruk P, Chiarchiaro J, Shotsberger KJ, et al. Key stakeholders' perceptions of the acceptability and usefulness of a tablet-based tool to improve communication and shared decision making in ICUs. Journal of Critical Care. 2016; 33: p. 19-25.

21 Huffines M, Johnson KL, Naranjo LLS, Lissauer ME, Fishel MAM, Howes SMA, et al. Improving family satisfaction and participation in decision making in an intensive care unit. Critical Care Nurse. 2013; 33(5): p. 56-69.

22 Almoosa KF, Goldenhar LM, Panos RJ. Characteristics of discussions on cardiopulmonary resuscitation between physicians and surrogates of critically ill patients. Journal of Critical Care. 2009; 24(2): p. 280-287.

23 Garrouste-Orgeas M, Max A, Lerin T, Grégoire C, Ruckly S, Kloeckner M, et al. Impact of proactive nurse participation in ICU family conferences: a mixed-method study. Critical Care Medicine. 2016; 44(6): p. 1116-1128.

24 Hollman Frisman G, Wåhlin I, Orvelius L, Ågren S. Health-promoting conversations - a novel approach to families experiencing critical illness in the ICU environment. Journal of Clinical Nursing. 2018; 27(3): p. 631-639.

25 Huang KB, Weber U, Johnson J, Anderson N, Knies AK, Nhundu B, et al. Primary Care Physician Involvement in Shared Decision Making for Critically Ill Patients and Family Satisfaction with Care. The Journal of the American Board of Family Medicine. 2018; 31(1): p. 64-72.

26 Curtis JR, Treece PD, Nielsen EL, Gold J, Ciechanowski PS, Shannon SE, et al. Randomized trial of communication facilitators to reduce family distress and intensity of end-of-life care. American Journal of Respiratory and Critical Care Medicine. 2016; 193(2): p. 154-162.

27 Shaw DJ, Davidson JE, Smilde RI, Sondoozi T, Agan D. Multidisciplinary team training to enhance family communication in the ICU. Critical Care Medicine. 2014; 42(2): p. 265-271.

For peer review only - http://bmjopen.bmj.com/site/about/guidelines.xhtml

28 Cray L. A collaborative project: initiating a family intervention program in a medical intensive care unit. Focus on Critical Care. 1989; 16(3): p. 213-218.

29 Davidson JE, Daly BJ, Agan D, Brady NR, Higgins PA. Facilitated sensemaking: a feasibility study for the provision of a family support program in the intensive care unit. Critical Care Nursing Quarterly. 2010; 33(2): p. 177-189.

30 Marshall AP, Wake E, Weisbrodt L, Dhaliwal R, Spencer A, Heyland DK. A multi-faceted, family-centred nutrition intervention to optimise nutrition intake of critically ill patients: The OPTICS feasibility study. Australian Critical Care. 2016; 29(2): p. 68-76.

31 White DB, Angus DC, Shields AM, Buddadhumaruk P, Pidro C, Paner C, et al. A Randomized Trial of a Family-Support Intervention in Intensive Care Units. New England Journal of Medicine. 2018; 378(25): p. 2365-2375.

32 Allen SR, Pascual J, Martin N, Reilly P, Luckianow G, Datner E, et al. A novel method of optimizing patient-and family-centered care in the ICU. Journal of Trauma and Acute Care Surgery. 2017; 82(3): p. 582-586.

33 Jacobowski NL, Girard TD, Mulder JA, Ely EW. Communication in critical care: family rounds in the intensive care unit. American Journal of Critical Care. 2010; 19(5): p. 421-430.

34 Weber U, Johnson J, Anderson N, Knies AK, Nhundu B, Bautista C, et al. Dedicated Afternoon Rounds for ICU Patients' Families and Family Satisfaction With Care. Critical Care Medicine. 2018; 46(4): p. 602-611.

35 Prichard C, Newcomb P. Benefit to family members of delivering hand massage with essential oils to critically ill patients. American Journal of Critical Care. 2015; 24(5): p. 446-449.

36 Davidson JE, Powers K, Hedayat KM, Tieszen M, Kon AA, Shepard E, Spuhler V, Todres ID, Levy M, Barr J, Ghandi R. Clinical practice guidelines for support of the family in the patient-centered intensive care unit: American College of Critical Care Medicine Task Force 2004–2005. Critical care medicine. 2007 Feb 1;35(2):605-22.

37 Davidson JE, Aslakson RA, Long AC, Puntillo KA, Kross EK, Hart J, Cox CE, Wunsch H, Wickline MA, Nunnally ME, Netzer G. Guidelines for family-centered care in the neonatal, pediatric, and adult ICU. Critical care medicine. 2017 Jan 1;45(1):103-28.

38 Patient- and Family-Centered Care Initiatives in Acute Care Settings: A Review of the Clinical Evidence, Safety and Guidelines [Internet]. Ottawa, Canada: Canadian Agency for Drugs and Technologies in Health; 2015. http://www.ncbi.nlm.nih.gov/books/NBK350000/. Accessed September 14, 2016.

39 Singer AE, Ash T, Ochotorena C, Lorenz KA, Chong K, Shreve ST, Ahluwalia SC. A systematic review of family meeting tools in palliative and intensive care settings. American Journal of Hospice and Palliative Medicine®. 2016 Sep;33(8):797-806.

40 Bibas L, Peretz-Larochelle M, Adhikari NK, Goldfarb MJ, Luk A, Englesakis M, Detsky ME, Lawler PR. Association of surrogate decision-making interventions for critically ill adults with patient, family, and resource use outcomes: a systematic review and meta-analysis. JAMA network open. 2019 Jul 3;2(7):e197229.

For peer review only - http://bmjopen.bmj.com/site/about/guidelines.xhtml

41 Rose SC, Bisson J, Churchill R, Wessely S. Psychological debriefing for preventing post traumatic stress disorder (PTSD). Cochrane database of systematic reviews. 2002(2).

For peer review only

For peer review only - http://bmjopen.bmj.com/site/about/guidelines.xhtml

List of Tables

Table 1: Included studies 19
Table 2: Intervention characteristics..21
Table 3: Quality assessment - Quantitative studies 28
Table 4: Quality assessment - Qualitative studies..28
Table 5: Communication outcome measures 29
Table 6: Decision making outcome measures..29
Table 7: Satisfaction outcome measures 30
Table 8: Anxiety outcome measures..31
Table 9: Depression outcome measures..31
Table 10: Trauma outcome measures..32
Table 11: Patient outcome measures 32

For peer review only

Table 1: Included studies

Author	Year	Country and setting	Design	Sample size	Research approach	Intervention
Allen et al	2017	USA – Surgical ICU	Non-randomised, before and after study	Patients: n=847<br>Family members: n=429	Quantitative	Engaging family members on rounds
Almoosa et al	2009	USA – Medical ICU	Prospective, 2-centre observational study	Patients: n=85<br>Family members: n=85	Quantitative	CPR Discussions
Choi and Bosch	2013	USA – Neuro ICU and Trauma ICU	Comparative observational study	Patients: n=81<br>Family members: n=81	Quantitative	Patient and family centred unit design
Cray	1989	USA – Medical ICU	Post hoc evaluation study	Patients: n=76<br>Family members: n=76	Quantitative	Family intervention programme
Dalal et al	2015	USA – Medical ICU (Oncology Unit)	Post hoc evaluation study	Patients: n=26<br>Family members: n=77	Quantitative	Patient centred toolkit
Davidson et al	2010	USA – Mixed ICU	Feasibility study		Quantitative	Family Support Programme, based on Facilitated Sensemaking
Dykes et al	2017	USA – Medical ICU	Non-randomised, before and after study	Patients: n=58<br>Family members: n=156	Quantitative	Patient Engagement Communication and Technology (PROSPECT)
Ernecoff et al	2016	USA – Medical ICU	Qualitative interview study	Family members: n=30<br>Staff members: n=28	Qualitative	Tablet-based support tool
Garrouste-Orgeas et al	2016	France – Surgical ICU	Randomised controlled trial with nested qualitative study	Patients: n=100<br>Family members: n=88	Mixed methods	Proactive participation of a nurse in family conferences
Hollman Frisman et al	2018	Sweden – ICU	Qualitative interview study	Patients: n=8<br>Family members: n=10	Qualitative	Health promoting conversations
Huang et al	2018	USA – Neuroscience ICU	Prospective, single-centred observational study	Family members: n=263	Quantitative	Primary care physician involvement in decision-making in the ICU
Huffines et al	2013	USA – Surgical ICU	Non-randomised, before and after study	Family members: n=48	Quantitative	SICU Family supportive care algorithm
Marshall et al	2016	Australia – General ICU	Feasibility study	Family members: n=51	Qualitative	Multi-faceted family-centred nutrition intervention
Jacobowski et al	2010	USA – Medical ICU	Non-randomised, before and after study	Family members: n=111	Quantitative	Family rounds
Prichard and Newcomb	2015	USA – Trauma ICU	Quasi experimental pilot study	Family members: n=30	Quantitative	Hand massage
Randall-Curtis et al	2016	USA – General ICU	Randomised controlled trial	Patients: n=168<br>Family members: n=268	Quantitative	Communication facilitator

For peer review only

For peer review only - http://bmjopen.bmj.com/site/about/guidelines.xhtml

Author	Year	Country and setting	Design	Sample size	Research approach	Intervention
Rippin et al	2015	USA – Neuroscience ICU	Comparative observational study	Family members: n=54<br>Staff members: n=18	Qualitative	Family centred unit design
Shaw et al	2014	USA – General ICU	Non-randomised, before and after study	Patients: n=121<br>Family members: n=121	Quantitative	Multidisciplinary team training to enhance family communication in the ICU
Weber et al	2018	USA – Neuroscience ICU	Non-randomised, before and after implementation study	Family members: n=141	Quantitative	Family rounds
White et al	2018	USA - 2 Neuro ICU, 2 Mixed ICU, 1 Medical ICU	Multicentre, stepped-wedge, cluster-randomized	Patients: n= 1420<br>Family members: n= 1106	Quantitative	Multicomponent family-support intervention

For peer review only

Table 2: Intervention characteristics

Author	Activity Type	Intervention	Purpose	Procedure	Participants	Beneficiary	Outcomes
Allen et al. 2017	Rounds based	Family member involvement in rounds	Engaging and integrating ICU patients' family members and surrogates on daily rounds	On admission to the ICU, a family member was invited to participate on daily rounds with the critical care team.	Family members, nurses, physicians	Family members, nurses, physicians	• significantly increased family member knowledge<br>• strengthened relationship between family members and doctors<br>• nurses reported: greater enjoyment<br>• improved communication<br>• reduction in workload<br>• increased support for patient and family centered care.<br>• increased physician satisfaction post intervention
Almoosa et al. 2009	Discussion based	CPR Discussions	The implementation of structured, informed CPR specific conversations with family members and surrogates from relevant physicians	Physician initiated CPR conversation about: chest compressions, electrical cardioversion, mechanical ventilation	Physicians, residents, fellows or family	Family members	• satisfaction with CPR discussions was higher after the intervention
Choi and Bosch 2013	Unit design	Patient and family centred unit design	Unit redesign undertaken to enable physical space to more broadly facilitate family interaction with patient and family interaction with staff	The intervention unit included larger spaces and more comfortable accommodations for visitors	Family members	Family members and patients	• increased family interactions with patient<br>• increased family interactions with staff
Cray et al. 1989	Multi-component	Family intervention programme	Multi-component intervention to enhance family involvement in and understanding of their loved one's condition in ICU	Intervention components included: Family conference, Telephone conversation, Visit by nurse, Follow up visit to the intermediary care unit	Family members, Clinical nurse specialists	Family members	• strong agreement that the intervention helped family members to understand their loved one's illness and benefit other families.<br>• high satisfaction with intervention components.

For peer review only - http://bmjopen.bmj.com/site/about/guidelines.xhtml

Author	Activity Type	Intervention	Purpose	Procedure	Participants	Beneficiary	Outcomes
Dalal et al. 2016	Web based/ electronic	Patient centred toolkit (PCTK)	The PCTK was a suite of web-based patient-facing and provider-facing tools designed to facilitate collaborative decision making by providing access to tailored educational content and facilitating patient-provider communication	The PCTK was accessible by patients, caregivers, and providers from any web-enabled device connected to the hospital's secure intranet.	Patients and caregivers	Patients and family members	• the majority of patients and caregivers surveyed were satisfied or extremely satisfied with the intervention
Davidson et al. 2010	Multi-component	Family Support Programme, based on Facilitated Sensemaking	A systems-based patient-centered care and engagement program	The intervention consisted of 2 main components: personalized instruction and provision of family visiting kits.	Family members, Clinical nurse specialists	Family members	• high rate of instrument reliability<br>• a breakdown of the top ten needs of family members<br>• a ranking of interventions' helpfulness<br>• identification of any additional needs
Dykes et al. 2017	Web based/ electronic	Promoting Respect and Ongoing Safety through Patient Engagement Communication and Technology (PROSPECT).	A systems-based patient-centered care and engagement program designed to enhance patient and care partner experience in the ICU	60 minute training session to facilitate patient-centred care in healthcare professionals,<br>A web based – toolkit to apply the training,<br>Delivery of the Patient SatisfActive Model from nurses to patients in ICU	Nurses and patients	Nurses and patients	• relative reduction in the rate of adverse effects<br>• improvements in patient satisfaction<br>• improvements in care partner satisfaction

For peer review only - http://bmjopen.bmj.com/site/about/guidelines.xhtml

Author	Activity Type	Intervention	Purpose	Procedure	Participants	Beneficiary	Outcomes
Ernecoff et al. 2016	Web based/ electronic	Tablet-based and video-driven communication and decision support tool	A tool was conceptualised to: 1) prepare the family for conversations with clinicians, 2)give clinicians tailored information about the family and patient in advance of the family meeting, 3) promote a personalized relationship between clinician and family, and 4) provide general decision support to surrogates.	The sections of the tool for surrogates were: 1) orienting surrogates to the ICU, 2) explaining principles of surrogate decision making 3) providing a question prompt list and opportunity to write down questions, 4) a values clarification exercise 5) education about treatment pathways (e.g., life prolonging treatment, comfort-focused treatment; and a time-limited trial of ICU care);6) eliciting surrogates prognostic information, and 7) providing psycho-social resources	Family members (surrogates)	Family members	• enhancing and supplementing communication between surrogates and the clinical team<br>• leveraging surrogates' downtime before and between clinician-family meetings<br>• helping surrogates to consider the patient's values and treatment options<br>• allowing for repetition and review of information
Garrouste-Orgeas et al. 2016	Discussion based	Proactive participation of a nurse in family conferences	Integration of nursing staff in family conferences focussed around: Naming emotions, expressing Understanding, showing Respect, articulating Support for the patient, and Exploring the family's emotional state	All family members who wished to attend were escorted to the room. Briefly, an open question was asked first to encourage the family members to express themselves. A substantial proportion of the time was devoted to listening to the family, and the professionals used simple words to enhance comprehension. At the end of the conference, the family was allowed enough time to ask all the questions they had.	Physicians, nurses, family members	Family members	• family members reported that the conferences allowed them to receive and assimilate information<br>• be listened to regarding both their positive and negative feelings<br>• receive compassion and respect

For peer review only

Author	Activity Type	Intervention	Purpose	Procedure	Participants	Beneficiary	Outcomes
Hollman Frisman et al. 2018	Discussion based	(Nurse led) Health promoting conversations with families	The aim of the conversations was to create a context for change related to the families' problems and resources.	Discussion of the aim of the conversation series and the families' and nurses' expectations about the conversations and each other's roles. The three conversation sessions focused on topics that the families considered important, and the dialogue and questioning intended to identify resources within and outside the family. At the end of each conversation, the nurse offered a short reflection on how the family members had experienced the session. A closing letter was sent to the family 2–3 weeks after the last conversation to summarise further possibilities for reflection.	Nurses and family members	Family members	• health promoting conversations led to increasing emotional openness<br>• enhanced consciousness regarding illness<br>• greater family member satisfaction<br>• a valuable sense of confirmation<br>• general promotion of family wellbeing
Huang et al. 2018	Discussion based	Primary Care Physician involvement in decision-making in the ICU	The study evaluated survey results which measured family member satisfaction with general ICU care and shared decision making between Primary Care Physicians and family members	Examined involvement of the patient's Primary Care Physician and shared decision making.	Primary care physicians	Family members	• a higher proportion reporting PCP involvement felt completely satisfied with their inclusion in the ICU decision making process

For peer review only

Author	Activity Type	Intervention	Purpose	Procedure	Participants	Beneficiary	Outcomes
Huffines et al. 2013	Web based/ electronic	SICU Family Supportive Care Algorithm	The goal of the interventions in the 24-hour bundle is to inform families about the importance of their participation in decision making and to inform them of the resources available to help them participate in decision making.	Within 24 hours of admission, a member of the intensivist team meets with the family. Also within the first 24 hours after a patient's SICU admission, the patient's family is encouraged to watch an on-demand 10-minute video.<br>72 hours:<br>Reaffirm and ensure family is supported. Encouraged to participate in decision making in rounds.<br>96 hours:<br>Implement a family meeting with the multidisciplinary team.	Bedside nurses	Family members	• mixed to positive outcomes when measuring family satisfaction<br>• staff teamwork<br>• participation in decision making<br>• frequency of support
Marshall et al. 2016	Multi-component	Multi-faceted, family-centred nutrition intervention	To assist families in discussing nutritional goals with health professionals	Consisted of:<br>Family interviews,<br>Focussed education sessions, and Nutrition diaries	Dieticians, Family Nurses	Families and patients	• perceptions around how nutrition education can be improved<br>• the diversity of experience relating to the provision of in-hospital nutrition therapy<br>• continuity of existing family member involvement approaches<br>• the importance of families as advocates
Jacobowski et al. 2010	Rounds based	Family rounds	To enhance communication between medical and nursing staff and the families of ICU patients	The attending physician provided a summary for the family using understandable, lay language and the family was offered an opportunity to ask questions of the team	Physicians, nurses, family members	Physicians, nurses, family members	• communication regarding condition improved significantly as did decision making support.<br>• there was a decline in the number of family members who thought that they had sufficient time to address their questions and concerns.

For peer review only - http://bmjopen.bmj.com/site/about/guidelines.xhtml

Author	Activity Type	Intervention	Purpose	Procedure	Participants	Beneficiary	Outcomes
Prichard and Newcomb 2015	Physical	Hand massage	To provide physical relief to patients in the ICU through hand massage	Participants were taught to administer hand massage in compliance with the M Technique, a registered method of simple, structured touch that has been used on critically ill patients with positive effects. For this study, the technique was used on hands. Participants applied the intervention twice daily for 5 minutes per session for 3 consecutive days	Family members, patients	Family members, patients	• anxiety was greatly reduced in the treatment group
Randall-Curtis et al. 2016	Discussion based	Communication-facilitator	To understand the family's concerns, needs, and communication characteristics	Consisted of facilitated: Interviews, Meetings, Communication and emotional support; as well as Facilitator participation in family conferences and 24 hour follow up	Nurse or social worker trained to improve communicatio n between the ICU team and the family by acting as a communicatio n facilitator or navigator	Family members	• adjusted depression scores were lowered alongside ICU costs
Rippin et al. 2015	Unit design	Family Centred Unit design	Unit designed to prioritise and engage the family in the ICU context	The FCU physically integrated family into the fabric of the unit. Nurses worked between centralized nursing stations and decentralized alcoves just outside patient rooms for improved monitoring and safety. Rooms were larger (245 sq ft) with more space around the bedside.	Nurses and family members	Family members	• reduction in problematic bedside co-presence (or overcrowding)<br>• a continuity of pre intervention distribution patterns in which nurse and family remained clustered in their respective 'domains'<br>• nurse perspectives which acknowledged the unpredictability and complexity of the ICU and the subsequent mixed results which this newly implemented unit design encouraged

For peer review only - http://bmjopen.bmj.com/site/about/guidelines.xhtml

Author	Activity Type	Intervention	Purpose	Procedure	Participants	Beneficiary	Outcomes
Shaw et al. 2014	Discussion based	Multidisciplinary team training to enhance family communication in the ICU	The training material was selected to improve communication. The training was also designed to foster team building and improve collaborative relationships among clinicians from multiple disciplines.	Training included how to conduct and participate in patient/family conferences, addressing goals of care and giving critical information in the intensive care setting so that all caregivers might speak to patients and their families with a common voice. The training was designed to address known drivers of family satisfaction, as well as to address each of the 21 items being measured in the staff confidence survey.	ICU team members	MDT team members and family members	• staff confidence and family satisfaction were shown to significantly improve
Weber et al. 2018	Rounds based	Family Rounds	To improve family satisfaction in ICU experience by integrating them into rounds processes	After each session, the nursing leader recorded (1) whether family rounds occurred (2), how many families participated, and (3) how many patients the NICU team had rounded on that morning	Nursing staff	Family members	• family reported improved satisfaction with decision making, frequency of communication, receiving emotional support and with coordination of care<br>• percentage improvements in satisfaction scores were not large enough to reach statistical significance
White et al. 2018	Multi-component	Multicomponent family-support intervention	Collaborative intervention between nursing staff and family members designed to support caregivers in the ICU	Nurses received advanced communication training. A family support pathway was instituted. Intensive support for implementation was provided to each ICU by a quality-improvement specialist to incorporate the family-support pathway into clinicians' workflow.	Nursing staff, ICU team, family members	Family members and nursing staff	• significant improvements were noted with family satisfaction regarding:<br>• the quality of communication with clinicians, and<br>• perceptions of patient centeredness<br>• the intervention led to an average reduction in the length of stay in the ICU of 3 days

For peer review only - http://bmjopen.bmj.com/site/about/guidelines.xhtml

Table 3: Quality assessment - Quantitative studies

Study Id	Aim of Evaluation	Research Design	Sample size	Power calculation	Number of groups	Method of allocation	Allocation concealment	Blinding	Loss to follow up	Data collection method	Significance measures	Data analysis method	Reported biases or confounders	Relevance	Ethics	Strength of design	Strength of numbers	Quality of information	Quality of study	Overall weighting
Allen 2017	+	-	+	-	-	-	-	-	-	-	+	+	+	+	+	?	?	?	?	Low
Almoosa 2009	+	+	+	+	+	-	-	-	+	+	+	+	+	+	+	?	?	?	?	Moderate
Choi 2013	+	-	+	-	+	-	-	-	-	-	+	+	+	+	-	?	?	?	?	Low
Cray 1989	+	-	?	?	?	?	?	?	?	?	+	+	-	+	?	-	+	?	-	Low
Dalal 2016	-	-	+	-	-	-	-	-	-	-	+	+	+	+	+	?	?	?	?	Very low
Davidson 2010	+	+	+	-	+	-	-	-	+	+	+	+	+	+	+	?	?	?	?	Moderate
Dykes 2017	+	-	+	+	-	-	-	-	-	+	+	+	+	+	+	?	?	?	?	Low
Garrouste-Orgeas 2016	+	+	+	+	+	+	+	-	+	+	+	+	+	+	+	+	+	+	+	Very high
Huang 2018	+	+	+	+	+	-	-	-	-	+	+	+	+	+	+	-	+	+	+	Moderate
Huffines 2013	+	+	+	-	+	-	-	-	+	+	+	+	+	+	?	?	?	?	?	Moderate
Jacobowski 2010	+	?	+	?	+	?	?	?	?	+	+	+	+	+	+	?	+	?	?	Moderate
Marshall 2016	+	+	+	-	?	-	-	-	-	+	+	+	+	+	+	+	+	?	+	Moderate
Prichard 2015	+	?	+	?	+	-	?	?	+	+	+	+	+	+	+	?	+	+	?	High
Randall-Curtis 2016	+	+	+	+	+	+	+	?	-	+	+	+	+	+	+	+	+	+	+	Very high
Shaw 2014	+	?	+	+	+	?	?	?	-	+	+	+	+	+	+	?	+	+	?	High
Weber 2018	+	+	+	+	+	?	?	?	?	+	+	+	+	+	+	?	?	?	?	High
White 2018	+	+	+	+	+	+	+	?	+	+	+	+	+	+	+	+	+	+	+	Very high

+ Low concern, - High concern, ? Unclear. Quality assessment tool elaborated in Xyrichis et al.[13]

Table 4: Quality assessment - Qualitative studies

Study Id	Aim of Evaluation	Sampling	Data collection	Data analysis	Research relations	Findings	Transferability	Relevance & Usefulness	Ethics	Quality of information	Quality of study	Overall weighting
Ernecoff 2016	+	+	+	+	-	+	+	+	+	+	+	High
Frisman 2018	+	+	+	+	-	+	+	+	+	+	+	High
Garrouste-Orgeas 2016	+	+	+	+	-	+	+	+	+	+	+	High
Marshall 2016	+	+	+	+	-	+	?	+	+	?	?	Moderate
Rippin 2015	+	?	+	+	?	+	+	+	-	?	?	Low

+ Low concern, - High concern, ? Unclear. Quality assessment tool elaborated in Xyrichis et al.[13]

For peer review only - http://bmjopen.bmj.com/site/about/guidelines.xhtml

Table 5: Communication outcome measures

Study / Intervention / Measure	Jacobowski et al. 2010	Weber et al. 2018	Shaw et al. 2014	White et al. 2018	Allen et al. 2017
**Intervention**	**Family rounds**	**Family rounds**	**Team training**	**Multi family support**	**Family on rounds**
**FS ICU 24 – Frequency of nurse communication**	% highest score<br>Pre: 64%<br>Post: 57%<br>**Change: -7% p=0.30**<br>**RR: 0.89 CI: 0.70-1.12**	% top scores<br>Control: 62%<br>Intervention: 78%<br>**Change: 16% p>0.05**<br>**RR: 1.24 CI 0.98-1.58**	Mean score<br>Pre: 79.2<br>Post: 87.18<br>**Change: 7.98 p=0.04***		
**FS ICU 24 – Frequency of doctor communication**	% highest score<br>Pre: 38%<br>Post: 60%<br>**Change: 22% p=0.004***<br>**RR: 1.60 CI 1.18-2.17**	% top scores<br>Control: 43%<br>Intervention: 56%<br>**Change: 13% p>0.05**<br>**RR: 1.31 CI 0.90-1.89**	Mean score<br>Pre: 67.86<br>Post: 76.69<br>**Change: 8.83 p=0.04***		
**FS ICU 24 – Honesty of information**			Mean score<br>Pre: 77.78<br>Post: 87.08<br>**Change: 9.30 p=0.01***		
**Quality of Communication (QOC)**				Mean score<br>Control: 62.7<br>Intervention: 69.1<br>**Change: 6.39 p=0.001***<br>**CI 2.57 – 10.20**	
**Communication improvement**					Pre: n=49 (100%)<br>Post: n=47 (100%)<br>**Change 0% p=0.68**<br>**RR: 1, CI: 0-0**
**Overall metric for comparison**	**RR: 1.60**	**RR: 1.31**	**MD: 8.83**	**MD: 6.39**	**RR: 1**

Table 6: Decision making outcome measures

Study / Intervention / Measure	Huang et al. 2018	Shaw et al. 2014	Weber et al. 2018	Jacobowski et al. 2010	Huffines et al. 2013	Almoosa et al. 2009
**Intervention**	**Primary physician involvement**	**Team training**	**Family rounds**	**Family rounds**	**Support care algorithm**	**CPR discussions**
**FS ICU 24 – Decision making**	Mean (sd)<br>Control: 80.07 (12.76)<br>Intervention: 81.06 (15.1)<br>**Change: 0.99 p=0.16**<br>**CI -3.00 – 4.98**	Mean score<br>Pre: 77.47<br>Post: 83.32<br>**Change: 5.85 p=0.05***	Mean score (SD)<br>Control: 85.1 (16.3)<br>Intervention: 88.6 (14.6)<br>**Change: 3.5 p=0.20**<br>**CI: -1.98-8.98**			
**Inclusion in decision making**	% completely satisfied<br>Control: 61.4%<br>Intervention: 75.9%<br>**Change: 14.5% p=0.05***<br>**RR: 1.23 CI 1.03-1.49**			% highest score<br>Pre: 66%<br>Post: 76%<br>**Change: 10% p=0.12**<br>**RR: 1.15 CI 0.96-1.38**		
**Control over patient care**	% completely satisfied<br>Control: 55.6%<br>Intervention: 73.6%<br>**Change: 18% p=0.02***<br>**RR: 1.31 CI 1.07-1.61**					
**Support in decision making**				% highest score<br>Pre: 49%<br>Post: 69%<br>**Change: 20% p=0.005***		

For peer review only

For peer review only - http://bmjopen.bmj.com/site/about/guidelines.xhtml

				RR: 1.39 CI 1.09-1.79		
**Share in decisions about care planning**					% scoring excellent<br>Pre: 45%<br>Post: 68%<br>**Change: 23% p=0.009***<br>**RR: 1.52 CI 1.03-2.24**	
**Time to decision**						Days mean (sd)<br>Control 7.4 (3.2)<br>Interv. 6.6 (6.5)<br>**Change -0.8 days CI -1.48-3.08**
**Overall metric for comparison**	**RR: 1.31**	**MD: 5.85**	**MD: 3.5**	**RR: 1.39**	**RR: 1.52**	**MD: -0.8**

Table 7: Satisfaction outcome measures

**Study**	**Huang et al. 2018**	**Shaw et al. 2014**	**Dykes et al. 2017**	**Weber et al. 2018**	**Huffines et al. 2013**
**Intervention / Measure**	**Primary physician involvement**	**Staff teamwork training**	**ICT-based engagement**	**Family rounds**	**Supportive care algorithm**
**FS ICU 24 – Global score**	Mean (sd)<br>Control: 84.91 (12.17)<br>Intervention: 86.4 (11.76)<br>**Change: 1.49 p=0.16**<br>**CI -2.14 - 5.12**	Mean score<br>Pre: 83.21<br>Post: 85.69<br>**Change: 2.48 p=0.32**	Mean score<br>Pre: 84.3 (3)<br>Post: 90 (1.9)<br>**Change: 5.7 p<0.05***<br>**CI 2.31-9.09**	Mean score (SD)<br>Control: 86.0 (16.0)<br>Intervention: 90.8 (10.7)<br>**Change: 4.8 p=0.20**<br>**CI: -0.12 - 9.72**	
**HCAHPS**			% top score 9-10<br>Pre: 71.8%<br>Post: 93.3%<br>**Change 21.5 P<0.05***<br>**RR 1.33 CI 1.10-1.55**		
**Support given**				% top scores<br>Control: 54%<br>Intervention: 71%<br>**Change: 17% p>0.05**<br>**RR: 1.32 CI 0.99-1.75**	% scoring excellent<br>Pre: 60%<br>Post 75%<br>**Change: 15% p=0.14**<br>**RR: 1.23 CI 0.91-1.65**
**Overall metric for comparison**	**MD: 1.49**	**MD: 2.48**	**RR: 1.33**	**RR: 1.32**	**RR: 1.23**

**Study**	**White et al. 2018**	**Cray 1989**	**Dalal et al. 2015**	**Jacobowski et al. 2010**
**Intervention / Measure**	**Multi-component family support programme**	**Family support programme**	**Patient centred toolkit**	**Family rounds**
**PPPC**	Mean score<br>Control: 1.8<br>Intervention: 1.7<br>**Change: -0.15 p=0.006***<br>**CI –0.26 - –0.04**			
**Satisfaction with intervention**		% Satisfied<br>**100% agreed (76/76)**	% Satisfied<br>**72% (13/18)**	
**Time to ask questions**				% highest score<br>Pre: 40%<br>Post: 23%<br>**Change: -17% p=0.02***<br>**RR: 0.57 CI 0.37-0.90**
**Overall metric for comparison**	**MD: 0.15**	**100%**	**72%**	**RR: 0.57**

Table 8: Anxiety outcome measures

Study / Intervention / Measure	Garrouste et al. 2016	Prichard & Newcomb 2015	White et al. 2018	Randall-Curtis et al. 2016
**Intervention**	**IP family conference**	**Hand massage**	**Multi family support**	**Communication facilitator**
**HADS - Anxiety**	90 days, Median [IQR]<br>Control: 8 [4.5-12]<br>Intervention: 4 [1-9]<br>**Change: -4 p=0.01***<br><br>% w\ Anxiety score > 8<br>Control: 52.3% (n=23)<br>Intervention: 33.3% (n=14)<br>**Change: 19% p=0.08**<br>**RR: 0.95, CI 0.63-1.44**	Mean change in score<br>Control: -0.4<br>Intervention: -3.87<br>**Change: -3.47 p=0.002***<br>**CI -5.5 - -1.4**		
**HADS – Global score**			Mean score<br>Control: 12.0<br>Intervention: 11.7<br>**Change: -0.34 p=0.61**<br>**CI –1.67 – 0.99**	
**GAD 7 - Anxiety**				Mean score 3 months<br>Control: 3.0<br>Intervention: 2.3<br>**Change: -0.7 p=0.50**<br>**CI: -2.91 - 1.42**<br><br>Mean score 6 months<br>Control: 2.7<br>Intervention: 1.8<br>**Change: -0.9 p=0.43**<br>**CI: -3.10 - 1.32**
**Overall metric for comparison**	**MD: -4**	**MD: -3.47**	**MD: -0.34**	**MD: -0.7**

Table 9: Depression outcome measures

Study / Intervention / Measure	Garrouste et al. 2016	Prichard & Newcomb 2015	Curtis et al. 2016
**Intervention**	**IP family conference**	**Hand massage**	**Communication facilitator**
**HADS – Depression**	At 90 days, Median [IQR]<br>Control: 5.5 [1-11.5]<br>Intervention: 2 [0-6]<br>**Change: -3.5 p=0.04***<br><br>% sign. Depress. score > 8<br>Control: 38.6% (n=17)<br>Intervention: 23.8% (n=10)<br>**Change: 14.8% p=0.14**<br>**RR: 0.61, CI 0.31-1.18**	Mean change in score<br>Control: -0.3<br>Intervention: -2.5<br>**Change: -2.2 p=0.10**<br>**CI -0.49 - 4.7**	
**PHQ 9 Depression**			Mean score 3 months<br>Control: 4.9<br>Intervention: 3.1<br>**Change: -1.8 p=0.09**<br>**CI: -3.89 - 0.31**<br><br>Mean score 6 months<br>Control: 4.7<br>Intervention: 2.4<br>**Change: -2.3 p=0.01***<br>**CI: -4.30 - -0.42**
**Overall metric for comparison**	**MD: -3.5**	**MD: -2.2**	**MD: -1.8**

For peer review only

Table 10: Trauma outcome measures

Study	Garrouste et al. 2016	Curtis et al. 2016	White et al. 2018
Intervention / Measure	**Interprofessional family conference**	**Communication facilitator**	**Multicomponent family support**
**PDEQ**	Median [IQR]<br>Control: 14.5 [11-23]<br>Intervention: 13 [0-17]<br>**Change: -1.5 p=0.17**		
**IES-R**	At 90 days, Median [IQR]<br>Control: 24 [12.5-45]<br>Intervention: 21 [9-33]<br>**Change: -3 p=0.24**		
**IES - PTSD (0-88)**		Mean score<br>Control: 20.3<br>Intervention: 21.2<br>**Change: 0.90 p=0.49**<br>**CI: 1.66 - 3.47**	
**PCL - PTSD**			Mean score 3 months<br>Control: 31.6<br>Intervention: 29.8<br>**Change: -1.7 p=0.47**<br>**CI: -6.65 - 3.12**<br><br>Mean score 6 months<br>Control: 30.6<br>Intervention: 27.1<br>**Change: -3.5 p=0.056**<br>**CI: -7.12 - 0.09**
**Overall metric for comparison**	**MD: -3**	**MD: 0.9**	**MD: -7.12**

Table 11: Patient outcome measures

Study	Almoosa et al. 2009	White et al. 2018	Dykes et al. 2017
Intervention / Measure	**CPR discussions**	**Multi family support**	**Patient engagement**
**Death**	Control: 8% (3/39)<br>Intervention: 37% (17/45)<br>**Change: 31%, p<0.05***<br>**RR: 4.91, CI: 1.55-15.51**	Control: 28.5% (249/873)<br>Intervention: 36% (197/547)<br>**Change: 7.5%, p=0.008***<br>**RR: 1.27, CI: 1.09-1.48**	
**Length of stay**		Mean days<br>Control: 13.5<br>Intervention: 10.4<br>**Change: 3.1, p<0.001***<br>**CI: 0.69 - 0.87**	Mean (median) [range]<br>Pre: 4.9 (2) [1-108], n=881<br>Post: 5.0 (2) [1-115, n=904<br>**Change: 0.1 [0], p=0.61**
**Adverse events**			Pre: 59/1000 patient days<br>Post: 42/1000 patient days<br>**Change: 17, % reduction: 29%**<br>**CI: 6.95-27.05, p<0.05***
**Overall metric for comparison**	**RR: 4.91**	**RR: 1.27**	**29%**

For peer review only - http://bmjopen.bmj.com/site/about/guidelines.xhtml

For peer review only

For peer review only - http://bmjopen.bmj.com/site/about/guidelines.xhtml

Figure 1: Flow diagram

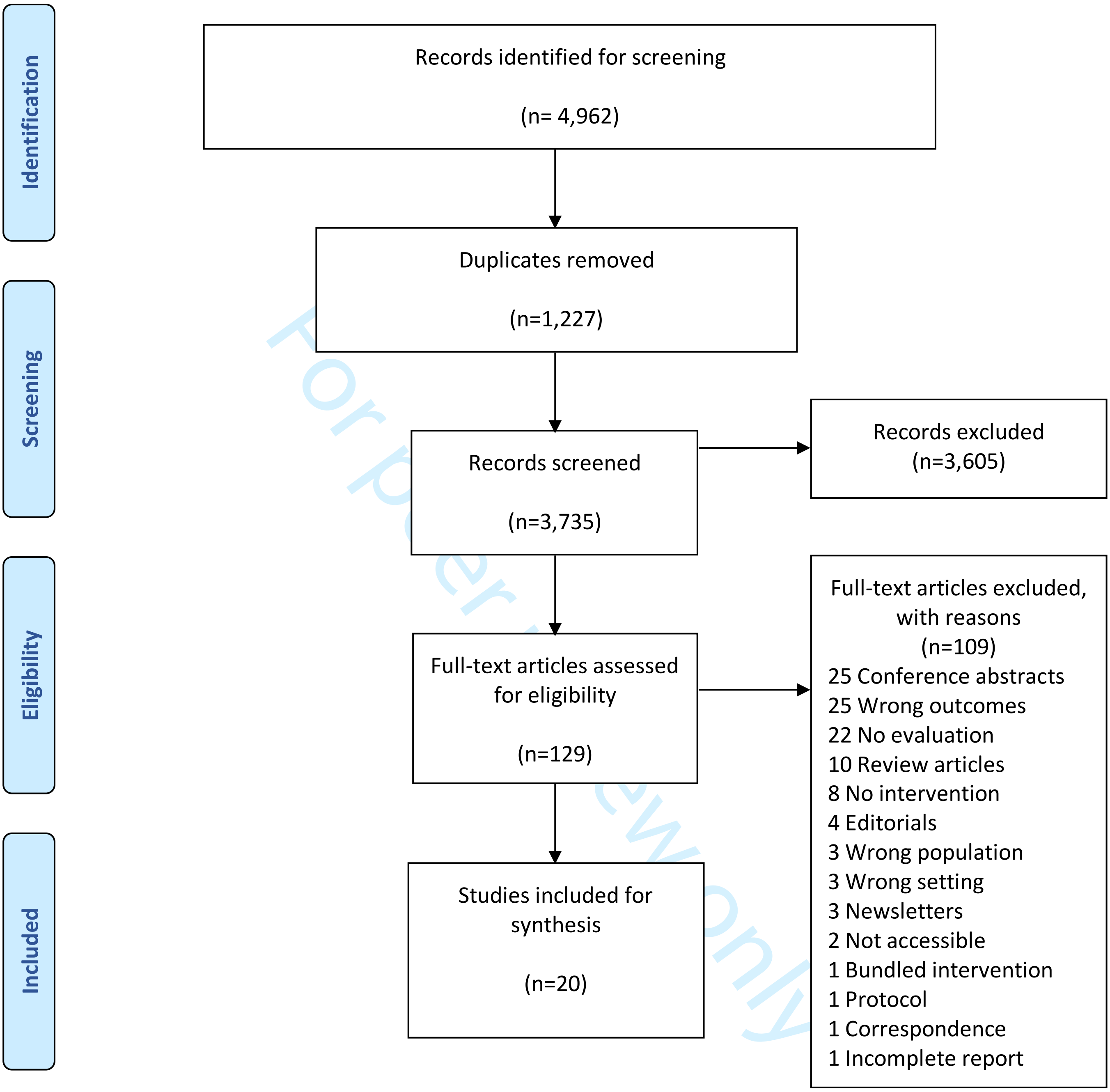

For peer review only

Figure 2: Typology of family involvement interventions.

Intervention Types / Features	ICU setting	Family time commitment	Professional input	Opportunities & Challenges	Cost	Evidence level	Engagement outcomes			Clinical outcomes		
							Satisfaction	Decision making	Communication	Wellbeing	Trauma	Patient outcomes
**Environmental unit changes** Unit change interventions consisted of complete structural redesign of an existing ICU with the objective being enhanced family member presence	General	Low	Low	+ Communication<br>+ Interaction<br>- Exposure<br>- Privacy	High	Low	Low impact	Very low impact	Low impact	Very low impact	Very low impact	Very low impact
**Web-based support** Web based and electronic interventions were approaches which utilised information and communication technology to facilitate information sharing and asynchronous communication	General/ specialist	Moderate	Moderate	+ Knowledge<br>+ Efficiency<br>- IT proficiency	Moderate	Moderate	High impact	Low impact	Very low impact	Very low impact	Very low impact	Low impact
**Discussion-based support** Discussion based interventions generally consisted of one off or regular face to face conversations which took place in the unit between family members and healthcare professionals	General/ Specialist	Moderate	High	+ Support<br>+ Wellbeing<br>+ Decision making<br>- Training	Moderate	High	Moderate impact	Very high impact	High impact	Moderate impact	Moderate impact	Low impact
**Multicomponent support** Multicomponent interventions comprised more than one technique to engage, educate and communicate with family members in the ICU	General/ Specialist	High	High	+ Family trauma<br>+ Pt outcomes<br>- Complexity<br>- Resources	High	High	Moderate impact	Very low impact	Low impact	Moderate impact	Low impact	Moderate impact
**Participation in medical rounds** Family involvement in rounds interventions enabled the family members to attend and watch the daily rounds process, with opportunity to raise questions and clarify issues	General ICU	High	Moderate	+ Knowledge<br>+ Efficiency<br>- Time<br>- Satisfaction	Low	High	Moderate impact	Very low impact	High impact	Low impact	Very low impact	Very low impact
**Participation in physical care** Physical participation in care interventions consisted of physical touch between family members and patients being utilised for reciprocal benefit	General ICU	High	Low	+ Satisfaction<br>+ Wellbeing<br>- Confidence	Low	Low	High impact	Very low impact	Very low impact	Moderate impact	Very low impact	Very low impact

For peer review only - http://bmjopen.bmj.com/site/about/guidelines.xhtml

Search strategy as applied in Medline (date last run 16.06.2019)

1. exp Critical Illness/
2. exp Critical Care/
3. exp Intensive Care Units/
4. critical care.mp.
5. critical illness*.mp.
6. intensive care.mp.
7. ICU.mp.
8. ITU.mp.
9. OR/1-8
10.exp Patient Participation/
11.patient involve*.mp.
12.patient engage*.mp.
13.shared decision making.mp.
14.patient participat*.mp.
15.service user involve*.mp.
16.family involve*.mp.
17.family participat*.mp.
18.family engage*.mp.
19.caregiver involve*.mp.
20.caregiver engage*.mp.
21.caregiver participat*.mp.
22.relative involve*.mp.
23.relative engage*.mp.
24.relative participat*.mp.
25.OR/11-24
26.9 AND 25

For peer review only

SUPPLEMENTARY FILE

Supplementary Table 1: Search strategy as applied in Medline 1
Supplementary Table 2: Participation in medical rounds interventions 2
Supplementary Table 3: Multi-component family involvement interventions 3
Supplementary Table 4: Discussion based family involvement interventions 3
Supplementary Table 5: Web-based support interventions 5
Supplementary Table 6: Environmental unit changes & Participation in care interventions... 6

Supplementary Table 1: Search strategy as applied in Medline (date last run 16.06.2019)

1. exp Critical Illness/
2. exp Critical Care/
3. exp Intensive Care Units/
4. critical care.mp.
5. critical illness*.mp.
6. intensive care.mp.
7. ICU.mp.
8. ITU.mp.
9. OR/1-8
10. exp Patient Participation/
11. patient involve*.mp.
12. patient engage*.mp.
13. shared decision making.mp.
14. patient participat*.mp.
15. service user involve*.mp.
16. family involve*.mp.
17. family participat*.mp.
18. family engage*.mp.
19. caregiver involve*.mp.
20. caregiver engage*.mp.
21. caregiver participat*.mp.
22. relative involve*.mp.
23. relative engage*.mp.
24. relative participat*.mp.
25. OR/11-24
26. 9 AND 25

For peer review only

Supplementary File: Outcomes grouped by type of family involvement intervention

Supplementary Table 2: Participation in medical rounds interventions

Study	Allen et al. 2017	Jacobowski et al. 2010	Weber et al. 2018
Intervention / Measure	Family on rounds	Family rounds	Family rounds
**Communication (improvement, % agree)**	Pre: n=49 (100%)<br>Post: n=47 (100%)<br>Change 0% p=0.68<br>RR: 1, CI: 0-0		
**Family knowledge**	Improvement, % agree<br>Pre: n=146 (35%) | Post: n=374 (88%)<br>Change 52% p<0.001<br>RR: 2.46, CI: 2.14-2.81		
**Nurse workload**	Reduction, % agree<br>Pre: n=32 (65%) | Post: n=45 (96%)<br>Change 31%<br>RR: 1.46, CI: 1.18-1.81		
**Physician satisfaction**	Pre: n=4 (80%) | Post: n= 5 (83%)<br>Change 3%<br>RR: 1.04, CI: 0.59-1.83		
**Frequency nurse communication**		% highest score<br>Pre: 64% | Post: 57%<br>Change: -7% p=0.30<br>RR: 0.89 CI: 0.70-1.12	
**Frequency doctor communication**		% highest score<br>Pre: 38% | Post: 60%<br>Change: 22% p=0.004<br>RR: 1.60 CI 1.18-2.17	
**Included in decision making**		% highest score<br>Pre: 66% | Post: 76%<br>Change: 10% p=0.12<br>RR: 1.15 CI 0.96-1.38	
**Support in decision making**		% highest score<br>Pre: 49% | Post: 69%<br>Change: 20% p=0.005<br>RR: 1.39 CI 1.09-1.79	
**Time to ask questions**		% highest score<br>Pre: 40% | Post: 23%<br>Change: -17% p=0.02<br>RR: 0.57 CI 0.37-0.90	
**FS ICU 24 - Frequency doctor communication**			% top scores<br>Control: 43%<br>Intervention: 56%<br>Change: 13% p>0.05<br>RR: 1.31 CI 0.90-1.89
**FS ICU 24 - Frequency nurse communication**			% top scores<br>Control: 62%<br>Intervention: 78%<br>Change: 16% p>0.05<br>RR: 1.24 CI 0.98-1.58
**FS-ICU (24) - Decision making**			Mean score (SD)<br>Control: 85.1 (16.3)<br>Intervention: 88.6 (14.6)<br>Change: 3.5 p=0.20<br>CI: -1.98-8.98
**FS-ICU (24) - Global score**			Mean score (SD)<br>Control: 86.0 (16.0)<br>Intervention: 90.8 (10.7)<br>Change: 4.8 p=0.20<br>CI: -0.12 - 9.72
**Support given (emotional)**			% top scores<br>Control: 54%<br>Intervention: 71%<br>Change: 17% p>0.05<br>RR: 1.32 CI 0.99-1.75
**Coordination of care**			% top scores<br>Control: 68%<br>Intervention: 77%<br>Change: 9%<br>RR: 1.13 CI 0.91-1.41

For peer review only - http://bmjopen.bmj.com/site/about/guidelines.xhtml

Supplementary Table 3: Multi-component family involvement interventions

Study	Cray 1989	Davidson et al. 2010	Marshall et al. 2016	White et al. 2018
Intervention / Measure	Family programme	Facilitated sensemaking	Nutrition intervention	Multi family support
Satisfaction with family involvement	100% agreed (76/76)	Observations showed family members were most engaged when receiving information about how to participate at the bedside.	Reports of increased family involvement, enabling patient advocacy.	
Helpful in understanding loved one's illness	93% agreed (71/76)		Improvement in knowledge regarding nutritional needs.	
HADS - global score (0-42)				Mean score<br>Control: 12.0<br>Intervention: 11.7<br>Change: -0.34 p=0.61<br>CI −1.67 - 0.99
QOC - clinician-family communication (0-100)				Mean score<br>Control: 62.7<br>Intervention: 69.1<br>Change: 6.39 p=0.001<br>CI 2.57 - 10.20
PPPC - Patient Perception of Patient Centeredness (1-4)				Mean score<br>Control: 1.8<br>Intervention: 1.7<br>Change: -0.15 p=0.006<br>CI −0.26 - −0.04
Length of stay - Hospital				Mean score<br>Control: 6.7<br>Intervention: 7.4<br>Change: 0.90 p=0.045<br>CI 0.81 - 1.00
Length of stay - ICU				Mean score<br>Control: 13.5<br>Intervention: 10.4<br>Change: 0.77 p<0.001<br>CI 0.69 - 0.87
IES - PTSD (0-88)				Mean score<br>Control: 20.3<br>Intervention: 21.2<br>Change: 0.90 p=0.49<br>CI −1.66 - 3.47

Supplementary Table 4: Discussion based family involvement interventions

Study	Almoosa et al. 2009	Hollman et al. 2018	Garrouste et al. 2016	Huang et al. 2018	Curtis et al. 2016	Shaw et al. 2014
Intervention / Measure	CPR discussions	Health conversations	Interprofessional family conference	Primary physician involvement	Communication facilitator	Team training
Satisfaction with CPR Discussion	Questions answered 84% (36/43) CI 70-91<br>Facilitated decision 77% (33/42) CI 64-88					
Time to decision	Days mean (sd)<br>Control 7.4 (3.2)<br>Intervention 6.6 (6.5)<br>Change -0.8 days<br>CI -1.48-3.08					
Death	Control 8% (3/39)<br>Intervention 37% (17/45)<br>Change 31%<br>RR 4.91 CI 1.55-15.51					
Change to less aggressive preference	Control 18% (8/39)<br>Intervention 25% (11/45)<br>RR: 1.19 CI 0.53-2.66					
Inclusion in decision making		Qualitative findings of greater family engagement with ICU nurses.		% completely satisfied<br>Control: 61.4%<br>Intervention: 75.9%<br>Change: 14.5%<br>p=0.05<br>RR: 1.23 CI 1.03-1.49		

For peer review only

Measure \ Study / Intervention	Almoosa et al. 2009	Hollman et al. 2018	Garrouste et al. 2016	Huang et al. 2018	Curtis et al. 2016	Shaw et al. 2014
**Intervention**	**CPR discussions**	**Health conversations**	**Interprofessional family conference**	**Primary physician involvement**	**Communication facilitator**	**Team training**
**HADS - anxiety**			At 90 days, Median [IQR]<br>Control: 8 [4.5-12]<br>Intervention: 4 [1-9]<br>Change: 4 p=0.01<br><br>% sign. Anxiety (>8)<br>Control: 52.3% (n=23)<br>Intervention: 33.3% (n=14)<br>Change: 19% p=0.08<br>RR: 0.95, CI 0.63-1.44			
**HADS - depression**			At 90 days, Median [IQR]<br>Control: 5.5 [1-11.5]<br>Intervention: 2 [0-6]<br>Change: -3.5 p=0.04<br><br>% sign. Depress. score > 8<br>Control: 38.6% (n=17)<br>Intervention: 23.8% (n=10)<br>Change: 14.8% p=0.14<br>RR: 0.61, CI 0.31-1.18			
**PDEQ**			Median [IQR]<br>Control: 14.5 [11-23]<br>Intervention: 13 [0-17]<br>Change: -1.5 p=0.17			
**IES-R**			At 90 days, Median [IQR]<br>Control: 24 [12.5-45]<br>Intervention: 21 [9-33]<br>Change: -3 p=0.24			
**FS-ICU (24) - ICU Care**				Mean (sd)<br>Control: 88.38 (14.5)<br>Intervention: 90.22 (12.68)<br>Change: 1.84 p=0.28<br>CI -2.42 - 6.09		
**FS-ICU (24) - Global score**				Mean (sd)<br>Control: 84.91 (12.17)<br>Intervention: 86.4 (11.76)<br>Change: 1.49 p=0.16<br>CI -2.14 - 5.12		
**FS-ICU (24) - Decision making**				Mean (sd)<br>Control: 80.07 (12.76)<br>Intervention: 81.06 (15.1)<br>Change: 0.99 p=0.16<br>CI -3.00 - 4.98		
**Control over patient care**				% completely satisfied<br>Control: 55.6%<br>Intervention: 73.6%<br>Change: 18% p=0.02<br>RR: 1.31 CI 1.07-1.61		
**GAD 7 - anxiety**					Mean score 3 months<br>Control: 3.0<br>Intervention: 2.3<br>Change: -0.7 p=0.50<br>CI: -2.91 - 1.42<br><br>Mean score 6 months<br>Control: 2.7<br>Intervention: 1.8	

Study / Intervention / Measure	Almoosa et al. 2009 CPR discussions	Hollman et al. 2018 Health conversations	Garrouste et al. 2016 Interprofessional family conference	Huang et al. 2018 Primary physician involvement	Curtis et al. 2016 Communication facilitator	Shaw et al. 2014 Team training
					Change: -0.9 p=0.43<br>CI: -3.10 - 1.32	
**PHQ 9 - depression**					Mean score 3 months<br>Control: 4.9<br>Intervention: 3.1<br>Change: -1.8 p=0.09<br>CI: -3.89 - 0.31<br><br>Mean score 6 months<br>Control: 4.7<br>Intervention: 2.4<br>Change: -2.3 p=0.01<br>CI: -4.30 - -0.42	
**PCL - PTSD**					Mean score 3 months<br>Control: 31.6<br>Intervention: 29.8<br>Change: -1.7 p=0.47<br>CI: -6.65 - 3.12<br><br>Mean score 6 months<br>Control: 30.6<br>Intervention: 27.1<br>Change: -3.5 p=0.056<br>CI: -7.12 - 0.09	
**Staff confidence. All items improved, biggest change: confidence in encouraging family presence**						Mean score (SD)<br>Pre: 7.0 (1.7)<br>Post: 8.4 (1.1)<br>Change: 1.4<br>p<0.001<br>CI: 0.81-1.99
**FS ICU 24 - Frequency nurse communication**						Mean score<br>Pre: 79.2<br>Post: 87.18<br>Change: 7.98<br>p=0.04
**FS ICU 24 - Frequency doctor communication**						Mean score<br>Pre: 67.86<br>Post: 76.69<br>Change: 8.83<br>p=0.04
**FS ICU 24 - Honesty of information**						Mean score<br>Pre: 77.78<br>Post: 87.08<br>Change: 9.30<br>p=0.01
**FS-ICU (24) - Decision making**						Mean score<br>Pre: 77.47<br>Post: 83.32<br>Change: 5.85<br>p=0.05
**FS-ICU (24) - Global score**						Mean score<br>Pre: 83.21<br>Post: 85.69<br>Change: 2.48<br>p=0.32

Supplementary Table 5: Web-based support interventions

Study / Intervention / Measure	Dalal et al. 2015 Patient centred toolkit	Dykes et al. 2017 Patient engagement ICT	Ernecoff et al. 2016 Tablet decision support	Huffines et al. 2013 Support care algorithm
**Usability**	Mean (sd) usability score<br>m 74 (16.7)			
**Satisfaction**	Satisfaction 72% (13/18)<br>Mean (sd) satisfaction score<br>4.06 (0.94)			

For peer review only - http://bmjopen.bmj.com/site/about/guidelines.xhtml

Study / Intervention / Measure	Dalal et al. 2015	Dykes et al. 2017	Ernecoff et al. 2016	Huffines et al. 2013
Intervention	Patient centred toolkit	Patient engagement ICT	Tablet decision support	Support care algorithm
**HCAHPS**		% top score 9-10<br>Pre: 71.8%, n=53, CI 61.1-82.6<br>Post: 93.3%, n=58, CI 88.2-98.4<br>Change 21.5<br>RR 1.33 CI 1.10-1.55		
**FS-ICU (20)**		Mean score<br>Pre: 84.3, n=106, CI 81.3-87.3<br>Post: 90, n=156, CI 88.1-91.9<br>Change: 5.7, CI 2.31-9.09		
**Adverse events**		Pre: 59/1000 patient days<br>Post: 42/1000 patient days<br>Change 17, CI 6.95-27.05<br>Relative reduction 29%		
**Length of stay**		Mean (median) LoS [range]<br>Pre: 4.9 (2) [1-108], n=881<br>Post: 5.0 (2) [1-115, n=904<br>p=0.61		
**Share in decisions about care planning**			Qualitative perceptions of improving family engagement, communication and involvement in decision making.	Mean satisfaction (1-4)<br>Pre: 2.97 | Post: 3.41<br>Change: 0.44 p=0.07<br><br>% scoring excellent<br>Pre: 45% | Post: 68%<br>Change: 23% p=0.009<br>RR: 1.52 CI 1.03-2.24
**Support and encouragement given**				Mean satisfaction (1-4)<br>Pre: 3.38 | Post: 3.52<br>Change: 0.15 p=0.47<br><br>% scoring excellent<br>Pre: 60% | Post 75%<br>Change: 15% p=0.14<br>RR: 1.23 CI 0.91-1.65
**Nurses and doctors working together as a team**				Mean satisfaction (1-4)<br>Pre: 3.38 | Post: 3.73<br>Change: 0.35 p=0.04<br><br>% scoring excellent<br>Pre: 64% | Post: 83%<br>Change: 19% p=0.04<br>RR: 1.31 CI 1.00-1.71

Supplementary Table 6: Environmental unit changes & Participation in care interventions

Study / Intervention / Measure	Choi et al 2013	Rippin et al. 2015	Prichard & Newcomb 2015
	Environmental unit changes		Physical participation in care
Intervention	PFC Unit design	Family centred design	Hand massage
**Family interaction with patient**	r=0.632 | p<0.01		
**Family interaction with staff**	r=0.552 | p<0.01	Qualitative observations of increased, family-initiated interactions.	
**HADS - anxiety**			Mean change in score<br>Control: -0.4<br>Intervention: -3.87<br>Change: -3.47 p=0.002<br>CI -5.5 - -1.4
**HADS - depression**			Mean change in score<br>Control: -0.3<br>Intervention: -2.5<br>Change: -2.2 p=0.10<br>CI -0.49 - 4.7

For peer review only - http://bmjopen.bmj.com/site/about/guidelines.xhtml

Reporting checklist for systematic review and meta-analysis.

Based on the PRISMA guidelines.

Instructions to authors

Complete this checklist by entering the page numbers from your manuscript where readers will find each of the items listed below.

Your article may not currently address all the items on the checklist. Please modify your text to include the missing information. If you are certain that an item does not apply, please write "n/a" and provide a short explanation.

Upload your completed checklist as an extra file when you submit to a journal.

In your methods section, say that you used the PRISMAreporting guidelines, and cite them as:

Moher D, Liberati A, Tetzlaff J, Altman DG, The PRISMA Group. Preferred Reporting Items for Systematic Reviews and Meta-Analyses: The PRISMA Statement

For peer review only

		Reporting Item	Page Number
**Title**			
	#1	Identify the report as a systematic review, meta-analysis, or both.	1
**Abstract**			
Structured summary	#2	Provide a structured summary including, as applicable: background; objectives; data sources; study eligibility criteria, participants, and interventions; study appraisal and synthesis methods; results; limitations; conclusions and implications of key findings; systematic review registration number	2
**Introduction**			
Rationale	#3	Describe the rationale for the review in the context of what is already known.	4
Objectives	#4	Provide an explicit statement of questions being addressed with reference to participants, interventions, comparisons, outcomes, and study design	5

		(PICOS).	
**Methods**			
Protocol and registration	#5	Indicate if a review protocol exists, if and where it can be accessed (e.g., Web address) and, if available, provide registration information including the registration number.	5
Eligibility criteria	#6	Specify study characteristics (e.g., PICOS, length of follow-up) and report characteristics (e.g., years considered, language, publication status) used as criteria for eligibility, giving rational	5
Information sources	#7	Describe all information sources in the search (e.g., databases with dates of coverage, contact with study authors to identify additional studies) and date last searched.	5
Search	#8	Present full electronic search strategy for at least one database, including any limits used, such that it could be repeated.	5
Study selection	#9	State the process for selecting studies (i.e., for screening, for determining eligibility, for inclusion in the systematic review, and, if applicable, for inclusion in the meta-analysis).	5-6
Data collection process	#10	Describe the method of data extraction from reports (e.g., piloted forms, independently by two reviewers) and any processes for obtaining and confirming data from investigators.	5-6
Data items	#11	List and define all variables for which data were sought (e.g., PICOS, funding sources), and any assumptions and simplifications made.	5-6
Risk of bias in individual studies	#12	Describe methods used for assessing risk of bias in individual studies (including specification of whether this was done at the study or outcome level, or both), and how this information is to be used in any data synthesis.	5-6
Summary measures	#13	State the principal summary measures (e.g., risk ratio, difference in means).	5-6
Planned methods of analyis	#14	Describe the methods of handling data and combining results of studies, if done, including measures of consistency (e.g., I2) for each meta-analysis.	6
Risk of bias across studies	#15	Specify any assessment of risk of bias that may affect the cumulative evidence (e.g., publication bias, selective reporting within studies).	5-6
Additional	#16	Describe methods of additional analyses (e.g., sensitivity or subgroup	6

For peer review only

analyses		analyses, meta-regression), if done, indicating which were pre-specified.	
**Results**			
Study selection	#17	Give numbers of studies screened, assessed for eligibility, and included in the review, with reasons for exclusions at each stage, ideally with a flow diagram.	6
Study characteristics	#18	For each study, present characteristics for which data were extracted (e.g., study size, PICOS, follow-up period) and provide the citation.	6-7
Risk of bias within studies	#19	Present data on risk of bias of each study and, if available, any outcome-level assessment (see Item 12).	7
Results of individual studies	#20	For all outcomes considered (benefits and harms), present, for each study: (a) simple summary data for each intervention group and (b) effect estimates and confidence intervals, ideally with a forest plot.	7-8
Synthesis of results	#21	Present the main results of the review. If meta-analyses are done, include for each, confidence intervals and measures of consistency.	8-9
Risk of bias across studies	#22	Present results of any assessment of risk of bias across studies (see Item 15).	9-10
Additional analysis	#23	Give results of additional analyses, if done (e.g., sensitivity or subgroup analyses, meta-regression [see Item 16]).	9-10
**Discussion**			
Summary of Evidence	#24	Summarize the main findings, including the strength of evidence for each main outcome; consider their relevance to key groups (e.g., health care providers, users, and policy makers	10-11
Limitations	#25	Discuss limitations at study and outcome level (e.g., risk of bias), and at review level (e.g., incomplete retrieval of identified research, reporting bias).	10-11
Conclusions	#26	Provide a general interpretation of the results in the context of other evidence, and implications for future research.	11
**Funding**			
Funding	#27	Describe sources of funding or other support (e.g., supply of data) for the systematic review; role of funders for the systematic review.	11

For peer review only

The PRISMA checklist is distributed under the terms of the Creative Commons Attribution License CC-BY.
This checklist was completed on 08. July 2020 using https://www.goodreports.org/, a tool made by the EQUATOR Network in collaboration with Penelope.ai

For peer review only

BMJ Open

Interventions to promote family member involvement in adult critical care settings: A systematic review.

Journal:	*BMJ Open*
Manuscript ID	bmjopen-2020-042556.R2
Article Type:	Original research
Date Submitted by the Author:	24-Feb-2021
Complete List of Authors:	Xyrichis, Andreas; King's College London<br>Fletcher, Simon; Kingston and St Georges University London, Health, Social Care and Education<br>Philippou, Julia; King's College London, Nursing & Midwifery<br>Brearley, Sally; King's College London, Florence Nightingale Faculty of Nursing and Midwifery<br>Terblanche, Marius; Guy's and St Thomas' NHS Foundation Trust, Critical Care<br>Rafferty, Anne Marie; King's College London, Florence Nightingale School of Nursing and Midwifery
**Primary Subject Heading**:	Intensive care
Secondary Subject Heading:	Intensive care, Patient-centred medicine, Health services research, Health policy, Evidence based practice
Keywords:	Adult intensive & critical care < INTENSIVE & CRITICAL CARE, Quality in health care < HEALTH SERVICES ADMINISTRATION & MANAGEMENT, Health policy < HEALTH SERVICES ADMINISTRATION & MANAGEMENT

SCHOLARONE™
Manuscripts

I, the Submitting Author has the right to grant and does grant on behalf of all authors of the Work (as defined in the below author licence), an exclusive licence and/or a non-exclusive licence for contributions from authors who are: i) UK Crown employees; ii) where BMJ has agreed a CC-BY licence shall apply, and/or iii) in accordance with the terms applicable for US Federal Government officers or employees acting as part of their official duties; on a worldwide, perpetual, irrevocable, royalty-free basis to BMJ Publishing Group Ltd ("BMJ") its licensees and where the relevant Journal is co-owned by BMJ to the co-owners of the Journal, to publish the Work in this journal and any other BMJ products and to exploit all rights, as set out in our licence.

The Submitting Author accepts and understands that any supply made under these terms is made by BMJ to the Submitting Author unless you are acting as an employee on behalf of your employer or a postgraduate student of an affiliated institution which is paying any applicable article publishing charge ("APC") for Open Access articles. Where the Submitting Author wishes to make the Work available on an Open Access basis (and intends to pay the relevant APC), the terms of reuse of such Open Access shall be governed by a Creative Commons licence – details of these licences and which Creative Commons licence will apply to this Work are set out in our licence referred to above.

Other than as permitted in any relevant BMJ Author's Self Archiving Policies, I confirm this Work has not been accepted for publication elsewhere, is not being considered for publication elsewhere and does not duplicate material already published. I confirm all authors consent to publication of this Work and authorise the granting of this licence.

For peer review only

TITLE PAGE

Interventions to promote family member involvement in adult critical care settings: A systematic review.

Andreas Xyrichis (corresponding author): Kings College London, James Clerk Maxwell Building, Waterloo, London, andreas.xyrichis@kcl.ac.uk
Simon Fletcher: Kingston & St Georges University London, Hunter Wing 6th Floor, St Georges University, Tooting, London, simon.fletcher@sgul.kingston.ac.uk
Julia Philippou: Kings College London, James Clerk Maxwell Building, Waterloo, London, julia.philippou@kcl.ac.uk
Sally Brearley: Kingston & St Georges University London, Hunter Wing 6th Floor, St Georges University, Tooting, London, sally.brearley@icloud.com
Marius Terblanche: Critical Care, Guy's and St Thomas' NHS Foundation Trust, Westminster Bridge Road, London, Marius.Terblanche@gstt.nhs.uk
Anne Marie Rafferty: Kings College London, James Clerk Maxwell Building, Waterloo, London, anne_marie.rafferty@kcl.ac.uk

For peer review only

ABSTRACT

Objective: To identify, appraise and synthesise evidence of interventions designed to promote family member involvement in adult critical care units; and to develop a working typology of interventions for use by health professionals and family members.

Design: Mixed-method systematic review.

Data sources: Bibliographic databases were searched without date restriction up to June 2019: MEDLINE, EMBASE and CINAHL; the Cochrane Central Register of Controlled Trials, Joanna Briggs and Cochrane Libraries. Back issues of leading critical care and patient experience journals were manually searched, as were the reference lists of included studies. All evaluation studies of relevant intervention activities were included; all research designs and outcome measures were eligible. Due to heterogeneity in interventions, designs and outcome measures the synthesis followed a narrative approach. Service users met with the research team termly.

Results: Out of 4,962 possible citations a total of 20 studies were included. The overall evidence base was assessed as moderate to weak. Six categories of interventions were identified: environmental unit changes (n=2), web-based support (n=4), discussion-based support (n=6), multicomponent support (n=4), participation in rounds (n=3), and participation in physical care (n=1). Clinical and methodological heterogeneity across studies hindered meta-analysis, hence a narrative synthesis was pursued. Six main outcomes were identified, grouped under two categories: i) Involvement outcomes: Communication (*mean difference* ranged from: 6.39 to 8.83), Decision making (*mean difference* ranged from: -0.8 to 5.85), Satisfaction (*mean difference* ranged from: 0.15 to 2.48); and ii) Health outcomes: Family Trauma (*mean difference* ranged from: -7.12 to 0.9), Family Wellbeing (*mean difference* ranged from: -0.7 to -4), Patient outcomes (*relative risk* ranged from: 1.27 to 4.91). The findings from the qualitative studies were thematically analysed to identify features of the interventions that participants perceived to influence effectiveness. Synthesised into five overarching categories (practicality, development, interaction, reflexivity, bridging) these can serve as principles to inform the future design and development of more refined family member involvement interventions.

Conclusions: Future interventions should be developed with much closer family member input and designed by considering the key features we identified. We call for future interventions to be multi-layered and allow for a greater or lesser level, and different kind, of involvement for family members. Choice of intervention should be informed by a baseline diagnostic of family members’ needs, readiness and preparedness for involvement.

Review registration: PROSPERO 2018 CRD42018086325

Keywords: Critical care units, family involvement, systematic review, typology, service user

For peer review only - http://bmjopen.bmj.com/site/about/guidelines.xhtml

ARTICLE SUMMARY

Strengths and limitations of this study

- We completed a comprehensive mixed-method systematic review of available evidence on interventions for family member involvement in adult critical care.
- We involved a Service Users & Carers group throughout the systematic review process, which included 16 hours of in-depth group discussions.
- Study screening, selection, quality assessment and data extraction was completed independently and in duplicate by two review authors.
- Methodological and clinical heterogeneity across studies prevented us from pursuing a meta-analysis.
- Qualitative evidence was synthesised thematically to identify features of the interventions that participants perceived to influence effectiveness.

For peer review only

BACKGROUND

Family member involvement in healthcare is recognised by policymakers and the public as an invaluable aspect of healthcare provision. The move towards the more structured integration of family members is consistent with the call for greater patient-centred care, long invoked by a (2001) report from the Institute of Medicine[1]. Emphasising the potential benefits in actively involving family members in decision-making processes and care management was regarded as a key contributor to the reduction of the risk of medical error and a broader improvement in quality. The idea that a 'fully-engaged' public[2] should be encouraged has also been evident in United Kingdom contexts, however governmental scrutiny over the proceeding decade has revealed that this has yet to be comprehensively realised. Two strategic reports from NHS England[3,4] explored a practical move towards a healthcare system which is built around the patient, incorporating families representing a key means of achieving this.

For the purposes of the present review, family member involvement refers to the activities of different professionals to improve care by ensuring family members are involved in decision making, sharing of information, power and responsibility for patient needs and choices. Family member involvement is especially relevant in the intensive care unit (ICU), where it can have profound consequences for care decisions, delivery and outcomes; this is partly because ICU patients are rarely in a position to communicate directly with clinicians and recollect their ICU experience, which means that the responsibility for this often lies with their family members. While this is a concern in healthcare systems worldwide, and especially in the UK, a recent mixed-methods study found there is still great variation in family satisfaction across ICUs in England, Wales and Northern Ireland[5].

Studies over the past decade have confirmed that family members can have a positive influence on a patients' care and recovery from ICU; but also that family members themselves can be affected even after discharge[6-11]. In particular, within the first few days after ICU admission, family members can show signs of anxiety, depression and stress; report difficulties in understanding the information clinicians try to communicate with them; and those who suffered a bereavement are at risk of generalized anxiety, panic attacks, depression, and post-traumatic stress syndrome (PTSD)[7]. For example, one study[6] surveyed family members of patients who have been in ICU to find that 90 days after discharge more than a third (34%) of them suffered from PTSD symptoms. In addition, they noted higher rates (48%) among those family members who indicated the information they were given was incomplete.

While there is a growing evidence base on patients, family members and clinicians' perceptions of involvement in ICU care, we are still missing a standardised, evidence-based approach to facilitate this process. Indeed, a recent scoping review[10] sought to investigate the extent and range of literature on this topic, finding evidence of a growth in papers with over 100 reports identified. However, they did not seek to assess the quality of the evidence nor did they examine the type of interventions available, and key features that foster

For peer review only - http://bmjopen.bmj.com/site/about/guidelines.xhtml

effectiveness. While this is an area of growing interest, we are still unclear about the range and quality of interventions available to promote family member involvement in ICUs.

METHODS

Our review sought to answer the following question: What are the available interventions, and which are most effective, for fostering family member involvement in adult critical care settings? Four objectives were set: a) undertake a comprehensive and systematic search of published and unpublished studies reporting on interventions that promote involvement in adult critical care; b) robustly assess the quality of empirical evidence for all included studies; c) generate a detailed description and synthesis of interventions and their associated outcomes; and d) classify interventions and outcomes in order to develop a typology of interventions, outlining key factors that support or impede involvement.

Study eligibility criteria

We included evaluation studies of any design, including experimental and quasi-experimental studies, as well as action research, case study and ethnographic designs. We considered reports of any kind of interventions as long as they were intended to promote the participation of family members in adult critical care. We included studies that reported on a mixture of relevant outcomes, including standard measures such as the Hospital Anxiety and Depression Score (HADS); and non-standard but important indicators such as family satisfaction. We excluded non-evaluation papers and those with populations, outcomes and settings that did not match our brief. One study reported on a bundled intervention including awakening and breathing coordination, delirium monitoring and management, early mobility, and family-centred care; this study had to be excluded because we could not discern outcomes specifically linked to the family involvement component of the bundle.

Search for evidence

We searched (June 2019) MEDLINE, EMBASE, CINAHL and the Cochrane Central Register of Controlled Trials following a systematic approach without date restriction. We also performed searches for clinical studies through the WHO Trials Registry. We hand searched recent back issues of key critical care and patient experience e-journals, scanned reference lists of identified reviews and included articles; searched our personal libraries and consulted with experts in the field. GoogleScholar was also searched. Non-commercially published (Grey) literature was sought through the OpenGrey and the GreyLit Report databases. A standardised search strategy was developed with the input of an information specialist and applied to all databases, utilising both MeSH and freetext terms (Supplementary file).

Data management, screening, extraction and quality assessment

Search results were imported into Covidence, the standard production platform for Cochrane Reviews. Two reviewers (AX, SF) independently screened all citations in duplicate against the inclusion criteria. Any disagreement was resolved through discussion or by involving a third

For peer review only - http://bmjopen.bmj.com/site/about/guidelines.xhtml

reviewer (JP). Reasons for excluding papers read in full were recorded and a PRISMA flowchart was completed. Standardised data extraction forms were used by two reviewers (SF, AX) to independently extract key information. Any disagreements found at this stage were also resolved through a consensus approach, or the involvement of a third reviewer (JP). We assessed each included paper for methodological rigour using an established rating approach utilised successfully in a number of previous mixed-method systematic reviews[12,13]. This rating approach assesses ‘quality of study’ to consider: appropriate fit between study design/research questions and use of appropriate analyses; and, ‘quality of information’ to assess for a clear rationale for the intervention, good contextual information and risk of bias. Quality assessment was undertaken independently by two reviewers (AX, SF) in duplicate. For each quality domain we described the procedures reported in the paper, using verbatim quotes as necessary.

Data synthesis

The overall approach to the synthesis followed a segregated methodologies approach[14]. Accordingly, a separate synthesis of quantitative and qualitative data was initially completed followed by a mixed-method synthesis. Quantitative studies were grouped depending on study design, and results summarised using descriptive statistics. Because heterogeneity precluded statistical combination through a meta-analysis, a narrative approach to the quantitative synthesis was pursued. Synthesis of qualitative data followed the Best-fit framework approach, involving identification and grouping of thematic categories around factors influencing effectiveness of family member involvement interventions[15]. The mixed-method synthesis utilised the quantitative data to inform on the measured effects, while the qualitative data informed on the perceived effects. In this way, the qualitative and quantitative data acted as complementary rather than as confirmatory of each other. A key element of the mixed-method synthesis was to classify interventions and outcomes in order to develop a typology of family involvement interventions in ICU. To develop the typology, we identified categories for the different interventions used in the studies by noting their distinguishing characteristics. We also formed and refined key family involvement features of the interventions, such as required time commitment from families and staff, opportunities and challenges, and cost. We tabulated these alongside an indication of the quality level of the current body of evidence, and the expected impact on important engagement and clinical outcomes. The typology was developed in collaboration with a Service Users & Carers Group over two face to face meetings, during which the overall findings of the review and ways of representing these visually were discussed. The resulting typology is illustrated in a matrix-like format presenting the intervention categories in a continuum from low to high involvement.

Patient and public involvement

From the outset, the development of this project was grounded in patient and public involvement (PPI) since family member involvement in ICU has been identified as a priority area for research by the James Lind Alliance (http://www.jla.nihr.ac.uk) through a priority

For peer review only - http://bmjopen.bmj.com/site/about/guidelines.xhtml

setting partnership involving patients, carers and clinicians. ICU clinicians and patients raised our awareness on the lack of evidence-based advice on interventions that promote family involvement in ICU, and the challenges this introduces to daily practice. We included a PPI expert on our team (SB) and a frontline ICU clinician (MT) with whom we developed the project application and review protocol. We also invited feedback on our initial plans from the UK ICUSteps Charity ensuring the project was attuned to the sensitivities and concerns of service users. As part of the review process, a Service Users & Carers Group was established, the members of which were recruited through promotion in social media, the UK ICUSteps charity and the NIHR INVOLVE website. Consisting of eight ICU survivors and carers, the group was consulted through each step of the process and engaged in four strategically positioned meetings leading to over 16 hours of focussed and in-depth face-to-face discussions concerning the design, conduct, reporting and dissemination of the review. By having an engaged service user and carer group work with us throughout the completion of this review, we were able to look beyond the evidence by exploring the experiences and views of service users about the different interventions identified.

RESULTS

Overview of included studies

Search results

Out of 4,962 possible citations a total of 20 studies were included (Figure 1). Published between 1989 and 2018, they were predominantly from North American contexts (n=17), and from a range of ICU settings including surgical, neuroscience, medical oncology and trauma ICUs. One study came from a French setting, and others from Swedish and Australian ICU settings.

Study designs

Most of the studies followed a quantitative approach (n=15), but we also included four qualitative and one mixed methods study (see Table 1). Specifically, quantitative studies included randomised controlled trials, cluster-randomised and stepped-wedge trials, non-randomised before and after studies, and prospective observational multi-centre studies. Qualitative data from all studies came from semi-structured interviews. Sample sizes across the quantitative studies ranged from 30 to 1,106 family members; and in the qualitative studies from 10 to 54 family members.

Intervention types

Interventions were grouped based on their distinguishing characteristics (summarised in Table 2), and positioned within a continuum of low to high involvement: i) environmental unit changes (n=2)[16-17], ii) web-based support (n=4)[18-21], iii) discussion-based support (n=6)[22-27], iv) multicomponent support (n=4)[28-31], v) participation in rounds (n=3)[32-34], and vi) participation in physical care (n=1)[35]. Environmental unit change interventions

For peer review only - http://bmjopen.bmj.com/site/about/guidelines.xhtml

consisted of complete structural redesign of an existing ICU with the specific objective being enhanced family member presence. Web based and electronic interventions were approaches which utilised information and communication technology to facilitate information sharing and asynchronous communication. Discussion-based interventions generally consisted of one off or repeated face-to-face conversations which took place in the unit between family members and healthcare professionals. Multi-component interventions comprised more than one technique to engage, educate and communicate with family members in the ICU. Family involvement in rounds interventions enabled the family members to attend and watch the daily rounds process, with opportunity to raise questions and clarify issues. Physical participation in care consisted of physical touch between family members and patients being utilised for reciprocal benefit.

Quality of evidence

The overall evidence base was assessed to be moderate to weak, with only a few exceptions of high-quality quantitative and qualitative studies (Tables 3 and 4). The main weaknesses of the quantitative studies were related to inadequate randomisation and blinding, and lacking a control group for comparison purposes. The qualitative studies were in the main of a better quality, with key weaknesses being related to inadequate researcher reflexivity and theorisation of findings.

Quantitative results

Six main outcome categories were identified from the studies analysed for the review, grouped under two groups. Involvement outcomes: Communication, Decision making, Satisfaction; and Health outcomes: Family Wellbeing, Family Trauma, Patient outcomes. A summary of key results is provided below, with detailed reporting of outcome measures available in the tables; in Supplementary Tables 1-6 the outcome measures are presented according to intervention type.

Involvement outcomes

Communication (Table 5): Improved communication was considered as an outcome in five studies[32,33,27,34,31], the majority of which involved family participation in rounds, reporting on four measurement tools. Three studies[33,27,34] utilised the Family Satisfaction ICU (FS ICU 24) tool, specifically reporting on three dimensions: honesty of information, frequency of doctor communication, frequency of nurse communication. One study[31] reported on the Quality of Communication (QOC) tool and another used a self-developed questionnaire[32]. Mean improvements were found in all studies, although not all results reached statistical significance. The biggest effect was seen on the frequency of doctor communication, with one study of family participation in rounds showing a statistically significant improvement of 60% (RR: 1.60, CI 1.18 to 2.17, p=0.004)[33].

For peer review only - http://bmjopen.bmj.com/site/about/guidelines.xhtml

Decision making (Table 6): Six of the included studies[22,25,21,33,27,34] looked at decision making as an outcome, most of which were discussion-based interventions, through six measurement tools. The 'decision making' sub-scale of the FS ICU 24 revealed improvements in three studies although only one[27], targeting the whole interprofessional team, found a statistically significant result (MD: 5.85, p=0.05). Inclusion in decision making was also considered, as the percentage change in family members reporting complete satisfaction or giving the highest score, with one study[25] which involved the patient's primary physician showing a statistically significant improvement of 23% (RR: 1.23, CI: 1.03 to 1.49, p=0.05). Studies also noted improvements with regard to 'control over patient care' (RR: 1.31, CI: 1.07 to 1.61, p=0.02)[25], 'support in decision making' (RR: 1.39, CI: 1.09 – 1.79)[21] and 'share in decisions about care planning' (23%, RR: 1.52, CI: 1.03 to 2.24, p=0.009)[21].

Satisfaction (Table 7): Satisfaction was considered as an outcome in nine studies [28,18,19,25,21,33,27,34,31] using six different measures, and reporting on a mixture of discussion-, rounds-, multi-component- and web- based interventions. The majority of studies (n=4) measured family satisfaction with care using the FS-ICU, while the remaining studies used self-developed tools. One of the studies[19] also used the HCAHPS (Hospital Consumer Assessment of Healthcare Providers and Systems) Survey. This is a national, standardized, publicly reported survey of patients' perspectives of hospital care in the US. Mean improvements on the FS ICU were found in all studies, ranging from 1.49 to 5.7, although only one[19] which used a web-based engagement intervention reached statistical significance (MD: 5.7, CI: 2.31 to 9.09, p<0.05). Statistically significant improvements were also noted on the HCAHPS (RR: 1.33, CI: 1.10 to 1.55, p<0.05)[19].

Health outcomes

Family wellbeing was considered through measurements for anxiety and depression.

Anxiety (Table 8): Four studies, which encouraged family participation in discussions and personal care, considered anxiety as an outcome[23,35,26,31] using three different ways for measurement. Three studies[23,35,31] used the HADS. The fourth study[26] used the Generalised Anxiety Disorder Assessment (GAD-7). All four studies identified a reduction in family members' anxiety scores post the intervention, with the effect ranging from -0.34 to -4. The minimal clinically important difference of HADS has been suggested to be between -1.5 and -2, therefore the reduction noted in these papers could be of clinical significance. However, only two of these studies found statistical significance[23,35].

Depression (Table 9): Depression was an outcome also reported by three of the above studies[23,35,26] using two measures. Two studies[23,35] used the depression subscale of the Hospital Anxiety and Depression Scale (HADS) while another[26] used the Patient Health Questionnaire (PHQ-9). Both studies that used the HADS identified a reduction in depression among family members, although only one[23] reached statistical significance (p=0.04) with a median reduction of 3.5. Results on the PHQ-9 also showed a statistically significant mean reduction in scores at six months (MD: -2.3, CI: -4.30 to -0.42, p=0.01)[26].

For peer review only - http://bmjopen.bmj.com/site/about/guidelines.xhtml

Family Trauma (Table 10): Three studies considered the impact of interventions (multicomponent-, web- and discussion-based) on family member ICU-induced trauma using four different measurement tools[23,26,31]: Post-Traumatic Stress Disorder (PTSD) Checklist (PCL), Impact of Event Scale (IES) and Peritraumatic Dissociative Experience Questionnaire (PDEQ). All three studies identified a trend showing a beneficial effect of family involvement interventions on severity of trauma through reduced severity scores, ranging from a reduction of -3 to -7 points on the various scales, however none of these results reached statistical significance.

Patient outcomes (Table 11): Three kinds of patient outcomes were reported in three studies involving multicomponent-, web- and discussion-based interventions: patient mortality[22,31] adverse events[19] and length of stay[19,31]. A statistically significant increase in mortality was reported in two studies; specifically one study found an actual percentage increase in the intervention group of 31% (RR: 4.91, CI: 1.55 to 15.51, p<0.05)[22] while the other of 7.5% (RR: 1.27, CI: 1.09 to 1.48, p=0.008)[31]. A relative reduction in adverse events per 1000 patient days of 29% was also reported[9] with an actual mean difference of -17 (CI: 6.95 to 27.05, p<0.05). Length of stay was examined in two studies with conflicting results: one reported a statistically significant reduction (MD: -0.77, CI: -0.69 to -0.87, p<0.001)[31] while another did not find any statistically significant difference[19].

Qualitative results

Features that influence effectiveness

The findings from the qualitative studies (n=5) were analysed thematically to identify features of the interventions that participants perceived to influence effectiveness. These included accessibility, simplicity, supplementarity, contextualisation, interprofessionality, consistency, relationship and confidence development. Synthesised into five overarching categories, these can serve as principles to inform the future design and development of more refined family member involvement interventions in ICU: a) Practicality, b) Development, c) Interaction, d) Reflexivity and e) Bridging. We present a high-level summary of these below, with a more detailed qualitative report to be made available in a forthcoming publication.

Practicality: Identified as a theme in three studies[20,23-24] this included intervention accessibility, simplicity, long term application and repetition. Practicality points to effective interventions needing to be readily available with clear instructions, which are as close to universally identifiable as possible. This may enable the intervention to be used as many times as necessary, benefiting family members over and beyond an individual encounter.

Development: In four studies[17,20,24,30] developing family members' contextual knowledge, or insight, was particularly valuable for effective intervention delivery. Insight was key to developing shared understanding among all those engaged in the treatment process. Insight should go beyond an understanding of the patient's condition to encompass the

For peer review only - http://bmjopen.bmj.com/site/about/guidelines.xhtml

diverse perspectives of healthcare professionals and the emotional strain which family members naturally undergo.

Interaction: Three studies[17,20,24] noted that interventions, either explicitly or implicitly, should encourage verbal and physical interaction between clinicians and family members. However, interprofessional discussions among ICU clinicians, and specifically between medical and nursing staff, appear equally important. Interventions that foster quality interaction at all levels appear essential for the family to be coherently integrated into the structure of the ICU.

Reflexivity: While effective interventions bring people together, three studies[17,23-24] noted they should also benefit the individual family member, who has the opportunity for personal growth in the path towards meaningful family member integration. Specific examples of reflexivity in the featured interventions were identified in the confirmatory exchanges between family members and healthcare professionals, as family members were able to explore their own role within the ICU, the relationships which they had independently developed with their clinicians and how they could utilise this unique knowledge as a form of leverage in their communication with ICU professionals.

Bridging: Effective interventions should also provide key and otherwise unavailable pathways to encourage family member integration. In two studies[20,23] the supplementary capacity which effective interventions offer was of particular value when consistent or predictable communication could not be guaranteed. While information availability is important, acknowledging the many difficulties which family members themselves face, and offering ways to overcome these, may ultimately enhance integration and experience.

Proposed typology

Utilising the learning gained from the current review, and the insights of our service users and carers group, we propose a typology of interventions (Figure 2) to be considered when promoting family member involvement in ICU. Presented in a matrix, we position types of interventions along a continuum of low to high involvement (environmental unit changes, web-based support, discussion-based support, multicomponent support, participation in rounds and participation in physical care) along with their key characteristics (type of ICU, family time commitment, professional input, cost, opportunities & challenges), level of evidence (low, moderate, high) and impact on key involvement and health outcomes (satisfaction, decision making, communication, family wellbeing, family trauma, and patient outcomes). We completed an initial face and content validation with our project's service users group consisting of ICU survivors and family members, and advisory board consisting of ICU professionals, but would still caution that the typology remains in need of further empirical validation in different ICU settings.

For peer review only - http://bmjopen.bmj.com/site/about/guidelines.xhtml

DISCUSSION

The diverse nature of the family involvement interventions we identified, the heterogeneity of research designs found, and the varying methodological quality of available studies meant there was not a single intervention that stood out as the recommended way forward. Overall, the available evidence suggests that: rounds-based interventions can benefit communication outcomes; multi-component and web-based interventions can increase family satisfaction; and, discussion-based interventions can benefit decision making and family wellbeing. However, interventions can be of varying cost, require different kinds and levels of input and time commitment from clinicians and family members, and impact on different kinds of outcomes. Given the current state of the evidence, we discourage proposing a single intervention as the 'gold standard' and instead encourage use of our typology to inform decisions based on individual ICU teams' context, needs and available resources.

At this stage, we suggest our typology is used as a reflective tool to aid ICU teams make evidence-informed decisions about potential interventions they may wish to consider adopting locally. In the first instance, teams are advised to discuss the kinds of engagement and/or clinical outcomes their unit wants to improve as a priority so they can focus and limit their choice of intervention types. They could then consider the evidence level, cost and key opportunities and challenges offered by each intervention type to help them make fully informed decisions as well as manage expectations. Finally, they could consider the time investment expected of ICU professionals and of family members for the different intervention types; for example, in geographically remote areas where family visiting is a challenge and staff shortage is significant, the team may favour trialling an intervention with less time commitment.

Through focussing on the different types of available interventions, their associated involvement and clinical outcomes, features perceived to influence effectiveness, and proposing an overarching typology of family involvement interventions, the current review makes a significant contribution to the available literature. Past review work in this area largely focussed on interventions that can support family members of critically ill patients[36-39]; these included providing care and treatment options for family members to counter the negative effects arising out of a loved one being hospitalised, or dying, in ICU. Available guidelines often suggest a long list of initiatives to support family members without full consideration or instruction on the contextual requirements of these suggestions or the kind of impact likely to be achieved by these. While prior reviews concluded, as have we, that the evidence in this field needs development and strengthening, these did not offer a granulated analysis of the evidence level for different kinds of interventions or presented these in a visual and user-friendly way to inform future work. Such work has helped spark the current movement around family involvement in ICU but remains somewhat limiting in that it does not adequately distinguish between family *support* and family *involvement*, often conflating the two. Consequently, conclusions of past work lack clarity on what can be done to involve as well as support family members.

For peer review only - http://bmjopen.bmj.com/site/about/guidelines.xhtml

We expect our typology to introduce some much-needed clarity in this field of research, as well as inform the development of future interventions and research studies. Importantly, the typology may be used by individual ICUs to inform discussions among professionals, and with family members, about which kinds of family involvement interventions they may wish to adopt, trial, and implement in their units.

We add to this field of research by adopting strict methodological standards in the current review, to reduce risk of reviewer bias and strengthen transparency. Many of the available guidelines for family involvement in ICU are based on a scoping review methodology, which can be methodologically limiting especially in lacking independent and duplicate screening, data extraction and quality assessment; though with notable exceptions[40]. A further way in which we sought to build on others' work was by examining the full spectrum of involvement interventions, rather than focussing on singular aspects such as decision making. Moreover, we opted to include service users throughout the review process, rather than limiting their involvement to the final stages of peer checking results. This allowed our service users to contribute throughout the process, see how the review was developing, appreciate the technical side of the review process and of the available evidence, and importantly to contribute actively in the development of the final typology.

The involvement of a service users and carers group led to us to identify a key, fundamental flaw of existing family involvement interventions and research: in our assessment, these tend to treat family members as a homogeneous group of individuals, rather than as heterogenous units. Specifically, different family members may have different involvement needs and preferences influenced by their previous experiences, knowledge, mental and physical health, and socioeconomic state. In addition, the notion of a family member extends beyond the 'next of kin' or 'personal consultee' to encompass extended family, carers and friends. Moreover, our service users emphasised that the dynamics within family units are crucial to shaping the experience, benefit and satisfaction of family members with ICU care and their level of involvement.

Current family involvement interventions and initiatives appear to be applied in ICUs following a blanket approach, without adequate consideration of particular family members' needs and preparedness for involvement. This may result in some family members benefiting while others experiencing harm, which goes some way in explaining the wide confidence intervals identified in our review that often cross the line of no difference. Indeed, within the PTSD literature, systematic reviews have long concluded that interventions applied universally to victims of trauma, such as compulsory debriefing, should cease and be replaced with a 'screen and treat' model[41]. We therefore invite researchers and clinicians to consider the aforementioned issues carefully in future initiatives for involving family members in ICU.

The current review should be considered in the context of its limitations. These include lack of a meta-analysis, and research published in languages other than English and from lower- and middle-income countries.

For peer review only - http://bmjopen.bmj.com/site/about/guidelines.xhtml

CONCLUSION

We examined interventions for family member involvement in ICUs, assessed the quality level of the available evidence, presented key results grouped by kinds of outcomes and interventions, and identified key features stakeholders perceived to influence intervention success. We summarised our results in the form of a typology, which we offer here as a first step towards developing a more systematic programme of research on interventions for family involvement in ICU. Based on the learning gained from the current review, we argue that future interventions should be developed with much closer family member input and designed by considering the key success features we identified. Importantly, we call for future interventions to be multi-layered to allow for a greater or lesser level, and different kind of, involvement for family members; the decision of which should be informed by a baseline diagnostic of family members' readiness and preparedness for involvement.

DECLARATIONS

Acknowledgement

We are grateful to our Expert Advisory Board and patient advisers, members of our Service Users & Carers Group, for their invaluable insight and support throughout this review. We owe intellectual debt to the late Professor Scott Reeves who generously supervised this project until his untimely passing in May 2018.

Ethics approval and consent to participate – Not applicable.

Consent for publication – Not applicable.

Availability of data and material – The datasets generated and/or analysed during the current study are available from the corresponding author on reasonable request.

Competing interests – The authors declare that they have no competing interests.

Funding – This paper presents independent research funded by the National Institute for Health Research (NIHR) under its Research for Patient Benefit (RfPB) Programme (Grant Reference Number PB-PG-0416-20021). The views expressed are those of the authors and not necessarily those of the NHS, the NIHR or the Department of Health and Social Care.

Author contributions – AX was responsible for conceiving the review, developing the review question and methodology, leading the funding application and coordinating the project. SF and JP were responsible for developing and executing the search strategy. AX, SF and JP were responsible for reference screening, quality assessment, data extraction and management. AMR, MT and SB gave advice throughout the project and contributed to data interpretation. AX and SF were responsible for the statistical inferences and qualitative synthesis respectively. SF and AX led the development of the first draft of the manuscript. SB, MT and AMR gave editorial comments on the draft manuscript. AX is the guarantor of the review. AX, SF, JP, SB, MT, and AMR read and approved the manuscript.

For peer review only

Figure 1: Flow diagram

Figure 2: Typology of family involvement interventions

REFERENCES

1 Institute of Medicine. To err is human New York: National Academies Press; 2001.

2 Wanless D. Securing our Future Health: Taking a Long-Term View London: HM Treasury; 2002.

3 NHS England. Transforming Participation in Health and Care: Guidance to support commissioners. London; 2013.

4 NHS England. Five Year Forward View. London; 2015.

5 Wright S, Walmsley E, Harvey S, Robinson E, Ferrando-Vivas P, Harrison D, et al. Family-Reported Experiences Evaluation (FREE) study: a mixed-methods study to evaluate families' satisfaction with adult critical care services in the NHS. Health Services and Delivery Research. 2015; 3: p. 45.

6 Azoulay E, Pochard F, Kentish-Barnes N, Chevret S, Aboab J, Adrie C, et al. Risk of post-traumatic stress symptoms in family members of intensive care unit patients. American Journal of Respiratory and Critical Care Medicine. 2005;: p. 987-994.

7 Azoulay E, Chaize M, Kentish-Barnes N. Involvement of ICU families in decisions: fine-tuning the partnership. Annals of Intensive Care. 2014; 4: p. 1.

8 Bench S, Cornish J, Xyrichis A. A qualitative exploration of Intensive Care discharge summaries for General Practice staff. British Journal of General Practice. 2016; 66(653): p. e904-e912.

9 Davidson JE, Powers K, Hedayat KM, Tieszen M, Kon AA, Shepard E, et al. Clinical practice guidelines for support of the family in the patient-centered intensive care unit: American College of Critical Care Medicine Task Force 2004–2005. Critical Care Medicine. 2007; 35(2): p. 605-622.

10 Olding M, McMillan SE, Reeves S, Schmitt MH, Puntillo K, & Kitto S. Patient and family involvement in adult critical and intensive care settings: a scoping review. Health Expectations. 2015; 19: p. 1183-1202.

11 Liput SA, Kane-Gill SL, Seybert AL, Smithburger PL. A Review of the Perceptions of Healthcare Providers and Family Members Toward Family Involvement in Active Adult Patient Care in the ICU. Critical Care Medicine. 2016; 44(6): p. 1191-1197.

12 Reeves S, Fletcher S, Barr H, Birch I, Boet S, Davies N, et al. A BEME systematic review of the effects of interprofessional education: BEME Guide No. 39. Medical Teacher. 2016; 38(7): p. 656-668.

13 Xyrichis A, Fletcher S, Brearley S, Philippou J, Purssell E, Terblanche M, Rafferty AM, Reeves S. Interventions to promote patients and families' involvement in adult intensive care settings: a protocol for a mixed-method systematic review. Systematic Reviews. 2019; 8(1): p. 185-192.

For peer review only - http://bmjopen.bmj.com/site/about/guidelines.xhtml

14 Dufour É, Duhoux A, Bolduc J. Measured and perceived effects of audit and feedback on nursing performance: a mixed methods systematic review protocol. Systematic Reviews. 2019; 8(1): p. 38.

15 Carroll C, Booth A, Leaviss J, Rick J. “Best fit” framework synthesis: refining the method. Medical Research Methodology. 2013; 13(1): p. 37.

16 Choi YS, Bosch SJ. Environmental affordances: Designing for family presence and involvement in patient care. Health Environments Research & Design Journal. 2013; 6(4): p. 53-75.

17 Rippin AS, Zimring C, Samuels O, Denham ME. Finding a Middle Ground: Exploring the Impact of Patient-and Family-Centered Design on Nurse–Family Interactions in the Neuro ICU. Health Environments Research & Design Journal. 2015; 9(1): p. 80-98.

18 Dalal AK, Dykes PC, Collins S, Lehmann LS, Ohashi K, Rozenblum R, et al. A web-based, patient-centered toolkit to engage patients and caregivers in the acute care setting: a preliminary evaluation. Journal of the American Medical Informatics Association. 2015; 23(1): p. 80-87.

19 Dykes PC, Rozenblum R, Dalal A, Massaro A, Chang F, Clements M, et al. Prospective evaluation of a multifaceted intervention to improve outcomes in intensive care: the Promoting Respect and Ongoing Safety Through Patient Engagement Communication and Technology Study. Critical Care Medicine. 2017; 45(8): p. e806-e813.

20 Ernecoff NC, Witteman HO, Chon K, Buddadhumaruk P, Chiarchiaro J, Shotsberger KJ, et al. Key stakeholders' perceptions of the acceptability and usefulness of a tablet-based tool to improve communication and shared decision making in ICUs. Journal of Critical Care. 2016; 33: p. 19-25.

21 Huffines M, Johnson KL, Naranjo LLS, Lissauer ME, Fishel MAM, Howes SMA, et al. Improving family satisfaction and participation in decision making in an intensive care unit. Critical Care Nurse. 2013; 33(5): p. 56-69.

22 Almoosa KF, Goldenhar LM, Panos RJ. Characteristics of discussions on cardiopulmonary resuscitation between physicians and surrogates of critically ill patients. Journal of Critical Care. 2009; 24(2): p. 280-287.

23 Garrouste-Orgeas M, Max A, Lerin T, Grégoire C, Ruckly S, Kloeckner M, et al. Impact of proactive nurse participation in ICU family conferences: a mixed-method study. Critical Care Medicine. 2016; 44(6): p. 1116-1128.

24 Hollman Frisman G, Wåhlin I, Orvelius L, Ågren S. Health-promoting conversations - a novel approach to families experiencing critical illness in the ICU environment. Journal of Clinical Nursing. 2018; 27(3): p. 631-639.

25 Huang KB, Weber U, Johnson J, Anderson N, Knies AK, Nhundu B, et al. Primary Care Physician Involvement in Shared Decision Making for Critically Ill Patients and Family Satisfaction with Care. The Journal of the American Board of Family Medicine. 2018; 31(1): p. 64-72.

26 Curtis JR, Treece PD, Nielsen EL, Gold J, Ciechanowski PS, Shannon SE, et al. Randomized trial of communication facilitators to reduce family distress and intensity of end-of-life

For peer review only

care. American Journal of Respiratory and Critical Care Medicine. 2016; 193(2): p. 154-162.

27 Shaw DJ, Davidson JE, Smilde RI, Sondoozi T, Agan D. Multidisciplinary team training to enhance family communication in the ICU. Critical Care Medicine. 2014; 42(2): p. 265-271.

28 Cray L. A collaborative project: initiating a family intervention program in a medical intensive care unit. Focus on Critical Care. 1989; 16(3): p. 213-218.

29 Davidson JE, Daly BJ, Agan D, Brady NR, Higgins PA. Facilitated sensemaking: a feasibility study for the provision of a family support program in the intensive care unit. Critical Care Nursing Quarterly. 2010; 33(2): p. 177-189.

30 Marshall AP, Wake E, Weisbrodt L, Dhaliwal R, Spencer A, Heyland DK. A multi-faceted, family-centred nutrition intervention to optimise nutrition intake of critically ill patients: The OPTICS feasibility study. Australian Critical Care. 2016; 29(2): p. 68-76.

31 White DB, Angus DC, Shields AM, Buddadhumaruk P, Pidro C, Paner C, et al. A Randomized Trial of a Family-Support Intervention in Intensive Care Units. New England Journal of Medicine. 2018; 378(25): p. 2365-2375.

32 Allen SR, Pascual J, Martin N, Reilly P, Luckianow G, Datner E, et al. A novel method of optimizing patient-and family-centered care in the ICU. Journal of Trauma and Acute Care Surgery. 2017; 82(3): p. 582-586.

33 Jacobowski NL, Girard TD, Mulder JA, Ely EW. Communication in critical care: family rounds in the intensive care unit. American Journal of Critical Care. 2010; 19(5): p. 421-430.

34 Weber U, Johnson J, Anderson N, Knies AK, Nhundu B, Bautista C, et al. Dedicated Afternoon Rounds for ICU Patients' Families and Family Satisfaction With Care. Critical Care Medicine. 2018; 46(4): p. 602-611.

35 Prichard C, Newcomb P. Benefit to family members of delivering hand massage with essential oils to critically ill patients. American Journal of Critical Care. 2015; 24(5): p. 446-449.

36 Davidson JE, Powers K, Hedayat KM, Tieszen M, Kon AA, Shepard E, Spuhler V, Todres ID, Levy M, Barr J, Ghandi R. Clinical practice guidelines for support of the family in the patient-centered intensive care unit: American College of Critical Care Medicine Task Force 2004–2005. Critical care medicine. 2007 Feb 1;35(2):605-22.

37 Davidson JE, Aslakson RA, Long AC, Puntillo KA, Kross EK, Hart J, Cox CE, Wunsch H, Wickline MA, Nunnally ME, Netzer G. Guidelines for family-centered care in the neonatal, pediatric, and adult ICU. Critical care medicine. 2017 Jan 1;45(1):103-28.

38 Patient- and Family-Centered Care Initiatives in Acute Care Settings: A Review of the Clinical Evidence, Safety and Guidelines [Internet]. Ottawa, Canada: Canadian Agency for Drugs and Technologies in Health; 2015. http://www.ncbi.nlm.nih.gov/books/NBK350000/. Accessed September 14, 2016.

For peer review only - http://bmjopen.bmj.com/site/about/guidelines.xhtml

39 Singer AE, Ash T, Ochotorena C, Lorenz KA, Chong K, Shreve ST, Ahluwalia SC. A systematic review of family meeting tools in palliative and intensive care settings. American Journal of Hospice and Palliative Medicine®. 2016 Sep;33(8):797-806.

40 Bibas L, Peretz-Larochelle M, Adhikari NK, Goldfarb MJ, Luk A, Englesakis M, Detsky ME, Lawler PR. Association of surrogate decision-making interventions for critically ill adults with patient, family, and resource use outcomes: a systematic review and meta-analysis. JAMA network open. 2019 Jul 3;2(7):e197229.

41 Rose SC, Bisson J, Churchill R, Wessely S. Psychological debriefing for preventing post traumatic stress disorder (PTSD). Cochrane database of systematic reviews. 2002(2).

For peer review only

List of Tables

Table 1: Included studies 19
Table 2: Intervention characteristics....................21
Table 3: Quality assessment - Quantitative studies 28
Table 4: Quality assessment - Qualitative studies....................28
Table 5: Communication outcome measures 29
Table 6: Decision making outcome measures....................29
Table 7: Satisfaction outcome measures 30
Table 8: Anxiety outcome measures....................31
Table 9: Depression outcome measures....................31
Table 10: Trauma outcome measures....................32
Table 11: Patient outcome measures 32

For peer review only

For peer review only - http://bmjopen.bmj.com/site/about/guidelines.xhtml

Table 1: Included studies

Author	Year	Country and setting	Design	Sample size	Research approach	Intervention
Allen et al	2017	USA – Surgical ICU	Non-randomised, before and after study	Patients: n=847<br>Family members: n=429	Quantitative	Engaging family members on rounds
Almoosa et al	2009	USA – Medical ICU	Prospective, 2-centre observational study	Patients: n=85<br>Family members: n=85	Quantitative	CPR Discussions
Choi and Bosch	2013	USA – Neuro ICU and Trauma ICU	Comparative observational study	Patients: n=81<br>Family members: n=81	Quantitative	Patient and family centred unit design
Cray	1989	USA – Medical ICU	Post hoc evaluation study	Patients: n=76<br>Family members: n=76	Quantitative	Family intervention programme
Dalal et al	2015	USA – Medical ICU (Oncology Unit)	Post hoc evaluation study	Patients: n=26<br>Family members: n=77	Quantitative	Patient centred toolkit
Davidson et al	2010	USA – Mixed ICU	Feasibility study		Quantitative	Family Support Programme, based on Facilitated Sensemaking
Dykes et al	2017	USA – Medical ICU	Non-randomised, before and after study	Patients: n=58<br>Family members: n=156	Quantitative	Patient Engagement Communication and Technology (PROSPECT)
Ernecoff et al	2016	USA – Medical ICU	Qualitative interview study	Family members: n=30<br>Staff members: n=28	Qualitative	Tablet-based support tool
Garrouste-Orgeas et al	2016	France – Surgical ICU	Randomised controlled trial with nested qualitative study	Patients: n=100<br>Family members: n=88	Mixed methods	Proactive participation of a nurse in family conferences
Hollman Frisman et al	2018	Sweden – ICU	Qualitative interview study	Patients: n=8<br>Family members: n=10	Qualitative	Health promoting conversations
Huang et al	2018	USA – Neuroscience ICU	Prospective, single-centred observational study	Family members: n=263	Quantitative	Primary care physician involvement in decision-making in the ICU
Huffines et al	2013	USA – Surgical ICU	Non-randomised, before and after study	Family members: n=48	Quantitative	SICU Family supportive care algorithm
Marshall et al	2016	Australia – General ICU	Feasibility study	Family members: n=51	Qualitative	Multi-faceted family-centred nutrition intervention
Jacobowski et al	2010	USA – Medical ICU	Non-randomised, before and after study	Family members: n=111	Quantitative	Family rounds
Prichard and Newcomb	2015	USA – Trauma ICU	Quasi experimental pilot study	Family members: n=30	Quantitative	Hand massage
Randall-Curtis et al	2016	USA – General ICU	Randomised controlled trial	Patients: n=168<br>Family members: n=268	Quantitative	Communication facilitator

For peer review only

Author	Year	Country and setting	Design	Sample size	Research approach	Intervention
Rippin et al	2015	USA – Neuroscience ICU	Comparative observational study	Family members: n=54<br>Staff members: n=18	Qualitative	Family centred unit design
Shaw et al	2014	USA – General ICU	Non-randomised, before and after study	Patients: n=121<br>Family members: n=121	Quantitative	Multidisciplinary team training to enhance family communication in the ICU
Weber et al	2018	USA – Neuroscience ICU	Non-randomised, before and after implementation study	Family members: n=141	Quantitative	Family rounds
White et al	2018	USA - 2 Neuro ICU, 2 Mixed ICU, 1 Medical ICU	Multicentre, stepped-wedge, cluster-randomized	Patients: n= 1420<br>Family members: n= 1106	Quantitative	Multicomponent family-support intervention

For peer review only

Table 2: Intervention characteristics

Author	Activity Type	Intervention	Purpose	Procedure	Participants	Beneficiary	Outcomes
Allen et al. 2017	Rounds based	Family member involvement in rounds	Engaging and integrating ICU patients' family members and surrogates on daily rounds	On admission to the ICU, a family member was invited to participate on daily rounds with the critical care team.	Family members, nurses, physicians	Family members, nurses, physicians	• significantly increased family member knowledge<br>• strengthened relationship between family members and doctors<br>• nurses reported: greater enjoyment<br>• improved communication<br>• reduction in workload<br>• increased support for patient and family centered care.<br>• increased physician satisfaction post intervention
Almoosa et al. 2009	Discussion based	CPR Discussions	The implementation of structured, informed CPR specific conversations with family members and surrogates from relevant physicians	Physician initiated CPR conversation about: chest compressions, electrical cardioversion, mechanical ventilation	Physicians, residents, fellows or family	Family members	• satisfaction with CPR discussions was higher after the intervention
Choi and Bosch 2013	Unit design	Patient and family centred unit design	Unit redesign undertaken to enable physical space to more broadly facilitate family interaction with patient and family interaction with staff	The intervention unit included larger spaces and more comfortable accommodations for visitors	Family members	Family members and patients	• increased family interactions with patient<br>• increased family interactions with staff
Cray et al. 1989	Multi-component	Family intervention programme	Multi-component intervention to enhance family involvement in and understanding of their loved one's condition in ICU	Intervention components included: Family conference, Telephone conversation, Visit by nurse, Follow up visit to the intermediary care unit	Family members, Clinical nurse specialists	Family members	• strong agreement that the intervention helped family members to understand their loved one's illness and benefit other families.<br>• high satisfaction with intervention components.

For peer review only - http://bmjopen.bmj.com/site/about/guidelines.xhtml

Author	Activity Type	Intervention	Purpose	Procedure	Participants	Beneficiary	Outcomes
Dalal et al. 2016	Web based/ electronic	Patient centred toolkit (PCTK)	The PCTK was a suite of web-based patient-facing and provider-facing tools designed to facilitate collaborative decision making by providing access to tailored educational content and facilitating patient-provider communication	The PCTK was accessible by patients, caregivers, and providers from any web-enabled device connected to the hospital's secure intranet.	Patients and caregivers	Patients and family members	• the majority of patients and caregivers surveyed were satisfied or extremely satisfied with the intervention
Davidson et al. 2010	Multi-component	Family Support Programme, based on Facilitated Sensemaking	A systems-based patient-centered care and engagement program	The intervention consisted of 2 main components: personalized instruction and provision of family visiting kits.	Family members, Clinical nurse specialists	Family members	• high rate of instrument reliability<br>• a breakdown of the top ten needs of family members<br>• a ranking of interventions' helpfulness<br>• identification of any additional needs
Dykes et al. 2017	Web based/ electronic	Promoting Respect and Ongoing Safety through Patient Engagement Communication and Technology (PROSPECT).	A systems-based patient-centered care and engagement program designed to enhance patient and care partner experience in the ICU	60 minute training session to facilitate patient-centred care in healthcare professionals,<br>A web based – toolkit to apply the training,<br>Delivery of the Patient SatisfActive Model from nurses to patients in ICU	Nurses and patients	Nurses and patients	• relative reduction in the rate of adverse effects<br>• improvements in patient satisfaction<br>• improvements in care partner satisfaction

For peer review only - http://bmjopen.bmj.com/site/about/guidelines.xhtml

Author	Activity Type	Intervention	Purpose	Procedure	Participants	Beneficiary	Outcomes
Ernecoff et al. 2016	Web based/ electronic	Tablet-based and video-driven communication and decision support tool	A tool was conceptualised to: 1) prepare the family for conversations with clinicians, 2)give clinicians tailored information about the family and patient in advance of the family meeting, 3) promote a personalized relationship between clinician and family, and 4) provide general decision support to surrogates.	The sections of the tool for surrogates were: 1) orienting surrogates to the ICU, 2) explaining principles of surrogate decision making 3) providing a question prompt list and opportunity to write down questions, 4) a values clarification exercise 5) education about treatment pathways (e.g., life prolonging treatment, comfort-focused treatment; and a time-limited trial of ICU care);6) eliciting surrogates prognostic information, and 7) providing psycho-social resources	Family members (surrogates)	Family members	• enhancing and supplementing communication between surrogates and the clinical team<br>• leveraging surrogates' downtime before and between clinician-family meetings<br>• helping surrogates to consider the patient's values and treatment options<br>• allowing for repetition and review of information
Garrouste-Orgeas et al. 2016	Discussion based	Proactive participation of a nurse in family conferences	Integration of nursing staff in family conferences focussed around: Naming emotions, expressing Understanding, showing Respect, articulating Support for the patient, and Exploring the family's emotional state	All family members who wished to attend were escorted to the room. Briefly, an open question was asked first to encourage the family members to express themselves. A substantial proportion of the time was devoted to listening to the family, and the professionals used simple words to enhance comprehension. At the end of the conference, the family was allowed enough time to ask all the questions they had.	Physicians, nurses, family members	Family members	• family members reported that the conferences allowed them to receive and assimilate information<br>• be listened to regarding both their positive and negative feelings<br>• receive compassion and respect

For peer review only - http://bmjopen.bmj.com/site/about/guidelines.xhtml

Author	Activity Type	Intervention	Purpose	Procedure	Participants	Beneficiary	Outcomes
Hollman Frisman et al. 2018	Discussion based	(Nurse led) Health promoting conversations with families	The aim of the conversations was to create a context for change related to the families' problems and resources.	Discussion of the aim of the conversation series and the families' and nurses' expectations about the conversations and each other's roles. The three conversation sessions focused on topics that the families considered important, and the dialogue and questioning intended to identify resources within and outside the family. At the end of each conversation, the nurse offered a short reflection on how the family members had experienced the session. A closing letter was sent to the family 2–3 weeks after the last conversation to summarise further possibilities for reflection.	Nurses and family members	Family members	• health promoting conversations led to increasing emotional openness<br>• enhanced consciousness regarding illness<br>• greater family member satisfaction<br>• a valuable sense of confirmation<br>• general promotion of family wellbeing
Huang et al. 2018	Discussion based	Primary Care Physician involvement in decision-making in the ICU	The study evaluated survey results which measured family member satisfaction with general ICU care and shared decision making between Primary Care Physicians and family members	Examined involvement of the patient's Primary Care Physician and shared decision making.	Primary care physicians	Family members	• a higher proportion reporting PCP involvement felt completely satisfied with their inclusion in the ICU decision making process

For peer review only - http://bmjopen.bmj.com/site/about/guidelines.xhtml

Author	Activity Type	Intervention	Purpose	Procedure	Participants	Beneficiary	Outcomes
Huffines et al. 2013	Web based/ electronic	SICU Family Supportive Care Algorithm	The goal of the interventions in the 24-hour bundle is to inform families about the importance of their participation in decision making and to inform them of the resources available to help them participate in decision making.	Within 24 hours of admission, a member of the intensivist team meets with the family. Also within the first 24 hours after a patient's SICU admission, the patient's family is encouraged to watch an on-demand 10-minute video.<br>72 hours:<br>Reaffirm and ensure family is supported. Encouraged to participate in decision making in rounds.<br>96 hours:<br>Implement a family meeting with the multidisciplinary team.	Bedside nurses	Family members	• mixed to positive outcomes when measuring family satisfaction<br>• staff teamwork<br>• participation in decision making<br>• frequency of support
Marshall et al. 2016	Multi-component	Multi-faceted, family-centred nutrition intervention	To assist families in discussing nutritional goals with health professionals	Consisted of:<br>Family interviews,<br>Focussed education sessions, and Nutrition diaries	Dieticians, Family Nurses	Families and patients	• perceptions around how nutrition education can be improved<br>• the diversity of experience relating to the provision of in-hospital nutrition therapy<br>• continuity of existing family member involvement approaches<br>• the importance of families as advocates
Jacobowski et al. 2010	Rounds based	Family rounds	To enhance communication between medical and nursing staff and the families of ICU patients	The attending physician provided a summary for the family using understandable, lay language and the family was offered an opportunity to ask questions of the team	Physicians, nurses, family members	Physicians, nurses, family members	• communication regarding condition improved significantly as did decision making support.<br>• there was a decline in the number of family members who thought that they had sufficient time to address their questions and concerns.

For peer review only - http://bmjopen.bmj.com/site/about/guidelines.xhtml

Author	Activity Type	Intervention	Purpose	Procedure	Participants	Beneficiary	Outcomes
Prichard and Newcomb 2015	Physical	Hand massage	To provide physical relief to patients in the ICU through hand massage	Participants were taught to administer hand massage in compliance with the M Technique, a registered method of simple, structured touch that has been used on critically ill patients with positive effects. For this study, the technique was used on hands. Participants applied the intervention twice daily for 5 minutes per session for 3 consecutive days	Family members, patients	Family members, patients	• anxiety was greatly reduced in the treatment group
Randall-Curtis et al. 2016	Discussion based	Communication-facilitator	To understand the family's concerns, needs, and communication characteristics	Consisted of facilitated: Interviews, Meetings, Communication and emotional support; as well as Facilitator participation in family conferences and 24 hour follow up	Nurse or social worker trained to improve communication between the ICU team and the family by acting as a communication facilitator or navigator	Family members	• adjusted depression scores were lowered alongside ICU costs
Rippin et al. 2015	Unit design	Family Centred Unit design	Unit designed to prioritise and engage the family in the ICU context	The FCU physically integrated family into the fabric of the unit. Nurses worked between centralized nursing stations and decentralized alcoves just outside patient rooms for improved monitoring and safety. Rooms were larger (245 sq ft) with more space around the bedside.	Nurses and family members	Family members	• reduction in problematic bedside co-presence (or overcrowding) • a continuity of pre intervention distribution patterns in which nurse and family remained clustered in their respective 'domains' • nurse perspectives which acknowledged the unpredictability and complexity of the ICU and the subsequent mixed results which this newly implemented unit design encouraged

For peer review only - http://bmjopen.bmj.com/site/about/guidelines.xhtml

Author	Activity Type	Intervention	Purpose	Procedure	Participants	Beneficiary	Outcomes
Shaw et al. 2014	Discussion based	Multidisciplinary team training to enhance family communication in the ICU	The training material was selected to improve communication. The training was also designed to foster team building and improve collaborative relationships among clinicians from multiple disciplines.	Training included how to conduct and participate in patient/family conferences, addressing goals of care and giving critical information in the intensive care setting so that all caregivers might speak to patients and their families with a common voice. The training was designed to address known drivers of family satisfaction, as well as to address each of the 21 items being measured in the staff confidence survey.	ICU team members	MDT team members and family members	• staff confidence and family satisfaction were shown to significantly improve
Weber et al. 2018	Rounds based	Family Rounds	To improve family satisfaction in ICU experience by integrating them into rounds processes	After each session, the nursing leader recorded (1) whether family rounds occurred (2), how many families participated, and (3) how many patients the NICU team had rounded on that morning	Nursing staff	Family members	• family reported improved satisfaction with decision making, frequency of communication, receiving emotional support and with coordination of care<br>• percentage improvements in satisfaction scores were not large enough to reach statistical significance
White et al. 2018	Multi-component	Multicomponent family-support intervention	Collaborative intervention between nursing staff and family members designed to support caregivers in the ICU	Nurses received advanced communication training. A family support pathway was instituted. Intensive support for implementation was provided to each ICU by a quality-improvement specialist to incorporate the family-support pathway into clinicians' workflow.	Nursing staff, ICU team, family members	Family members and nursing staff	• significant improvements were noted with family satisfaction regarding:<br>• the quality of communication with clinicians, and<br>• perceptions of patient centeredness<br>• the intervention led to an average reduction in the length of stay in the ICU of 3 days

For peer review only

Table 3: Quality assessment - Quantitative studies

Study Id	Aim of Evaluation	Research Design	Sample size	Power calculation	Number of groups	Method of allocation	Allocation concealment	Blinding	Loss to follow up	Data collection method	Significance measures	Data analysis method	Reported biases or confounders	Relevance	Ethics	Strength of design	Strength of numbers	Quality of information	Quality of study	Overall weighting
Allen 2017	+	-	+	-	-	-	-	-	-	-	+	+	+	+	+	?	?	?	?	Low
Almoosa 2009	+	+	+	+	+	-	-	-	+	+	+	+	+	+	+	?	?	?	?	Moderate
Choi 2013	+	-	+	-	+	-	-	-	-	-	+	+	+	+	-	?	?	?	?	Low
Cray 1989	+	-	?	?	?	?	?	?	?	?	+	+	-	+	?	-	+	?	-	Low
Dalal 2016	-	-	+	-	-	-	-	-	-	-	+	+	+	+	+	?	?	?	?	Very low
Davidson 2010	+	+	+	-	+	-	-	-	+	+	+	+	+	+	+	?	?	?	?	Moderate
Dykes 2017	+	-	+	+	-	-	-	-	-	+	+	+	+	+	+	?	?	?	?	Low
Garrouste-Orgeas 2016	+	+	+	+	+	+	+	-	+	+	+	+	+	+	+	+	+	+	+	Very high
Huang 2018	+	+	+	+	+	-	-	-	-	+	+	+	+	+	+	-	+	+	+	Moderate
Huffines 2013	+	+	+	-	+	-	-	-	+	+	+	+	+	+	?	?	?	?	?	Moderate
Jacobowski 2010	+	?	+	?	+	?	?	?	?	+	+	+	+	+	+	?	+	?	?	Moderate
Marshall 2016	+	+	+	-	?	-	-	-	-	+	+	+	+	+	+	+	+	?	+	Moderate
Prichard 2015	+	?	+	?	+	-	?	?	+	+	+	+	+	+	+	?	+	+	?	High
Randall-Curtis 2016	+	+	+	+	+	+	+	?	-	+	+	+	+	+	+	+	+	+	+	Very high
Shaw 2014	+	?	+	+	+	?	?	?	-	+	+	+	+	+	+	?	+	+	?	High
Weber 2018	+	+	+	+	+	?	?	?	?	+	+	+	+	+	+	?	?	?	?	High
White 2018	+	+	+	+	+	+	+	?	+	+	+	+	+	+	+	+	+	+	+	Very high

+ Low concern, - High concern, ? Unclear. Quality assessment tool elaborated in Xyrichis et al.[13]

Table 4: Quality assessment - Qualitative studies

Study Id	Aim of Evaluation	Sampling	Data collection	Data analysis	Research relations	Findings	Transferability	Relevance & Usefulness	Ethics	Quality of information	Quality of study	Overall weighting
Ernecoff 2016	+	+	+	+	-	+	+	+	+	+	+	High
Frisman 2018	+	+	+	+	-	+	+	+	+	+	+	High
Garrouste-Orgeas 2016	+	+	+	+	-	+	+	+	+	+	+	High
Marshall 2016	+	+	+	+	-	+	?	+	+	?	?	Moderate
Rippin 2015	+	?	+	+	?	+	+	+	-	?	?	Low

+ Low concern, - High concern, ? Unclear. Quality assessment tool elaborated in Xyrichis et al.[13]

For peer review only - http://bmjopen.bmj.com/site/about/guidelines.xhtml

Table 5: Communication outcome measures

Study / Intervention / Measure	Jacobowski et al. 2010	Weber et al. 2018	Shaw et al. 2014	White et al. 2018	Allen et al. 2017
**Intervention**	**Family rounds**	**Family rounds**	**Team training**	**Multi family support**	**Family on rounds**
**FS ICU 24 – Frequency of nurse communication**	% highest score<br>Pre: 64%<br>Post: 57%<br>**Change: -7% p=0.30**<br>**RR: 0.89 CI: 0.70-1.12**	% top scores<br>Control: 62%<br>Intervention: 78%<br>**Change: 16% p>0.05**<br>**RR: 1.24 CI 0.98-1.58**	Mean score<br>Pre: 79.2<br>Post: 87.18<br>**Change: 7.98 p=0.04***		
**FS ICU 24 – Frequency of doctor communication**	% highest score<br>Pre: 38%<br>Post: 60%<br>**Change: 22% p=0.004***<br>**RR: 1.60 CI 1.18-2.17**	% top scores<br>Control: 43%<br>Intervention: 56%<br>**Change: 13% p>0.05**<br>**RR: 1.31 CI 0.90-1.89**	Mean score<br>Pre: 67.86<br>Post: 76.69<br>**Change: 8.83 p=0.04***		
**FS ICU 24 – Honesty of information**			Mean score<br>Pre: 77.78<br>Post: 87.08<br>**Change: 9.30 p=0.01***		
**Quality of Communication (QOC)**				Mean score<br>Control: 62.7<br>Intervention: 69.1<br>**Change: 6.39 p=0.001***<br>**CI 2.57 – 10.20**	
**Communication improvement**					Pre: n=49 (100%)<br>Post: n=47 (100%)<br>**Change 0% p=0.68**<br>**RR: 1, CI: 0-0**
**Overall metric for comparison**	**RR: 1.60**	**RR: 1.31**	**MD: 8.83**	**MD: 6.39**	**RR: 1**

Table 6: Decision making outcome measures

Study / Intervention / Measure	Huang et al. 2018	Shaw et al. 2014	Weber et al. 2018	Jacobowski et al. 2010	Huffines et al. 2013	Almoosa et al. 2009
**Intervention**	**Primary physician involvement**	**Team training**	**Family rounds**	**Family rounds**	**Support care algorithm**	**CPR discussions**
**FS ICU 24 – Decision making**	Mean (sd)<br>Control: 80.07 (12.76)<br>Intervention: 81.06 (15.1)<br>**Change: 0.99 p=0.16**<br>**CI -3.00 – 4.98**	Mean score<br>Pre: 77.47<br>Post: 83.32<br>**Change: 5.85 p=0.05***	Mean score (SD)<br>Control: 85.1 (16.3)<br>Intervention: 88.6 (14.6)<br>**Change: 3.5 p=0.20**<br>**CI: -1.98-8.98**			
**Inclusion in decision making**	% completely satisfied<br>Control: 61.4%<br>Intervention: 75.9%<br>**Change: 14.5% p=0.05***<br>**RR: 1.23 CI 1.03-1.49**			% highest score<br>Pre: 66%<br>Post: 76%<br>**Change: 10% p=0.12**<br>**RR: 1.15 CI 0.96-1.38**		
**Control over patient care**	% completely satisfied<br>Control: 55.6%<br>Intervention: 73.6%<br>**Change: 18% p=0.02***<br>**RR: 1.31 CI 1.07-1.61**					
**Support in decision making**				% highest score<br>Pre: 49%<br>Post: 69%<br>**Change: 20% p=0.005***		

				RR: 1.39 CI 1.09-1.79		
**Share in decisions about care planning**					% scoring excellent<br>Pre: 45%<br>Post: 68%<br>**Change: 23% p=0.009***<br>**RR: 1.52 CI 1.03-2.24**	
**Time to decision**						Days mean (sd)<br>Control 7.4 (3.2)<br>Interv. 6.6 (6.5)<br>**Change -0.8 days CI -1.48-3.08**
**Overall metric for comparison**	**RR: 1.31**	**MD: 5.85**	**MD: 3.5**	**RR: 1.39**	**RR: 1.52**	**MD: -0.8**

Table 7: Satisfaction outcome measures

**Study**	**Huang et al. 2018**	**Shaw et al. 2014**	**Dykes et al. 2017**	**Weber et al. 2018**	**Huffines et al. 2013**
**Intervention / Measure**	**Primary physician involvement**	**Staff teamwork training**	**ICT-based engagement**	**Family rounds**	**Supportive care algorithm**
**FS ICU 24 – Global score**	Mean (sd)<br>Control: 84.91 (12.17)<br>Intervention: 86.4 (11.76)<br>**Change: 1.49 p=0.16**<br>**CI -2.14 - 5.12**	Mean score<br>Pre: 83.21<br>Post: 85.69<br>**Change: 2.48 p=0.32**	Mean score<br>Pre: 84.3 (3)<br>Post: 90 (1.9)<br>**Change: 5.7 p<0.05***<br>**CI 2.31-9.09**	Mean score (SD)<br>Control: 86.0 (16.0)<br>Intervention: 90.8 (10.7)<br>**Change: 4.8 p=0.20**<br>**CI: -0.12 - 9.72**	
**HCAHPS**			% top score 9-10<br>Pre: 71.8%<br>Post: 93.3%<br>**Change 21.5 P<0.05***<br>**RR 1.33 CI 1.10-1.55**		
**Support given**				% top scores<br>Control: 54%<br>Intervention: 71%<br>**Change: 17% p>0.05**<br>**RR: 1.32 CI 0.99-1.75**	% scoring excellent<br>Pre: 60%<br>Post 75%<br>**Change: 15% p=0.14**<br>**RR: 1.23 CI 0.91-1.65**
**Overall metric for comparison**	**MD: 1.49**	**MD: 2.48**	**RR: 1.33**	**RR: 1.32**	**RR: 1.23**

**Study**	**White et al. 2018**	**Cray 1989**	**Dalal et al. 2015**	**Jacobowski et al. 2010**
**Intervention / Measure**	**Multi-component family support programme**	**Family support programme**	**Patient centred toolkit**	**Family rounds**
**PPPC**	Mean score<br>Control: 1.8<br>Intervention: 1.7<br>**Change: -0.15 p=0.006***<br>**CI −0.26 - −0.04**			
**Satisfaction with intervention**		% Satisfied<br>**100% agreed (76/76)**	% Satisfied<br>**72% (13/18)**	
**Time to ask questions**				% highest score<br>Pre: 40%<br>Post: 23%<br>**Change: -17% p=0.02***<br>**RR: 0.57 CI 0.37-0.90**
**Overall metric for comparison**	**MD: 0.15**	**100%**	**72%**	**RR: 0.57**

For peer review only - http://bmjopen.bmj.com/site/about/guidelines.xhtml

Table 8: Anxiety outcome measures

Study / Intervention / Measure	Garrouste et al. 2016	Prichard & Newcomb 2015	White et al. 2018	Randall-Curtis et al. 2016
**Intervention**	**IP family conference**	**Hand massage**	**Multi family support**	**Communication facilitator**
**HADS - Anxiety**	90 days, Median [IQR]<br>Control: 8 [4.5-12]<br>Intervention: 4 [1-9]<br>**Change: -4 p=0.01***<br><br>% w\ Anxiety score > 8<br>Control: 52.3% (n=23)<br>Intervention: 33.3% (n=14)<br>**Change: 19% p=0.08**<br>**RR: 0.95, CI 0.63-1.44**	Mean change in score<br>Control: -0.4<br>Intervention: -3.87<br>**Change: -3.47 p=0.002***<br>**CI -5.5 - -1.4**		
**HADS – Global score**			Mean score<br>Control: 12.0<br>Intervention: 11.7<br>**Change: -0.34 p=0.61**<br>**CI –1.67 – 0.99**	
**GAD 7 - Anxiety**				Mean score 3 months<br>Control: 3.0<br>Intervention: 2.3<br>**Change: -0.7 p=0.50**<br>**CI: -2.91 - 1.42**<br><br>Mean score 6 months<br>Control: 2.7<br>Intervention: 1.8<br>**Change: -0.9 p=0.43**<br>**CI: -3.10 - 1.32**
**Overall metric for comparison**	**MD: -4**	**MD: -3.47**	**MD: -0.34**	**MD: -0.7**

Table 9: Depression outcome measures

Study / Intervention / Measure	Garrouste et al. 2016	Prichard & Newcomb 2015	Curtis et al. 2016
**Intervention**	**IP family conference**	**Hand massage**	**Communication facilitator**
**HADS – Depression**	At 90 days, Median [IQR]<br>Control: 5.5 [1-11.5]<br>Intervention: 2 [0-6]<br>**Change: -3.5 p=0.04***<br><br>% sign. Depress. score > 8<br>Control: 38.6% (n=17)<br>Intervention: 23.8% (n=10)<br>**Change: 14.8% p=0.14**<br>**RR: 0.61, CI 0.31-1.18**	Mean change in score<br>Control: -0.3<br>Intervention: -2.5<br>**Change: -2.2 p=0.10**<br>**CI -0.49 - 4.7**	
**PHQ 9 Depression**			Mean score 3 months<br>Control: 4.9<br>Intervention: 3.1<br>**Change: -1.8 p=0.09**<br>**CI: -3.89 - 0.31**<br><br>Mean score 6 months<br>Control: 4.7<br>Intervention: 2.4<br>**Change: -2.3 p=0.01***<br>**CI: -4.30 - -0.42**
**Overall metric for comparison**	**MD: -3.5**	**MD: -2.2**	**MD: -1.8**

For peer review only - http://bmjopen.bmj.com/site/about/guidelines.xhtml

Table 10: Trauma outcome measures

Study	Garrouste et al. 2016	Curtis et al. 2016	White et al. 2018
**Measure** / **Intervention**	**Interprofessional family conference**	**Communication facilitator**	**Multicomponent family support**
**PDEQ**	Median [IQR]<br>Control: 14.5 [11-23]<br>Intervention: 13 [0-17]<br>**Change: -1.5 p=0.17**		
**IES-R**	At 90 days, Median [IQR]<br>Control: 24 [12.5-45]<br>Intervention: 21 [9-33]<br>**Change: -3 p=0.24**		
**IES - PTSD (0-88)**		Mean score<br>Control: 20.3<br>Intervention: 21.2<br>**Change: 0.90 p=0.49**<br>**CI: 1.66 - 3.47**	
**PCL - PTSD**			Mean score 3 months<br>Control: 31.6<br>Intervention: 29.8<br>**Change: -1.7 p=0.47**<br>**CI: -6.65 - 3.12**<br><br>Mean score 6 months<br>Control: 30.6<br>Intervention: 27.1<br>**Change: -3.5 p=0.056**<br>**CI: -7.12 - 0.09**
**Overall metric for comparison**	**MD: -3**	**MD: 0.9**	**MD: -7.12**

Table 11: Patient outcome measures

Study	Almoosa et al. 2009	White et al. 2018	Dykes et al. 2017
**Measure** / **Intervention**	**CPR discussions**	**Multi family support**	**Patient engagement**
**Death**	Control: 8% (3/39)<br>Intervention: 37% (17/45)<br>**Change: 31%, p<0.05***<br>**RR: 4.91, CI: 1.55-15.51**	Control: 28.5% (249/873)<br>Intervention: 36% (197/547)<br>**Change: 7.5%, p=0.008***<br>**RR: 1.27, CI: 1.09-1.48**	
**Length of stay**		Mean days<br>Control: 13.5<br>Intervention: 10.4<br>**Change: 3.1, p<0.001***<br>**CI: 0.69 - 0.87**	Mean (median) [range]<br>Pre: 4.9 (2) [1-108], n=881<br>Post: 5.0 (2) [1-115, n=904<br>**Change: 0.1 [0], p=0.61**
**Adverse events**			Pre: 59/1000 patient days<br>Post: 42/1000 patient days<br>**Change: 17, % reduction: 29%**<br>**CI: 6.95-27.05, p<0.05***
**Overall metric for comparison**	**RR: 4.91**	**RR: 1.27**	**29%**

For peer review only

For peer review only - http://bmjopen.bmj.com/site/about/guidelines.xhtml

For peer review only

For peer review only - http://bmjopen.bmj.com/site/about/guidelines.xhtml

Figure 1: Flow diagram

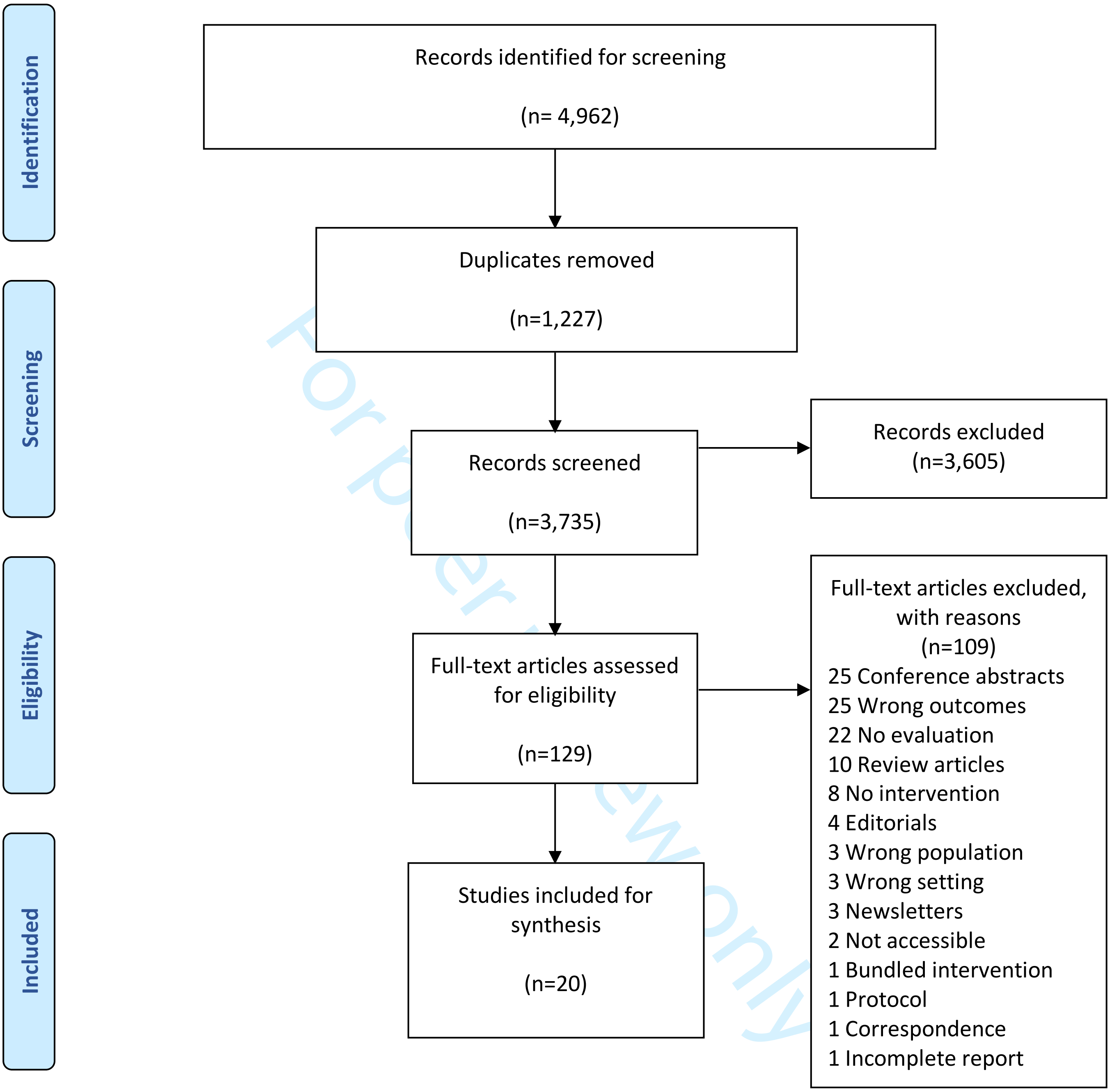

Figure 2: Typology of family involvement interventions.

Features<br>Intervention Types	ICU setting	Family time commitment	Professional input	Opportunities & Challenges	Cost	Evidence level	Engagement outcomes			Clinical outcomes		
							Satisfaction	Decision making	Communication	Wellbeing	Trauma	Patient outcomes
**Environmental unit changes**<br>Unit change interventions consisted of complete structural redesign of an existing ICU with the objective being enhanced family member presence	General	Low	Low	+ Communication<br>+ Interaction<br>- Exposure<br>- Privacy	High	Low	Low impact	Very low impact	Low impact	Very low impact	Very low impact	Very low impact
**Web-based support**<br>Web based and electronic interventions were approaches which utilised information and communication technology to facilitate information sharing and asynchronous communication	General/ specialist	Moderate	Moderate	+ Knowledge<br>+ Efficiency<br>- IT proficiency	Moderate	Moderate	High impact	Low impact	Very low impact	Very low impact	Very low impact	Low impact
**Discussion-based support**<br>Discussion based interventions generally consisted of one off or regular face to face conversations which took place in the unit between family members and healthcare professionals	General/ Specialist	Moderate	High	+ Support<br>+ Wellbeing<br>+ Decision making<br>- Training	Moderate	High	Moderate impact	Very high impact	High impact	Moderate impact	Moderate impact	Low impact
**Multicomponent support**<br>Multicomponent interventions comprised more than one technique to engage, educate and communicate with family members in the ICU	General/ Specialist	High	High	+ Family trauma<br>+ Pt outcomes<br>- Complexity<br>- Resources	High	High	Moderate impact	Very low impact	Low impact	Moderate impact	Low impact	Moderate impact
**Participation in medical rounds**<br>Family involvement in rounds interventions enabled the family members to attend and watch the daily rounds process, with opportunity to raise questions and clarify issues	General ICU	High	Moderate	+ Knowledge<br>+ Efficiency<br>- Time<br>- Satisfaction	Low	High	Moderate impact	Very low impact	High impact	Low impact	Very low impact	Very low impact
**Participation in physical care**<br>Physical participation in care interventions consisted of physical touch between family members and patients being utilised for reciprocal benefit	General ICU	High	Low	+ Satisfaction<br>+ Wellbeing<br>- Confidence	Low	Low	High impact	Very low impact	Very low impact	Moderate impact	Very low impact	Very low impact

Search strategy as applied in Medline (date last run 16.06.2019)

1. exp Critical Illness/
2. exp Critical Care/
3. exp Intensive Care Units/
4. critical care.mp.
5. critical illness*.mp.
6. intensive care.mp.
7. ICU.mp.
8. ITU.mp.
9. OR/1-8
10.exp Patient Participation/
11.patient involve*.mp.
12.patient engage*.mp.
13.shared decision making.mp.
14.patient participat*.mp.
15.service user involve*.mp.
16.family involve*.mp.
17.family participat*.mp.
18.family engage*.mp.
19.caregiver involve*.mp.
20.caregiver engage*.mp.
21.caregiver participat*.mp.
22.relative involve*.mp.
23.relative engage*.mp.
24.relative participat*.mp.
25.OR/11-24
26.9 AND 25

For peer review only

SUPPLEMENTARY FILE

Supplementary Table 1: Search strategy as applied in Medline 1
Supplementary Table 2: Participation in medical rounds interventions 2
Supplementary Table 3: Multi-component family involvement interventions 3
Supplementary Table 4: Discussion based family involvement interventions 3
Supplementary Table 5: Web-based support interventions 5
Supplementary Table 6: Environmental unit changes & Participation in care interventions... 6

Supplementary Table 1: Search strategy as applied in Medline (date last run 16.06.2019)

1. exp Critical Illness/
2. exp Critical Care/
3. exp Intensive Care Units/
4. critical care.mp.
5. critical illness*.mp.
6. intensive care.mp.
7. ICU.mp.
8. ITU.mp.
9. OR/1-8
10. exp Patient Participation/
11. patient involve*.mp.
12. patient engage*.mp.
13. shared decision making.mp.
14. patient participat*.mp.
15. service user involve*.mp.
16. family involve*.mp.
17. family participat*.mp.
18. family engage*.mp.
19. caregiver involve*.mp.
20. caregiver engage*.mp.
21. caregiver participat*.mp.
22. relative involve*.mp.
23. relative engage*.mp.
24. relative participat*.mp.
25. OR/11-24
26. 9 AND 25

For peer review only

Supplementary File: Outcomes grouped by type of family involvement intervention

Supplementary Table 2: Participation in medical rounds interventions

Study	Allen et al. 2017	Jacobowski et al. 2010	Weber et al. 2018
Intervention / Measure	Family on rounds	Family rounds	Family rounds
**Communication (improvement, % agree)**	Pre: n=49 (100%)<br>Post: n=47 (100%)<br>Change 0% p=0.68<br>RR: 1, CI: 0-0		
**Family knowledge**	Improvement, % agree<br>Pre: n=146 (35%) | Post: n=374 (88%)<br>Change 52% p<0.001<br>RR: 2.46, CI: 2.14-2.81		
**Nurse workload**	Reduction, % agree<br>Pre: n=32 (65%) | Post: n=45 (96%)<br>Change 31%<br>RR: 1.46, CI: 1.18-1.81		
**Physician satisfaction**	Pre: n=4 (80%) | Post: n= 5 (83%)<br>Change 3%<br>RR: 1.04, CI: 0.59-1.83		
**Frequency nurse communication**		% highest score<br>Pre: 64% | Post: 57%<br>Change: -7% p=0.30<br>RR: 0.89 CI: 0.70-1.12	
**Frequency doctor communication**		% highest score<br>Pre: 38% | Post: 60%<br>Change: 22% p=0.004<br>RR: 1.60 CI 1.18-2.17	
**Included in decision making**		% highest score<br>Pre: 66% | Post: 76%<br>Change: 10% p=0.12<br>RR: 1.15 CI 0.96-1.38	
**Support in decision making**		% highest score<br>Pre: 49% | Post: 69%<br>Change: 20% p=0.005<br>RR: 1.39 CI 1.09-1.79	
**Time to ask questions**		% highest score<br>Pre: 40% | Post: 23%<br>Change: -17% p=0.02<br>RR: 0.57 CI 0.37-0.90	
**FS ICU 24 - Frequency doctor communication**			% top scores<br>Control: 43%<br>Intervention: 56%<br>Change: 13% p>0.05<br>RR: 1.31 CI 0.90-1.89
**FS ICU 24 - Frequency nurse communication**			% top scores<br>Control: 62%<br>Intervention: 78%<br>Change: 16% p>0.05<br>RR: 1.24 CI 0.98-1.58
**FS-ICU (24) - Decision making**			Mean score (SD)<br>Control: 85.1 (16.3)<br>Intervention: 88.6 (14.6)<br>Change: 3.5 p=0.20<br>CI: -1.98-8.98
**FS-ICU (24) - Global score**			Mean score (SD)<br>Control: 86.0 (16.0)<br>Intervention: 90.8 (10.7)<br>Change: 4.8 p=0.20<br>CI: -0.12 - 9.72
**Support given (emotional)**			% top scores<br>Control: 54%<br>Intervention: 71%<br>Change: 17% p>0.05<br>RR: 1.32 CI 0.99-1.75
**Coordination of care**			% top scores<br>Control: 68%<br>Intervention: 77%<br>Change: 9%<br>RR: 1.13 CI 0.91-1.41

For peer review only

Supplementary Table 3: Multi-component family involvement interventions

Study / Intervention / Measure	Cray 1989	Davidson et al. 2010	Marshall et al. 2016	White et al. 2018
**Intervention**	**Family programme**	**Facilitated sensemaking**	**Nutrition intervention**	**Multi family support**
**Satisfaction with family involvement**	100% agreed (76/76)	Observations showed family members were most engaged when receiving information about how to participate at the bedside.	Reports of increased family involvement, enabling patient advocacy.	
**Helpful in understanding loved one's illness**	93% agreed (71/76)		Improvement in knowledge regarding nutritional needs.	
**HADS - global score (0-42)**				Mean score<br>Control: 12.0<br>Intervention: 11.7<br>Change: -0.34 p=0.61<br>CI −1.67 - 0.99
**QOC - clinician-family communication (0-100)**				Mean score<br>Control: 62.7<br>Intervention: 69.1<br>Change: 6.39 p=0.001<br>CI 2.57 - 10.20
**PPPC - Patient Perception of Patient Centeredness (1-4)**				Mean score<br>Control: 1.8<br>Intervention: 1.7<br>Change: -0.15 p=0.006<br>CI −0.26 - −0.04
**Length of stay - Hospital**				Mean score<br>Control: 6.7<br>Intervention: 7.4<br>Change: 0.90 p=0.045<br>CI 0.81 - 1.00
**Length of stay - ICU**				Mean score<br>Control: 13.5<br>Intervention: 10.4<br>Change: 0.77 p<0.001<br>CI 0.69 - 0.87
**IES - PTSD (0-88)**				Mean score<br>Control: 20.3<br>Intervention: 21.2<br>Change: 0.90 p=0.49<br>CI −1.66 - 3.47

Supplementary Table 4: Discussion based family involvement interventions

Study / Intervention / Measure	Almoosa et al. 2009	Hollman et al. 2018	Garrouste et al. 2016	Huang et al. 2018	Curtis et al. 2016	Shaw et al. 2014
**Intervention**	**CPR discussions**	**Health conversations**	**Interprofessional family conference**	**Primary physician involvement**	**Communication facilitator**	**Team training**
**Satisfaction with CPR Discussion**	Questions answered 84% (36/43) CI 70-91<br>Facilitated decision 77% (33/42) CI 64-88					
**Time to decision**	Days mean (sd)<br>Control 7.4 (3.2)<br>Intervention 6.6 (6.5)<br>Change -0.8 days<br>CI -1.48-3.08					
**Death**	Control 8% (3/39)<br>Intervention 37% (17/45)<br>Change 31%<br>RR 4.91 CI 1.55-15.51					
**Change to less aggressive preference**	Control 18% (8/39)<br>Intervention 25% (11/45)<br>RR: 1.19 CI 0.53-2.66					
**Inclusion in decision making**		Qualitative findings of greater family engagement with ICU nurses.		% completely satisfied<br>Control: 61.4%<br>Intervention: 75.9%<br>Change: 14.5% p=0.05<br>RR: 1.23 CI 1.03-1.49		

For peer review only

Study / Intervention / Measure	Almoosa et al. 2009<br>CPR discussions	Hollman et al. 2018<br>Health conversations	Garrouste et al. 2016<br>Interprofessional family conference	Huang et al. 2018<br>Primary physician involvement	Curtis et al. 2016<br>Communication facilitator	Shaw et al. 2014<br>Team training
**HADS - anxiety**			At 90 days, Median [IQR]<br>Control: 8 [4.5-12]<br>Intervention: 4 [1-9]<br>Change: 4 p=0.01<br><br>% sign. Anxiety (>8)<br>Control: 52.3% (n=23)<br>Intervention: 33.3% (n=14)<br>Change: 19% p=0.08<br>RR: 0.95, CI 0.63-1.44			
**HADS - depression**			At 90 days, Median [IQR]<br>Control: 5.5 [1-11.5]<br>Intervention: 2 [0-6]<br>Change: -3.5 p=0.04<br><br>% sign. Depress. score > 8<br>Control: 38.6% (n=17)<br>Intervention: 23.8% (n=10)<br>Change: 14.8% p=0.14<br>RR: 0.61, CI 0.31-1.18			
**PDEQ**			Median [IQR]<br>Control: 14.5 [11-23]<br>Intervention: 13 [0-17]<br>Change: -1.5 p=0.17			
**IES-R**			At 90 days, Median [IQR]<br>Control: 24 [12.5-45]<br>Intervention: 21 [9-33]<br>Change: -3 p=0.24			
**FS-ICU (24) - ICU Care**				Mean (sd)<br>Control: 88.38 (14.5)<br>Intervention: 90.22 (12.68)<br>Change: 1.84 p=0.28<br>CI -2.42 - 6.09		
**FS-ICU (24) - Global score**				Mean (sd)<br>Control: 84.91 (12.17)<br>Intervention: 86.4 (11.76)<br>Change: 1.49 p=0.16<br>CI -2.14 - 5.12		
**FS-ICU (24) - Decision making**				Mean (sd)<br>Control: 80.07 (12.76)<br>Intervention: 81.06 (15.1)<br>Change: 0.99 p=0.16<br>CI -3.00 - 4.98		
**Control over patient care**				% completely satisfied<br>Control: 55.6%<br>Intervention: 73.6%<br>Change: 18% p=0.02<br>RR: 1.31 CI 1.07-1.61		
**GAD 7 - anxiety**					Mean score 3 months<br>Control: 3.0<br>Intervention: 2.3<br>Change: -0.7 p=0.50<br>CI: -2.91 - 1.42<br><br>Mean score 6 months<br>Control: 2.7<br>Intervention: 1.8	

4

For peer review only

Study / Measure	Almoosa et al. 2009	Hollman et al. 2018	Garrouste et al. 2016	Huang et al. 2018	Curtis et al. 2016	Shaw et al. 2014
Intervention	CPR discussions	Health conversations	Interprofessional family conference	Primary physician involvement	Communication facilitator	Team training
					Change: -0.9 p=0.43<br>CI: -3.10 - 1.32	
**PHQ 9 - depression**					Mean score 3 months<br>Control: 4.9<br>Intervention: 3.1<br>Change: -1.8 p=0.09<br>CI: -3.89 - 0.31<br><br>Mean score 6 months<br>Control: 4.7<br>Intervention: 2.4<br>Change: -2.3 p=0.01<br>CI: -4.30 - -0.42	
**PCL - PTSD**					Mean score 3 months<br>Control: 31.6<br>Intervention: 29.8<br>Change: -1.7 p=0.47<br>CI: -6.65 - 3.12<br><br>Mean score 6 months<br>Control: 30.6<br>Intervention: 27.1<br>Change: -3.5 p=0.056<br>CI: -7.12 - 0.09	
**Staff confidence. All items improved, biggest change: confidence in encouraging family presence**						Mean score (SD)<br>Pre: 7.0 (1.7)<br>Post: 8.4 (1.1)<br>Change: 1.4<br>p<0.001<br>CI: 0.81-1.99
**FS ICU 24 - Frequency nurse communication**						Mean score<br>Pre: 79.2<br>Post: 87.18<br>Change: 7.98<br>p=0.04
**FS ICU 24 - Frequency doctor communication**						Mean score<br>Pre: 67.86<br>Post: 76.69<br>Change: 8.83<br>p=0.04
**FS ICU 24 - Honesty of information**						Mean score<br>Pre: 77.78<br>Post: 87.08<br>Change: 9.30<br>p=0.01
**FS-ICU (24) - Decision making**						Mean score<br>Pre: 77.47<br>Post: 83.32<br>Change: 5.85<br>p=0.05
**FS-ICU (24) - Global score**						Mean score<br>Pre: 83.21<br>Post: 85.69<br>Change: 2.48<br>p=0.32

Supplementary Table 5: Web-based support interventions

Study / Measure	Dalal et al. 2015	Dykes et al. 2017	Ernecoff et al. 2016	Huffines et al. 2013
Intervention	Patient centred toolkit	Patient engagement ICT	Tablet decision support	Support care algorithm
**Usability**	Mean (sd) usability score<br>m 74 (16.7)			
**Satisfaction**	Satisfaction 72% (13/18)<br>Mean (sd) satisfaction score<br>4.06 (0.94)			

For peer review only

Study / Intervention / Measure	Dalal et al. 2015	Dykes et al. 2017	Ernecoff et al. 2016	Huffines et al. 2013
Intervention	Patient centred toolkit	Patient engagement ICT	Tablet decision support	Support care algorithm
HCAHPS		% top score 9-10<br>Pre: 71.8%, n=53, CI 61.1-82.6<br>Post: 93.3%, n=58, CI 88.2-98.4<br>Change 21.5<br>RR 1.33 CI 1.10-1.55		
FS-ICU (20)		Mean score<br>Pre: 84.3, n=106, CI 81.3-87.3<br>Post: 90, n=156, CI 88.1-91.9<br>Change: 5.7, CI 2.31-9.09		
Adverse events		Pre: 59/1000 patient days<br>Post: 42/1000 patient days<br>Change 17, CI 6.95-27.05<br>Relative reduction 29%		
Length of stay		Mean (median) LoS [range]<br>Pre: 4.9 (2) [1-108], n=881<br>Post: 5.0 (2) [1-115, n=904<br>p=0.61		
Share in decisions about care planning			Qualitative perceptions of improving family engagement, communication and involvement in decision making.	Mean satisfaction (1-4)<br>Pre: 2.97 | Post: 3.41<br>Change: 0.44 p=0.07<br><br>% scoring excellent<br>Pre: 45% | Post: 68%<br>Change: 23% p=0.009<br>RR: 1.52 CI 1.03-2.24
Support and encouragement given				Mean satisfaction (1-4)<br>Pre: 3.38 | Post: 3.52<br>Change: 0.15 p=0.47<br><br>% scoring excellent<br>Pre: 60% | Post 75%<br>Change: 15% p=0.14<br>RR: 1.23 CI 0.91-1.65
Nurses and doctors working together as a team				Mean satisfaction (1-4)<br>Pre: 3.38 | Post: 3.73<br>Change: 0.35 p=0.04<br><br>% scoring excellent<br>Pre: 64% | Post: 83%<br>Change: 19% p=0.04<br>RR: 1.31 CI 1.00-1.71

Supplementary Table 6: Environmental unit changes & Participation in care interventions

Study	Choi et al 2013	Rippin et al. 2015	Prichard & Newcomb 2015
	Environmental unit changes		Physical participation in care
Intervention / Measure	PFC Unit design	Family centred design	Hand massage
Family interaction with patient	r=0.632 | p<0.01		
Family interaction with staff	r=0.552 | p<0.01	Qualitative observations of increased, family-initiated interactions.	
HADS - anxiety			Mean change in score<br>Control: -0.4<br>Intervention: -3.87<br>Change: -3.47 p=0.002<br>CI -5.5 - -1.4
HADS - depression			Mean change in score<br>Control: -0.3<br>Intervention: -2.5<br>Change: -2.2 p=0.10<br>CI -0.49 - 4.7

For peer review only - http://bmjopen.bmj.com/site/about/guidelines.xhtml

Reporting checklist for systematic review and meta-analysis.

Based on the PRISMA guidelines.

Instructions to authors

Complete this checklist by entering the page numbers from your manuscript where readers will find each of the items listed below.

Your article may not currently address all the items on the checklist. Please modify your text to include the missing information. If you are certain that an item does not apply, please write "n/a" and provide a short explanation.

Upload your completed checklist as an extra file when you submit to a journal.

In your methods section, say that you used the PRISMAreporting guidelines, and cite them as:

Moher D, Liberati A, Tetzlaff J, Altman DG, The PRISMA Group. Preferred Reporting Items for Systematic Reviews and Meta-Analyses: The PRISMA Statement

For peer review only

		Reporting Item	Page Number
**Title**			
	#1	Identify the report as a systematic review, meta-analysis, or both.	1
**Abstract**			
Structured summary	#2	Provide a structured summary including, as applicable: background; objectives; data sources; study eligibility criteria, participants, and interventions; study appraisal and synthesis methods; results; limitations; conclusions and implications of key findings; systematic review registration number	2
**Introduction**			
Rationale	#3	Describe the rationale for the review in the context of what is already known.	4
Objectives	#4	Provide an explicit statement of questions being addressed with reference to participants, interventions, comparisons, outcomes, and study design	5

		(PICOS).	
**Methods**			
Protocol and registration	#5	Indicate if a review protocol exists, if and where it can be accessed (e.g., Web address) and, if available, provide registration information including the registration number.	5
Eligibility criteria	#6	Specify study characteristics (e.g., PICOS, length of follow-up) and report characteristics (e.g., years considered, language, publication status) used as criteria for eligibility, giving rational	5
Information sources	#7	Describe all information sources in the search (e.g., databases with dates of coverage, contact with study authors to identify additional studies) and date last searched.	5
Search	#8	Present full electronic search strategy for at least one database, including any limits used, such that it could be repeated.	5
Study selection	#9	State the process for selecting studies (i.e., for screening, for determining eligibility, for inclusion in the systematic review, and, if applicable, for inclusion in the meta-analysis).	5-6
Data collection process	#10	Describe the method of data extraction from reports (e.g., piloted forms, independently by two reviewers) and any processes for obtaining and confirming data from investigators.	5-6
Data items	#11	List and define all variables for which data were sought (e.g., PICOS, funding sources), and any assumptions and simplifications made.	5-6
Risk of bias in individual studies	#12	Describe methods used for assessing risk of bias in individual studies (including specification of whether this was done at the study or outcome level, or both), and how this information is to be used in any data synthesis.	5-6
Summary measures	#13	State the principal summary measures (e.g., risk ratio, difference in means).	5-6
Planned methods of analyis	#14	Describe the methods of handling data and combining results of studies, if done, including measures of consistency (e.g., I2) for each meta-analysis.	6
Risk of bias across studies	#15	Specify any assessment of risk of bias that may affect the cumulative evidence (e.g., publication bias, selective reporting within studies).	5-6
Additional	#16	Describe methods of additional analyses (e.g., sensitivity or subgroup	6

For peer review only

analyses		analyses, meta-regression), if done, indicating which were pre-specified.	
**Results**			
Study selection	#17	Give numbers of studies screened, assessed for eligibility, and included in the review, with reasons for exclusions at each stage, ideally with a flow diagram.	6
Study characteristics	#18	For each study, present characteristics for which data were extracted (e.g., study size, PICOS, follow-up period) and provide the citation.	6-7
Risk of bias within studies	#19	Present data on risk of bias of each study and, if available, any outcome-level assessment (see Item 12).	7
Results of individual studies	#20	For all outcomes considered (benefits and harms), present, for each study: (a) simple summary data for each intervention group and (b) effect estimates and confidence intervals, ideally with a forest plot.	7-8
Synthesis of results	#21	Present the main results of the review. If meta-analyses are done, include for each, confidence intervals and measures of consistency.	8-9
Risk of bias across studies	#22	Present results of any assessment of risk of bias across studies (see Item 15).	9-10
Additional analysis	#23	Give results of additional analyses, if done (e.g., sensitivity or subgroup analyses, meta-regression [see Item 16]).	9-10
**Discussion**			
Summary of Evidence	#24	Summarize the main findings, including the strength of evidence for each main outcome; consider their relevance to key groups (e.g., health care providers, users, and policy makers	10-11
Limitations	#25	Discuss limitations at study and outcome level (e.g., risk of bias), and at review level (e.g., incomplete retrieval of identified research, reporting bias).	10-11
Conclusions	#26	Provide a general interpretation of the results in the context of other evidence, and implications for future research.	11
**Funding**			
Funding	#27	Describe sources of funding or other support (e.g., supply of data) for the systematic review; role of funders for the systematic review.	11

For peer review only

The PRISMA checklist is distributed under the terms of the Creative Commons Attribution License CC-BY.
This checklist was completed on 08. July 2020 using https://www.goodreports.org/, a tool made by the EQUATOR Network in collaboration with Penelope.ai

For peer review only
